# Harnessing Multiscale Topographic Environmental Variables for Regional Coral Species Distribution Models

**DOI:** 10.1002/ece3.71292

**Published:** 2025-04-23

**Authors:** Annie S. Guillaume, Renata Ferrari, Oliver Selmoni, Véronique J. L. Mocellin, Hugo Denis, Melissa Naugle, Emily Howells, Line K. Bay, Stéphane Joost

**Affiliations:** ^1^ Geospatial Molecular Epidemiology Group (GEOME), Laboratory for Biological Geochemistry (LGB) École Polytechnique Fédérale de Lausanne (EPFL) Lausanne Switzerland; ^2^ Australian Institute of Marine Science Townsville Queensland Australia; ^3^ Department of Embryology Carnegie Institution for Science Baltimore Maryland USA; ^4^ Department of Plant Biology Carnegie Institution for Science Stanford California USA; ^5^ UMR250/9220 ENTROPIE (IRD‐CNRS‐UR‐IFREMER‐UNC) Noumea Cedex New Caledonia France; ^6^ ED 129, SU Sorbonne Université Paris France; ^7^ National Marine Science Centre, Faculty of Science and Engineering Southern Cross University Coffs Harbour New South Wales Australia

**Keywords:** bathymetry digital elevation model (DEMs), multiscale analyses, seascape ecology, spatial scale, species distribution modelling (SDM), terrain attributes

## Abstract

Effective biodiversity conservation requires knowledge of species' distributions across large areas, yet prevalence data for marine sessile species is scarce, with traditional variables often unavailable at appropriate temporal and spatial resolutions. As marine organism distributions generally depend on terrain heterogeneity, topographic variables derived from digital elevation models (DEMs) can be useful proxies in ecological modelling, given appropriate spatial resolutions. Here, we use three reef‐building *Acropora* coral species across the Great Barrier Reef, Australia, in a case study to (1) assess high‐resolution bathymetry DEM sources for accuracy, (2) harness their derived topographic variables for regional coral species distribution models (SDMs), and (3) develop a transferable framework to produce, select and integrate multi‐resolution variables into marine spatial models. For this, we obtained and processed three distinct bathymetric digital depth models that we treat as DEMs, which are available across the GBR extent: (i) Allen Coral Atlas (ACA) at 10 m, (ii) DeepReef at 30 m and (iii) DeepReef at 100 m. We generalised the three DEMs to multiple nested spatial resolutions (15 m–120 m) and derived the same eight topographic variables to assess SDM sensitivity to bathymetry source and spatial resolution. The ACA and DeepReef DEMs shared similar vertical accuracies, each producing topographic variables relevant to marine SDMs. Slope and vector ruggedness measure (VRM), capturing hydrodynamic movement and shelter or exposure, were the most relevant variables in SDMs of all three species. Interestingly, variables at the finest resolution (15 m) were not always the most relevant for producing accurate coral SDMs, with optimal resolutions between 15 and 60 m depending on the variable type and species. Using multi‐resolution topographic variables in SDMs provided nuanced insights into the multiscale drivers of regional coral distributions. Drawing from this case study, we provide a practical and transferable framework to facilitate the adoption of multiscale SDMs for better‐informed conservation and management planning.

## Graphical abstract

We investigate the application of multiscale topographic variables obtained from freely available bathymetric digital elevation models (DEMs) for ecological modelling in seascape environments. Using a case study of three *Acropora* coral species in the Great Barrier Reef, we demonstrate that various spatial resolutions can effectively model coral distributions, thus allowing us to identify the key topographic variables in these models. Integrating multiscale frameworks into generating environmental predictor variables has the potential to improve ecological models, supporting the development of robust and effective marine conservation and management planning.

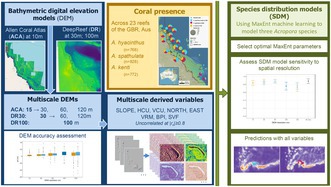

## 1 Introduction

Changing oceanic conditions are placing many populations of reef‐building corals at extinction risk globally (Hughes et al. 2017; Ortiz et al. 2021; Otto 2018), calling for targeted spatial biodiversity conservation and management plans to protect keystone species of reef ecosystems (Goetze et al. 2021; Pittman et al. 2021). Over the last 25 years, considerable effort has been made to harness remote‐sensed satellite imagery for producing high‐resolution geomorphic and benthic habitat maps of coral reefs globally. Examples of open‐source resources include the Millennium Coral Reef Mapping Project (Andréfouët and Bionaz 2021; Andréfouët and Riegl 2004), the Khaled bin Sultan Living Oceans Foundation (Carlton et al. 2021; Purkis et al. 2019), Reef Cover (Kennedy et al. 2021) and the Allen Coral Atlas (ACA; Allen Coral Atlas 2022; Lyons et al. 2024). Whilst such coral habitat maps provide valuable insights into coral reef diversity and health for conservation outcomes (e.g., Bakker et al. 2024; Lyons et al. 2024; Roelfsema et al. 2020), managers and researchers often require species‐specific distribution maps for fieldwork and species‐targeted management plans. To this end, species distribution models (SDMs; Elith and Leathwick 2009) are simple and powerful tools that can help predict the location of target species across large areas (Winship et al. 2020), offering a streamlined and cost‐effective approach to identify priority areas for research and management intervention.

Accurate and informative SDMs require incorporating environmental predictor variables that are expected to influence the fitness, and therefore distribution, of the species being modelled (Winship et al. 2020; Yuen et al. 2023). When modelling coral distributions, oceanographic and climatic variables are expected to be important predictors (Lukoschek et al. 2016; Lyons et al. 2020; McClanahan and Azali 2021) as they capture conditions that can constrain coral development and growth (e.g., temperature, light, water quality, pH and benthic composition; Abrego et al. 2021; Cornwall et al. 2021; Donovan et al. 2021; Pratchett et al. 2015; Vega Thurber et al. 2020). However, such variables are rarely available at fine‐enough temporal and spatial resolutions required to model sessile marine organisms (Lecours, Brown, et al. 2016; McArthur et al. 2010). Indeed, they are typically available at either 30 m resolution spanning only the last decade (e.g., Zuo et al. 2023) or at 1–5 km resolutions spanning multiple decades (e.g., Selmoni et al. 2023). Alternatively, high‐resolution, point‐measured conditions on reefs can be obtained, but these require mathematical interpolations to obtain values across the entire target study extent (e.g., Colberg et al. 2020; Devlin et al. 2013). Given the limitations of these more ‘traditional’ variables, topographic variables representing terrain heterogeneity at finer spatial resolutions (≤ 30 m) could provide convenient alternatives for distribution modelling of marine sessile organisms across regional extents (Bongaerts et al. 2021; Duce et al. 2016; Lecours, Dolan, et al. 2016; Lepczyk et al. 2021; Pittman et al. 2021).

Fine‐scale topographic variables derived from digital elevation models (DEMs) represent seascape heterogeneity, which dictates important environmental conditions that directly affect habitat suitability for sessile marine organisms (such as corals), including surface attachment sites, micro‐climates and nutrient availabilities (Kool et al. 2013; Lecours, Dolan, et al. 2016; Pygas et al. 2020; Tokeshi and Arakaki 2012). Topographic variables are therefore expected to be important metrics driving adaptation, distribution and physiology of sessile organisms, including corals, that need to be explicitly considered in modelling for reef conservation. Though topographic variables were originally developed to model geological and hydrological processes of terrestrial systems (Wilson and Gallant 2000), they have also been used to represent important marine ecological patterns and processes (Harris and Baker 2012; Lecours, Brown, et al. 2016; McArthur et al. 2010). A plethora of DEM‐derived variables have been developed to capture marine topographic structure, notably to model seabed roughness and structural complexity (see Lecours, Devillers, Simms, et al. 2017; Pygas et al. 2020; Smith 2014; Sous et al. 2024; Wilson and Gallant 2000). Such variables have been used, alone or alongside oceanographic variables, to accurately model benthic biota distributions (e.g., Bridge et al. 2012; Tong et al. 2016; Winship et al. 2020; Yuen et al. 2023).

In the present study, we selected a combination of eight easily computable terrain attributes commonly used in the marine and terrestrial literature (Table 1). First, the primary terrain attributes of slope, curvature (both horizontal and vertical) and aspect (as northness and eastness) are directly calculated from DEMs (Böhner et al. 2002; Wilson and Gallant 2000). These variables were selected to capture processes such as energy modification across terrain, affecting water velocity (Moore et al. 1991; Wilson et al. 2007), erosion and deposition rates (Lecours, Dolan, et al. 2016; Wilson and Gallant 2000), exposure to currents and wind (Lecours, Dolan, et al. 2016; Wilson and Gallant 2000; Wilson et al. 2007), and solar radiation levels (Moore et al. 1991; Pygas et al. 2020). From these, more complex secondary terrain attributes are derived that are specifically designed to describe a given pattern as a function of a process (Wilson and Gallant 2000). For instance, we derived terrain complexity variables, including vector ruggedness measure (VRM; Sappington et al. 2007) and bathymetric position index (BPI; Lundblad et al. 2006), to capture the three‐dimensional physical environment, where VRM quantifies local variation in terrain independent of slope (Sappington et al. 2007) and BPI captures broad terrain variation, such as crests and channels (Deng et al. 2007; Montesano et al. 2017; Pygas et al. 2020). These variations influence processes such as hydrodynamic movement, shelter or exposure, attachment sites and turbulence (Pygas et al. 2020), where VRM and BPI are associated with benthic fauna density in marine environments (Graham and Nash 2013; Price et al. 2021). We also derive sky view factor (SVF), a common metric used in modelling sessile terrestrial species distributions (e.g., Guillaume et al. 2021) that captures processes of light attenuation and exposure to the surrounds (Häntzschel et al. 2007). Alongside depth, these eight variables are expected to capture a majority (> 70%) of topographic structures required when modelling species‐environmental relationships (Lecours, Devillers, Simms, et al. 2017).

**TABLE 1:** Description and interpretation of bathymetric digital elevation models (DEM) and eight independent variables derived from three open‐source bathymetric models, the Allen Coral Atlas (ACA at 10 m) and DeepReef (DR at 30 m and 100 m).

	Variable	Abbreviation	Description	*A. hyacinthus*	*A. spathulata*	*A. kenti*	Overall optimal
Primary terrain attributes	Bathymetry	DEM	DEM of the bathymetry obtained from Allen Coral Atlas (Allen Coral Atlas 2022) or DeepReef (Beaman 2010). When used as a variable, it is referred to as ‘depth’. In meters below sea level. DEM = 0 → sea level DEM < 0 → below sea level	n/a	15 m ACA+	15 m ACA+	15 m ACA
	Slope	SLOPE	*Morphometry*. Maximum rate of change in DEM. Proxy for water flow, wave direction, wave intensity, erosion, solar radiation, currents, nutrition, etc. Affects habitat formation (e.g., sand, reef flat, reef crests, strata, etc.). In radians. Slope = 0 rad =0° → completely flat surface Slope = 1 rad =57.2° → steep slope	60 m ACA*	60 m ACA+	60 m ACA*	60 m ACA
	Aspect		*Morphometry*. Orientation of the slope in the downward direction, that describes energy modification across terrain: e.g., wave direction, erosion, light, currents. In radians.				
	Eastness	EAST	Represents the sine Aspect. EAST = −1 → west‐facing EAST = 0 → north‐ or south‐facing EAST = 1 → east‐facing	n/a	n/a	n/a	n/a
	Northness	NORTH	Represents the cosine Aspect. NORTH = −1 → south‐facing NORTH = 0 → east‐ or west‐facing NORTH =1 → north‐facing	n/a	n/a	n/a	n/a
	Vertical (profile) curvature	VCU	*Morphometry*. The level of convexity or concavity in parallel to slope. Important for understanding variations in terrain and complexity and quantifying the energy modification across terrain, explaining acceleration or deceleration of flow across terrain. In radians m^−1^. VCU < 0 → upwardly convex VCU = 0 → linear surface VCU > 0 → upwardly concave	30 m ACA*	30 m ACA+	30 m DR*	30 m ACA/DR
	Horizontal (plan) curvature	HCU	*Morphometry*. The level of convexity or concavity perpendicular to slope. Important for understanding variations in terrain and complexity and quantifying the energy modification across terrain explaining convergence or divergence of flow. In radians m^−1^. HCU < 0 → sideward concave HCU = 0 → linear surface HCU > 0 → sideward convex	n/a	n/a	n/a	n/a
Secondary terrain attributes	Vector ruggedness measure	VRM	*Morphometry*. Quantifies local variation in seabed rugosity with less correlation to slope, indicating a combined variability in slope and aspect. Predictor of habitat suitability and biodiversity. No unit. VRM = 0 → no variation in terrain around target VRM = 1 → complete variation in terrain around	30 m DR−	60 m ACA−	60 m ACA−	30–60 m ACA/DR
	Bathymetry Position Index	BPI	*Morphometry*. Indicates a pixel's relative position with regards to surrounding pixels, giving an indication of local topography (peaks and troughs). Calculated using an annulus‐shaped focal window. Predictor of habitat suitability and biodiversity. In meters. BPI < 0 → local lows in terrain (pits) BPI = 0 → flat terrain BPI > 0 → local highs in terrain (peaks)	15 m ACA+	15 m ACA+	15 m ACA+	15 m ACA
	Sky view factor	SVF	*Lighting*. Quantifies the proportion of sky visible from a point around a particular radius, by calculating the ratio of the radiation received by a planar surface to the radiation emitted by the entire hemispheric environment. It uses only the zenith angles above the horizontal plane. No unit. SVF = 0 → light completely obstructed to target SVF = 1 → light completely unobstructed to target	n/a	100 m+	120 m or 15 m−	Variable ACA/DR

*Note:* A summary of the most important spatial resolution and bathymetric sources for each variable is provided for all three *Acropora* species (*A. hyacinthus, A. spathulata* and *A. kenti* ), based on the Jackknife evaluations of the variables used in the MaxEnt SDMs, where ‘top variables’ are those with AUC_ONLY_ > 0.7 (see Figure 6). The symbols denote a positive (+), negative (−), or complex (*) relationship according to variable response curves, and ‘n/a’ indicates that the variable was not a ‘top variable’ in MaxEnt models and therefore its relationship with coral distribution was not assessed. The parameters used for calculations are provided in Table S3 of the Supporting Information.

Accurate derivations of topographic variables require high‐resolution bathymetric DEMs that continuously capture a given study extent (Pygas et al. 2020). The technology used to obtain these DEMs dictates spatial resolution, extent and accuracy (reviewed in Lecours, Dolan, et al. 2016), where a trade‐off between spatial resolution and extent exists. Here, we focus on the world's largest reef system, the Great Barrier Reef (GBR) off the north‐east Australian coast, where the finest resolution regional bathymetry models are available between 10 m and 100 m across an extent of approximately 344,000 km^2^. There are two open‐source regional bathymetric digital depth models available across the GBR, which we treat here as DEMs: the Allen Coral Atlas (ACA; Allen Coral Atlas 2022; Li, Schill, et al. 2019; Li et al. 2021) and the DeepReef projects (Beaman 2010, 2017; Hamylton et al. 2015). These are summarised in Table 2. Briefly, the ACA provides a satellite‐derived bathymetry model available at approximately 10 m resolution across the world's coral reef habitats. This dataset is aggregated from unobstructed surface reflectance images obtained predominantly from the Google Earth Engine Sentinel‐2 satellite, calculated to a median depth of 20 m using an automated algorithm (Li, Knapp, et al. 2019), with the highest accuracy in the top 10 m (Li et al. 2021; Table 2). However, the ACA contains some missing pixels resulting in sometimes patchy coverage. The DeepReef project provides two bathymetric models covering the entire GBR and Coral Sea regions at approximately 30 m resolution (0.0003‐arc degree) and 100 m resolution (0.001‐arc degree). Despite availability at coarser resolutions, the two DeepReef bathymetry models are expected to be of higher accuracy than ACA (Table 2), notably in areas > 10 m depth, as they complement satellite‐derived depth estimates with shipborne deep‐water multibeam and single‐beam sonar, as well as airborne LiDAR (Beaman 2010, 2017, 2020). Both ACA and DeepReef bathymetry DEMs can be used to derive topographic variables for use in regional ecological models of marine organisms. However, despite overlapping extents in the GBR, their relative accuracies across the GBR reefs and suitability of derived variable products have yet to be assessed; a necessary first step prior to use in ecological models for regional marine spatial management.

**TABLE 2:** Summary of the three raw open‐access bathymetric digital elevation model (DEM) sources spanning the Great Barrier Reef (GBR) extent: The Allen Coral Atlas (ACA) and the DeepReef project at 30 m and 100 m resolutions.

	Allen Coral Atlas (ACA)	DeepReef 30 m	DeepReef 100 m
Downloaded spatial res.	~10 m	~30 m (0.0003‐arc degrees)	~100 m (0.001‐arc degrees)
Target spatial res.	15 m (generalised to 30, 60, 120 m)	30 m (generalised to 60, 120 m)	100 m
File format	GeoTIFF	GeoTIFF	adf
Data source	Google Earth Engine Sentinel‐2 satellite – median depth over 12 months	Deep‐water multibeam and single beam sonar surveys, airborne LiDAR bathymetric surveys, and satellite data	Deep‐water multibeam and single beam sonar surveys, airborne LiDAR bathymetric surveys, and satellite data
Approximate accuracy	0‐10 m: RMSE*⋍1.4 m 10‐15 m: RMSE*⋍1.5 m 15‐20 m: RMSE*⋍2.5 m	0‐10 m: TVU^§^ = 1.05 m 10‐15 m: TVU^§^ = 1.18 m 15‐20 m: TVU^§^ = 1.18 m	0‐10 m: TVU^§^ = 1.05 m 10‐15 m: TVU^§^ = 1.18 m 15‐20 m: TVU^§^ = 1.18 m
Website	allencoralatlas.org	pid.geoscience.gov.au/dataset/ga/115066	deepreef.org/2010/07/06/gbr‐bathy/
Accessed	January 2023	May 2023	January 2023
Reference	Allen Coral Atlas (2022), Li et al. (2021)	Beaman (2017)	Beaman (2010)

*Note:* All models represent mean sea level, whilst none have definitive accuracies available (see footnotes). RMSE = Root mean squared error; calculated for the ACA and assessed at Heron Island, southern GBR (Li et al. 2021). § TVU = total vertical uncertainty; reported as the mean TVU across the GBR marine protected area extent using the IHO S‐44 Order 2 formula for the depth categories (International Hydrographic Organization 2020).

The relevance of high‐resolution variables in ecological models depends on their spatial resolutions (Lecours, Devillers, Edinger, et al. 2017; Pradervand et al. 2014). Whilst some recommend using the finest available resolution when possible (Chauvier et al. 2022; Cushman and Landguth 2010; Moudrý et al. 2023), this may not always be appropriate (Carlson et al. 2024; Pygas et al. 2020) owing to computational constraints, the necessity of expert knowledge in manipulating large rasters, and an increased risk of amplifying sampling artefacts from fine‐scale DEM acquisition and processing (Lecours, Devillers, Edinger, et al. 2017). Furthermore, topographic attributes at different spatial resolutions capture distinct environmental aspects that affect species in unique ways (Wood 1996; Woodcock and Strahler 1987). Indeed, optimal resolutions depend on variable type, reef terrain characteristics and the species being modelled (Connor et al. 2018; Guillaume et al. 2021; Tong et al. 2012, 2016). To appropriately capture unique and species‐specific patterns across different spatial resolutions for a given variable, a multiscale framework can be implemented (Guillaume, Leempoel, et al. 2024; Lecours et al. 2015; Misiuk et al. 2021). To do this, a DEM at its finest resolution is generalised to multiple nested resolutions using a Gaussian pyramid algorithm (Kalbermatten et al. 2012; Leempoel et al. 2015), before deriving topographic variables required for the study (Guillaume et al. 2021; Misiuk et al. 2021). Once datasets of multiscale variables are generated, variable selection methods can be used to create sets of species‐specific predictor variables for use in ecological modelling (for more information on this multiscale framework, see Guillaume, Leempoel, et al. 2024). Whilst spatial resolution is well known to impact ecological models, the optimal resolution of variables finer than 100 m for regional distribution models of various coral species remains unknown.

A hotspot for coral conservation research, the expansive GBR offers rich datasets for investigating the relevance of multiscale topographic variables in SDMs. Three high‐resolution open‐source bathymetric DEMs are available across the study extent—the ACA at 10 m and DeepReef at 30 m and 100 m (Table 2). Here, we derive multi‐resolution topographic variables from each DEM source using a multiscale framework and systematically evaluate their relevance in regional SDMs for three dominant reef‐building *Acropora* coral species across the GBR extent. Specifically, we aim to (1) compare three high‐resolution open‐access bathymetry DEMs for their accuracy, (2) harness DEM‐derived topographic variables in regional SDMs for three coral species and (3) develop a transferable framework to produce, select and integrate multi‐resolution topographic variables into marine spatial modelling. Ultimately, we look to assess and develop a framework for producing more nuanced marine spatial ecology models using multiscale variables, which can provide more informed conservation planning for restoration efforts globally.

## 2 Methods

**FIGURE 1:**
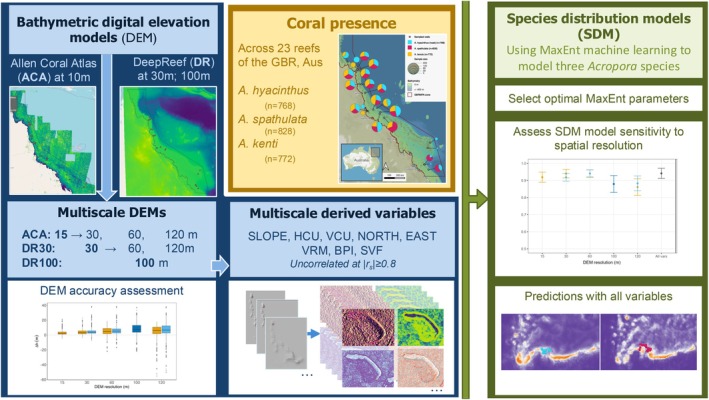
Schema of the workflow used to develop and integrate multiscale topographic variables derived from three bathymetric digital elevation models (DEMs) for use in species distribution modelling (SDM) of three *Acropora* species (*A. hyacinthus, A. spathulata* and *A. kenti* ). Topographic variables were obtained from three open‐access bathymetry models: The Allen Coral Atlas (ACA) at 10 m, and DeepReef (DR) at 30 m and 100 m spatial resolutions, where a multiscale framework was applied to obtain variables at nested spatial resolutions (defined in Table 1). Coral presence‐only coordinates were obtained for each *Acropora* species from across 23 reefs of the Great Barrier Reef, Australia (Figure 2). Topographic variables and presence‐only data were integrated into SDMs using MaxEnt machine learning algorithms to assess performance and produce prediction maps.

A visual schematic of the methods is provided in Figure 1, indicating the workflow followed to model the distribution of three coral species across the GBR using multiscale topographic variables derived from bathymetric DEMs (sourced from the ACA and DeepReef). To avoid ambiguity, we define *spatial resolution* as the pixel size of the variables, and *extent* as the boundary of the region analysed (Anderson et al. 2010). Unless stated otherwise, all analyses were performed in R (v4.1.0, 2021; R Core Team 2021).

### 2.1 Study Species and Sites

**FIGURE 2:**
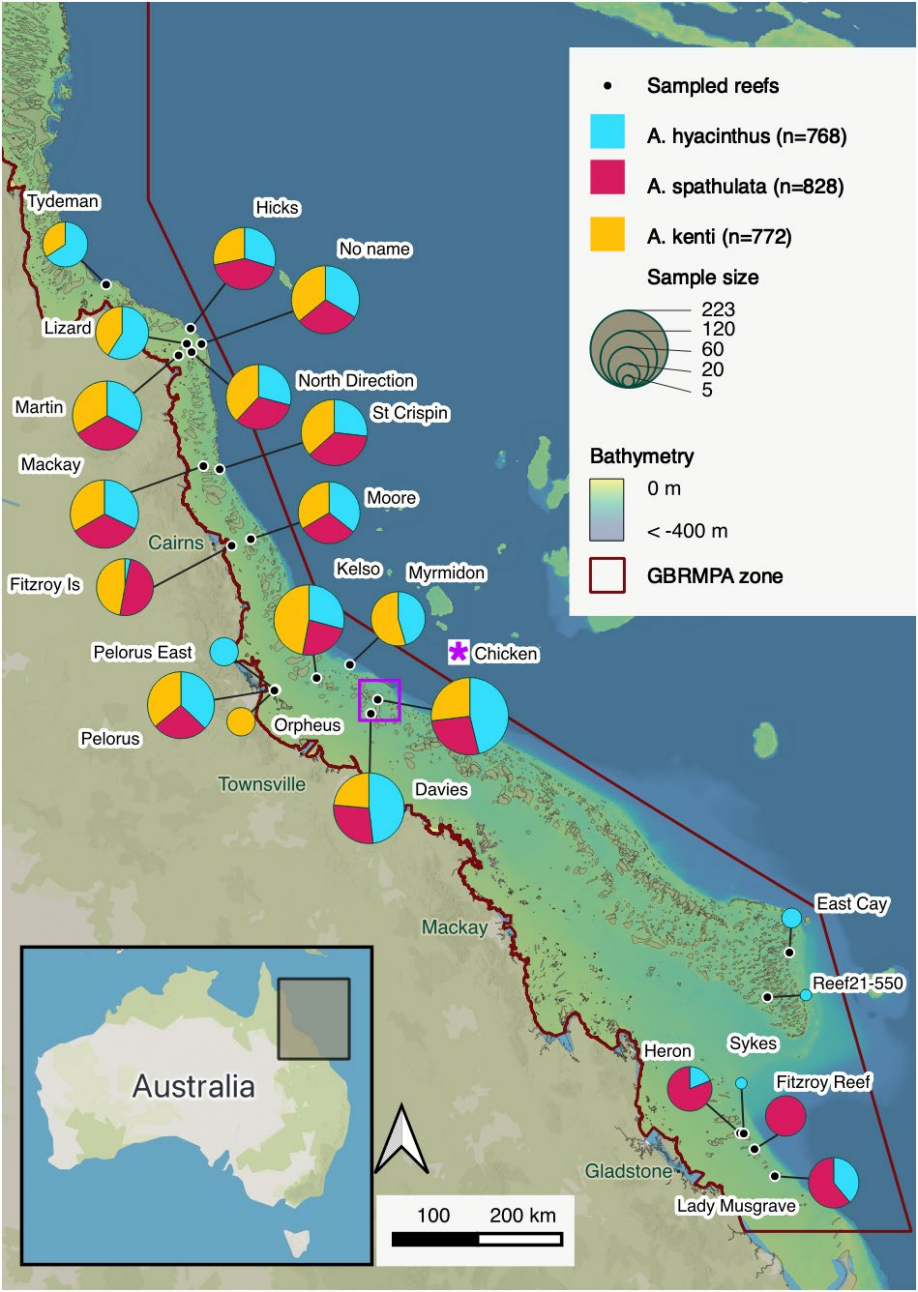
Sampling distribution of three *Acropora* coral species at 23 reefs across the Great Barrier Reef marine protected area (GBRMPA; red outline in EPSG:7855 projection) along the north‐east coast of Australia (insert). Pie charts indicate the proportion of colonies sampled per species ( *A. hyacinthus* , *A. spathulata* and *A. kenti* ) at each reef, where pie chart size reflects the relative sampling intensity at each reef. Raw values are provided in Table S1. The asterisk indicates the location of Chicken Reef, used as an example in Figure 7 (indicated with a purple box). The bathymetric map was obtained from the DeepReef Project at 100 m spatial resolution.

We obtained coral presence‐only data for three reef‐building *Acropora* species dominant at the 23 sampled reefs of the GBR, spread across 1400 km of latitude from varying locations on the reef shelf (Figure 2; Table S1). This biological data comes from the Reef Restoration and Adaptation Program (RRAP; www.gbrrestoration.org), where 3 years of field campaigns were undertaken to collect coral phenotypic and genomic data (Figure 2). Here, we use the GPS location coordinates and the colony depth metadata from the RRAP field campaigns. Sampled reef areas were selected based on expert knowledge of habitat preferences of the three target species. Whilst reef condition was not explicitly collected as part of this study, information can be found on the AIMS reef monitoring website with continual updates (https://apps.aims.gov.au/reef‐monitoring/). Colonies were haphazardly sampled up to a maximum depth of 8 m along reef crests and upper slopes, resulting in either clustered (sampled across 40–75 m) or elongated transects (up to 400 m) of occurrence records per site. Each colony was geolocated in situ for coordinates using a Garmin eTrex10 handheld GPS receiver (2011) towed along the surface by scuba divers, where the GPS gives a location accuracy of approximately 3 m (Fator and Zomrawi 2015). Latitude and longitude were attributed based on photograph timestamps, as per Lukoschek et al. (2016), where the GPS tow line was allowed to drift to a maximum of 2 m above each colony. Coordinates were projected to the GDA2020/MGA zone 55 (EPSG:7855) coordinate system. Colony depth was simultaneously recorded using dive computers (with an expected error of ±1 m; Marlowe et al. 2021) and later standardised to the lowest astronomical tide (LAT) using a custom R script. Tide measurements for sampling dates were obtained from the reef's closest tide station (Table S1; Australian Bureau of Meteorology via tides.willyweather.com.au, accessed May 2023). Tide stations were generally within 17 km of the sample sites, and we expect interpolations to be relatively robust, with three tide stations located further away that could result in larger interpolation errors (Chicken Reef: 39 km; Davies Reef: 46 km; St Crispin: 61 km; Table S1).

Three species of broadcast spawning coral of the genus *Acropora* were investigated here: *A. hyacinthus* (Dana, 1846; *n* = 768 individuals), *A. spathulata* (Brook, 1891; *n* = 828 individuals) and *A. kenti* (Brook, 1892; *n* = 772 individuals; see Bridge et al. 2023) (Tables S1 and S2). These species were selected due to their importance as habitat‐forming species in the GBR (Cooke et al. 2020; Ortiz et al. 2021), where diversity in morphology and habitat preferences makes *Acropora* coral early succession colonisers following disturbance events (Guest et al. 2012; Hazraty‐Kari et al. 2022; Yuen et al. 2023). Furthermore, they are well studied with regards to adaptation, recovery and reef restoration potential (Denis et al. 2024; Hazraty‐kari et al. 2022; Madin et al. 2012; Morais et al. 2023; Quigley et al. 2022; Rose et al. 2021). As *A. hyacinthus* represents a species complex (Ladner and Palumbi 2012), colonies with morphology matching the ‘neat’ putative species were targeted in this study (Naugle et al. 2024).

### 2.2 Bathymetric DEMs

Bathymetric DEM rasters were freely obtained from their online archives, with the ACA at 10 m and DeepReef at 30 m and 100 m (Table 2). These bathymetry models cover the same extents of the GBR, importantly providing depth measurements at the reefs sampled for coral colony data. All DEMs were reprojected and resampled to the GDA2020/MGA zone 55 (EPSG:7855) system at the target resolutions of 30 m and 100 m for DeepReef and 15 m for ACA (the finest resolution for comparison with DeepReef30m), where DeepReef 30 m tiles were first merged into one raster file in QGIS (v3.18.2‐Zürich). The rasters were cropped to a 500 m buffer around each sampled reef to facilitate calculations. The ACA DEM required further processing: pixels were divided by −100 to convert values to meters below sea level, before filling small NA pixel gaps (1.4% of the pre‐processed raster, with seven samples allocated to NA cells at the finest resolution) with the mean of a 3 × 3 window around each NA pixel, filling remaining gaps with the DeepReef30m.

Processed DEMs were generalised to multiple resolutions using a Gaussian pyramid algorithm through the ‘impyramid’ function in MATLAB (MathWorks: MATLAB R2019a, 2019), following Kalbermatten et al. (2012). This produced eight DEMs: ACA15 m at 15 m, 30 m, 60 m and 120 m; DeepReef30 m at 30 m, 60 m and 120 m; and DeepReef100 m at 100 m (Table 2). The maximum resolution of 120 m was restricted by the sampling scheme whilst allowing for comparisons with DeepReef100 m.

All bathymetric DEMs represent Mean Sea Level (MSL) without definitive accuracy measures across the whole GBR extent (but see Table 2 for estimated accuracy values). We assessed vertical error (∆h) by comparing the LAT‐standardised colony depths with the DEM modelled depth (∆h_i_ = in situ–DEM; *n* = 2368; Höhle and Höhle 2009), summarised using the normalised median absolute deviation (NMAD). Outliers were defined as colonies with a |∆h_i_| three times greater than the root mean square error (RMSE; Höhle and Potuckova 2006).

### 2.3 Derived Topographic Variables

We derived eight topographic variables relating to terrain morphometry and light availability from each DEM (Table 1; Ferrari et al. 2018; Lundblad et al. 2006; Price et al. 2021; Tong et al. 2012): slope (SLOPE), eastness (EAST; sine of aspect), northness (NORTH; cosine of aspect), vertical curvature (VCU), horizontal curvature (HCU), vector ruggedness measure (VRM), bathymetry position index (BPI) and sky view factor (SVF). Calculations were performed using ‘MultiscaleDTM’ (Ilich et al. 2023) and ‘RSAGA’ (Brenning et al. 2022; R‐package interface with SAGA GIS v8.4.1: Conrad et al. 2015). Detailed descriptions and calculation parameters are in Table S3. A total of 72 variables were available for downstream models (i.e., one DEM and eight variables obtained from each of the eight resolution‐bathymetry sources).

To compare ACA15 m and DeepReef30 m DEMs and derived variables at the common spatial resolutions of 30 m, 60 m and 120 m, we used scatterplots based on values from 15,000 random points across the 23 sampled reefs.

### 2.4 Regional Species Distribution Modelling Using MaxEnt

The suitability of the eight topographic variables were systematically assessed for each resolution–bathymetric source combination using the MaxEnt SDM algorithm, following the methods of Guillaume et al. (2021). MaxEnt (Phillips et al. 2006) is a machine learning method accessible through ‘maxnet’ in R (Phillips et al. 2017). MaxEnt estimates probability distributions of a species across a user‐defined landscape (Elith et al. 2011; Phillips and Dudík 2008), and automatically handles imbalanced‐biassed occurrence datasets and collinearity amongst predictor variables (Ahmadi et al. 2023; De Marco Júnior and Nóbrega 2018; Feng et al. 2019).

We performed SDMs for each of the three *Acropora* species separately. To reduce model biases from high‐density coral sampling, we thinned occurrence records to retain one coordinate per 15 m pixel (Table S1). Despite thinning, multiple occurrence points remained per grid at coarser resolutions, which we accepted to facilitate comparisons between SDMs at multiple resolutions (Guillaume et al. 2021; Guisan et al. 2007). Pseudo‐absence background coordinates were obtained following MaxEnt best practice guidelines (Phillips and Dudík 2008), with 10,000 randomly selected points from across the 23 reefs, biassed to areas of high coral sampling intensity (Phillips et al. 2009) using all georeferenced coordinates (*n* = 2368). Values from the 72 variables were extracted using the occurrence and background coordinates.

Model parameters were optimised (Merow et al. 2013) by performing MaxEnt with all 72 predictor variables, varying the combination of non‐linear feature class transformation (FC; linear, linear‐product, linear‐quadratic, linear‐product‐quadratic) with regularisation multiplier (RM; 1, 2, 5, 10; Phillips and Dudík 2008). Each FC‐RM combination was run over multiple iterations, using a leave‐one‐reef‐out cross‐validation method for model testing and training, performed in triplicate to randomly divide background points into 75% training and 25% testing. The optimal parameter was selected as the FC‐RM model that minimised the sum of ranks when a performance ranking was assigned for each mean Area Under the Receiver Operating Curve (AUC_TEST_; Bradley 1997) and Bayesian information criteria (BIC; McCullagh and Nelder 1989). A linear‐quadratic FC with an RM of 2 (LQ2) was the top‐ranking parameter combination for all three species (Table S4) and thus used to transform input variables for all following models.

We systematically assessed the impact of spatial resolution and DEM source on MaxEnt model performance, running eight single resolution models for each resolution–bathymetric source combination (*n* = 9 variable types per model) alongside one multi‐resolution model with all variables at all resolutions (*n* = 72 variables, ‘all variable’ model). Ten iterations were run per model to select different background points per cross‐validation. Model significance was assessed for AUC_TEST_ and BIC metrics separately for each species. Models were grouped using the non‐parametric Kruskal–Wallis one‐way analysis of variance and Dunn's post hoc test of pairwise multiple comparisons, applying Holm's correction for multiple testing.

Final MaxEnt models per species used all 72 variables. Jackknife analyses and variable response curves indicated the relative importance of each variable, performed with ‘maxnet’. Prediction maps across the sampled reefs were made using ‘enmSdmX’ (Smith et al. 2023). To assess the prediction map performance, the spatial distribution of presence probabilities were overlaid onto maps of potentially suitable habitat (‘coral/algae’ and ‘rock’) derived from the independent benthic habitat maps of the Allen Coral Atlas (accessed: allencoralatlas.org on 09.02.2023; Allen Coral Atlas 2022).

## 3 Results

### 3.1 Bathymetry DEM Accuracy Assessment

**FIGURE 3:**
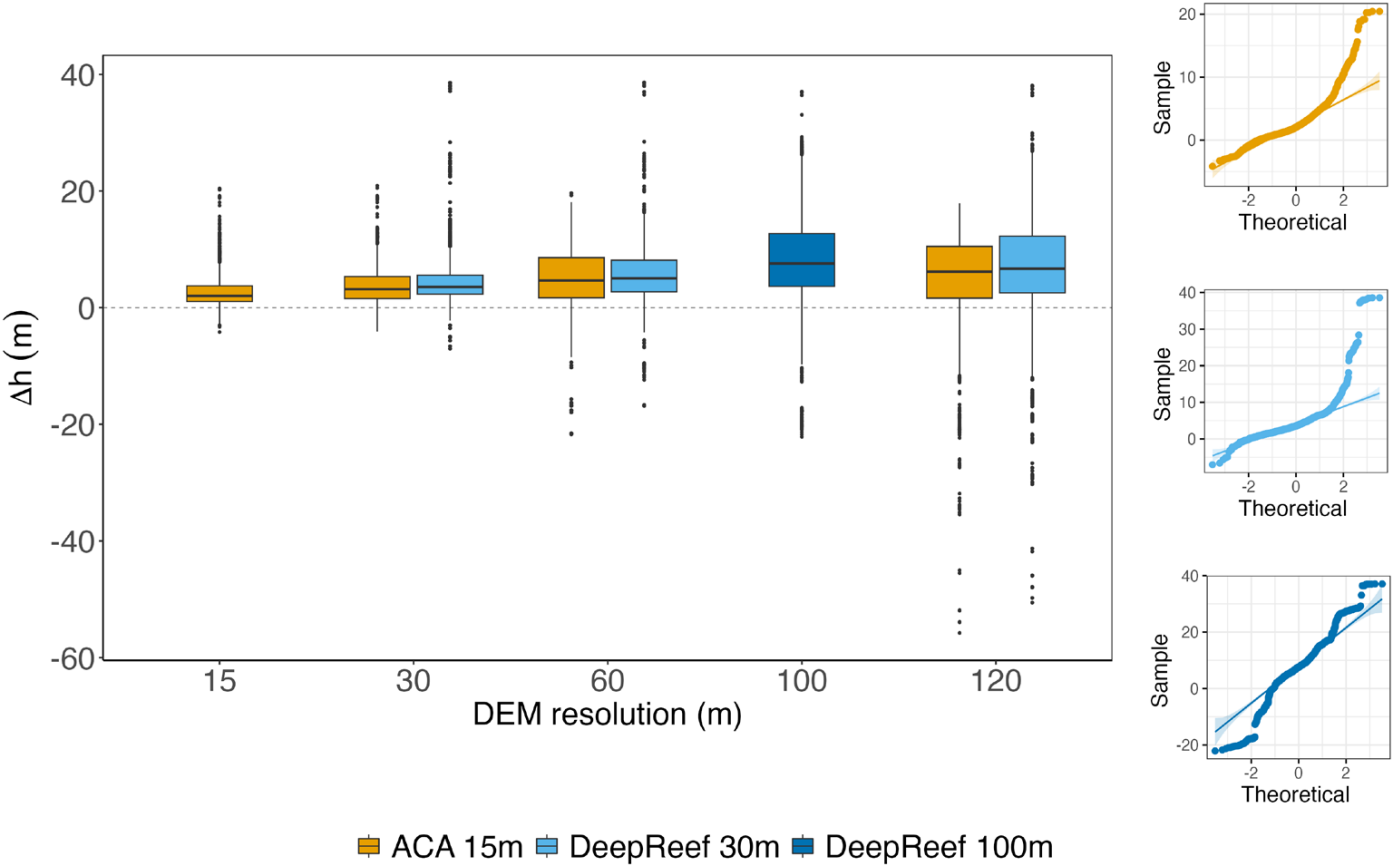
Accuracy of three bathymetry models (Allen Coral Atlas 15 m: Orange; DeepReef 30 m: Light blue; DeepReef 100 m: Dark blue) generalised to multiple spatial resolutions, assessed using sampled coral depths at 23 reefs of the Great Barrier Reef: (a) Boxplots for each bathymetry model highlight vertical error (∆h; in meters), which was calculated as the difference between the tide‐corrected depth of sampled colonies (*n* = 2368) and the predicted depth from the bathymetry models. Quantile‐quantile (Q‐Q) plots were used to assess the normality of ∆h distributions for the (b) Allen Coral Atlas at 15 m resolution, (c) DeepReef 30 m at 30 m resolution, and (d) DeepReef 100 m at 100 m resolution. Here, the theoretical ∆h values that were obtained from a normalised distribution (‘Theoretical’) are plotted against the actual sampled values of ∆h (‘Sampled’). Q‐Q plots of the ∆h for the other spatial resolutions per bathymetry model are available in Figure S1. We note that in situ measurements were corrected to Lowest Astronomical Tide (LAT) whilst the bathymetric models give mean sea level (MSL) values.

Vertical error (∆h) of DEMs compared to in situ sampled depth points revealed a reduction in relative accuracies at larger pixel sizes (Figure 3; exact values provided in Table S5). Q–Q plots of ∆h highlighted non‐normal error distributions, where plots for fine resolution DEMs had strong right skews indicating shallower predictions, whilst plots for coarser resolution DEMs had strong left skews indicating deeper predictions (Figure 3 for base DEMs; Figure S1 for all Q–Q plots). At matching spatial resolutions, ACA and DeepReef DEMs revealed similar relative accuracies, where the DEMs all predicted depths across the GBR as shallower than the in situ coral measurements (Figure 3; Table S5). Median (± standard deviation) errors increased with pixel size for all sources: the ACA DEM at 15 m had 2.0 ± 2.7 m error, 30 m had 3.2 ± 3.2 m, 60 m had 4.7 ± 4.8 m, and 120 m had 6.2 ± 8.9 m. Similarly, the DeepReef DEM at 30 m had 3.6 ± 3.9 m error, 60 m predicted 5.0 ± 5.0 m error, and 120 m predicted 6.7 ± 9.7 m error, whilst the 100 m DeepReef DEM had 7.6 ± 9.3 m. Normalised median absolute deviation (NMAD; visualised in Figure S2; Table S5) highlighted less ∆h variation with DeepReef than ACA at 30 m and 60 m (DeepReef_30m_ = 2.23 vs. ACA_30m_ = 2.66; DeepReef_60m_ = 3.87 vs. ACA_60m_ = 4.97), with the inverse true at 120 m (DeepReef_120m_ = 7.35 vs. ACA_120m_ = 6.55). Similar ∆h variation was seen for the 100 m DEM (DeepReef_100m_ = 6.53).

### 3.2 Derived Topographic Variable Assessment

**FIGURE 4:**
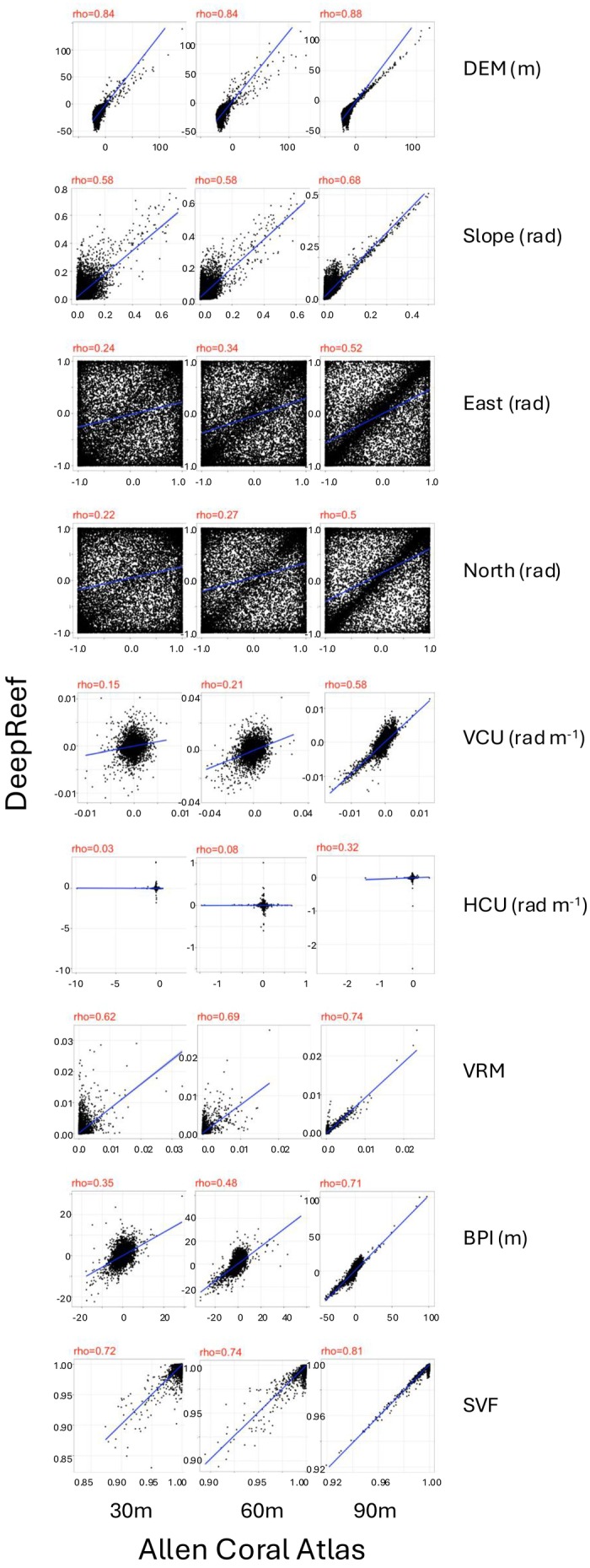
Scatterplots with spearman correlations are used to assess the calculation of eight topographic variables (plot rows, with associated units) derived from two bathymetric digital elevation model (DEMs) sources at the three common resolutions of 30 m, 60 m and 120 m (plot columns). DEMs were obtained from two publicly available sources: Allen Coral Atlas 15 m (ACA15; *x*‐axis), and DeepReef 30 m (*y*‐axis). Values were obtained at 15,000 random points across 23 sampled reefs of the Great Barrier Reef (GBR), selected using the 120 m DEM raster with a limit of one point per pixel. We used Spearman's rho (values above each plot) to compare values derived from ACA and DeepReef, where a straight line along *x* = *y* and rho = 1.0 is expected when DEMs compute the exact same variables, whilst low correlation indicates differences in computed values between DEMs that could potentially impact SDMs performance. Abbreviations and information about variables are provided in Table 1.

The DEMs from the three bathymetric sources and eight topographic variables were generally independent using a Spearman correlation threshold of |r_s_| ≥ 0.8 across the multiple spatial resolutions analysed (heat map summarising correlations in Figure S3). However, strong positive correlations r_s_ ≥ 0.5 were found within variable types for DEPTH, SLOPE, VRM and SVF across all spatial resolutions, and strong negative correlations between multiple resolutions of SVF with VRM and SLOPE. Scatterplots showed strong correlations (r_s_ ≥ 0.84) between the bathymetric models of ACA and DeepReef at the common resolutions of 30 m, 60 m and 120 m (Figure 4). However, the high scatter between topographic variables at matching resolutions from ACA and DeepReef indicates inconsistencies between calculated values, particularly at finer resolutions. This was notable for VCU, HCU, EAST and NORTH, where variables were poorly correlated at 30 m resolutions (r_s_ = 0.15, r_s_ = 0.03, r_s_ = 0.24 and r_s_ = 0.22, respectively), but more consistent at 120 m resolutions (r_s_ = 0.58, r_s_ = 0.32, r_s_ = 0.52 and r_s_ = 0.50, respectively). For SLOPE, VRM, BPI, and SVF, variables were more similar between ACA and DeepReef, with r_s_ > 0.35 at the 15 m resolution and r_s_ > 0.7 at the 120 m resolution.

### 3.3 Predictive Power of Multiscale Topographic Variables for Regional Species Distribution Models

**FIGURE 5:**
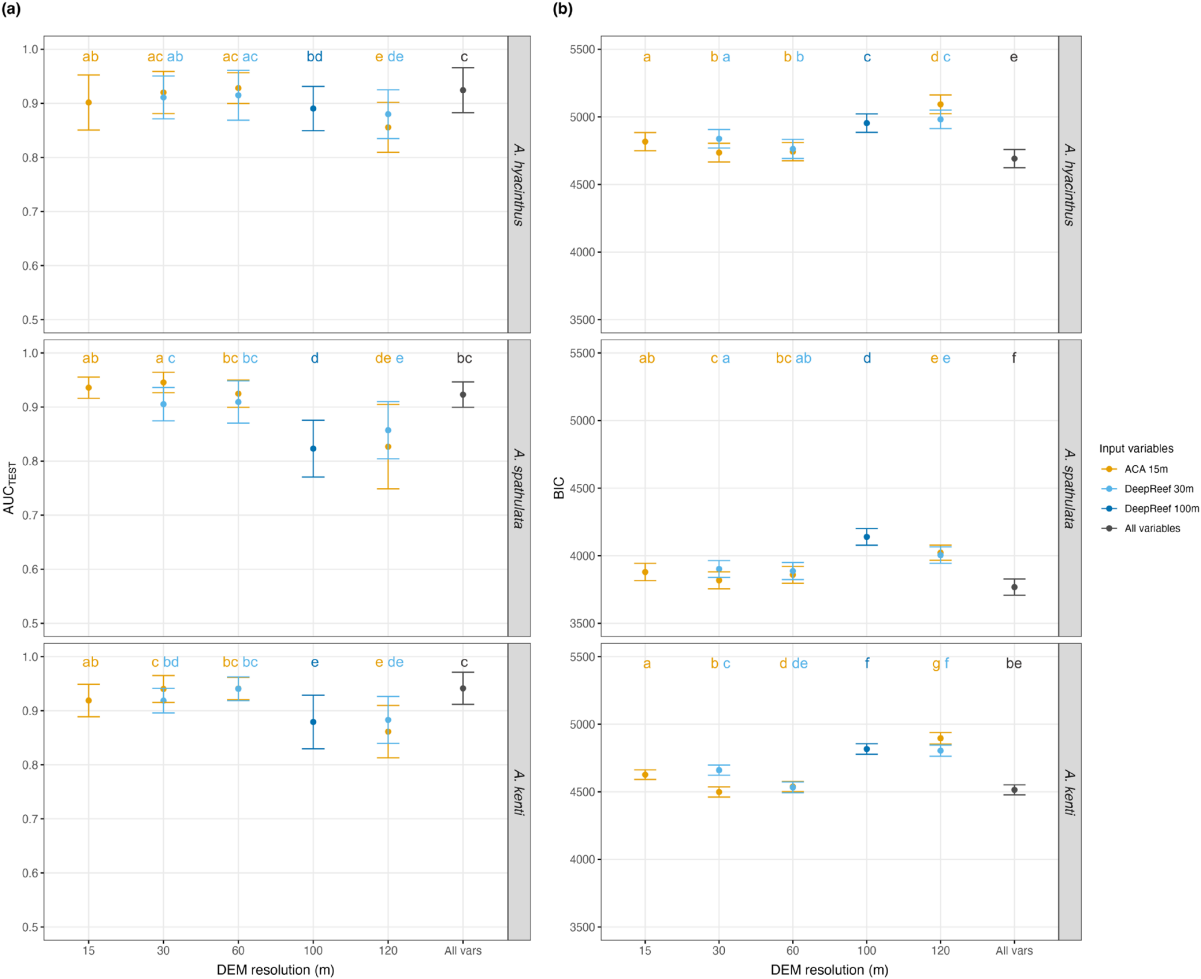
Performance evaluation of MaxEnt species distribution models (SDM) for three *Acropora* species ( *A. hyacinthus* , *A. spathulata* , and *A. kenti* ) on the Great Barrier Reef, using two metrics: (a) Area Under the receiver operating Curve based on the test data (AUC_TEST_) and (b) mean Bayesian Information Criterion (BIC). The *x*‐axes represent the spatial resolution of predictor variables used in SDMs, coloured by the source of DEM bathymetry models: Allen Coral Atlas 15 m (ACA; orange), DeepReef 30 m (light blue), DeepReef 100 m (dark blue). The models built with all variables at all resolutions (*n* = 72; ‘All variables’) are indicated in black. Each point represents the 95% confidence interval of the evaluation criteria for all cross validations repeated over ten iterations. Lettering above each bar (e.g., ab, ac, bd, f, gf, etc.) groups the models with statistically similar performances, assessed using Kruskal‐Wallis tests for multiple comparisons, where the groupings are independent between species and metrics. Full statistical results are available in Table S7.

MaxEnt SDMs using only depth and the eight topographic variables at multiple resolutions demonstrated extremely high predictive capabilities for all three *Acropora* species across the GBR (mean AUC_TEST_ > 0.8; Figure 5a; Hosmer Jr. et al. 2013). SDM performance was subtly, yet significantly, different between species (Figure 5; formal statistical values provided in Table S6). Variation in predictive ability depended on which reef was excluded in cross‐validation analyses (reef‐level responses in Figure S4). Overall, models for *A. hyacinthus* and *A. kenti* had statistically similar performances, where larger AUC_TEST_ values indicated improved ability to differentiate occurrence from background points than the models for *A. spathulata* . We saw consistent and significant (*p* < 0.001) responses of SDM performance to spatial resolution within each species according to Kruskal‐Wallis tests for each metric ( *A. hyacinthus* : *H*_AUCTEST_(8) = 155.3, *H*_BIC_(8) = 688.8; *A. spathulata* : *H*_AUCTEST_(8) = 211.3, *H*_BIC_(8) = 478.5; *A. kenti* : *H*_AUCTEST_(8) = 171.1, *H*_BIC_(8) = 1021.9; *p* < 0.001). Dunn's non‐parametric post hoc tests for each species highlighted that SDMs with input variables at 15–60 m produced statistically similar models, which consistently outperformed 100 m and 120 m models (Figure 5; formal statistical values provided in Table S7). Bathymetry source had little impact on model performance at matching resolutions, except at 30 m when ACA outperformed DeepReef (Figure 5; formal statistical values provided in Table S7). Often, the predictive abilities of the ‘all variable’ models (*n* = 72 variables) were statistically similar to 15–60 m single resolution models at both AUC_TEST_ and BIC metrics (Figure 5; formal statistical values provided in Table S7).

### 3.4 Importance of Multiscale Topographic Variables for Regional Species Distribution Models

**FIGURE 6:**
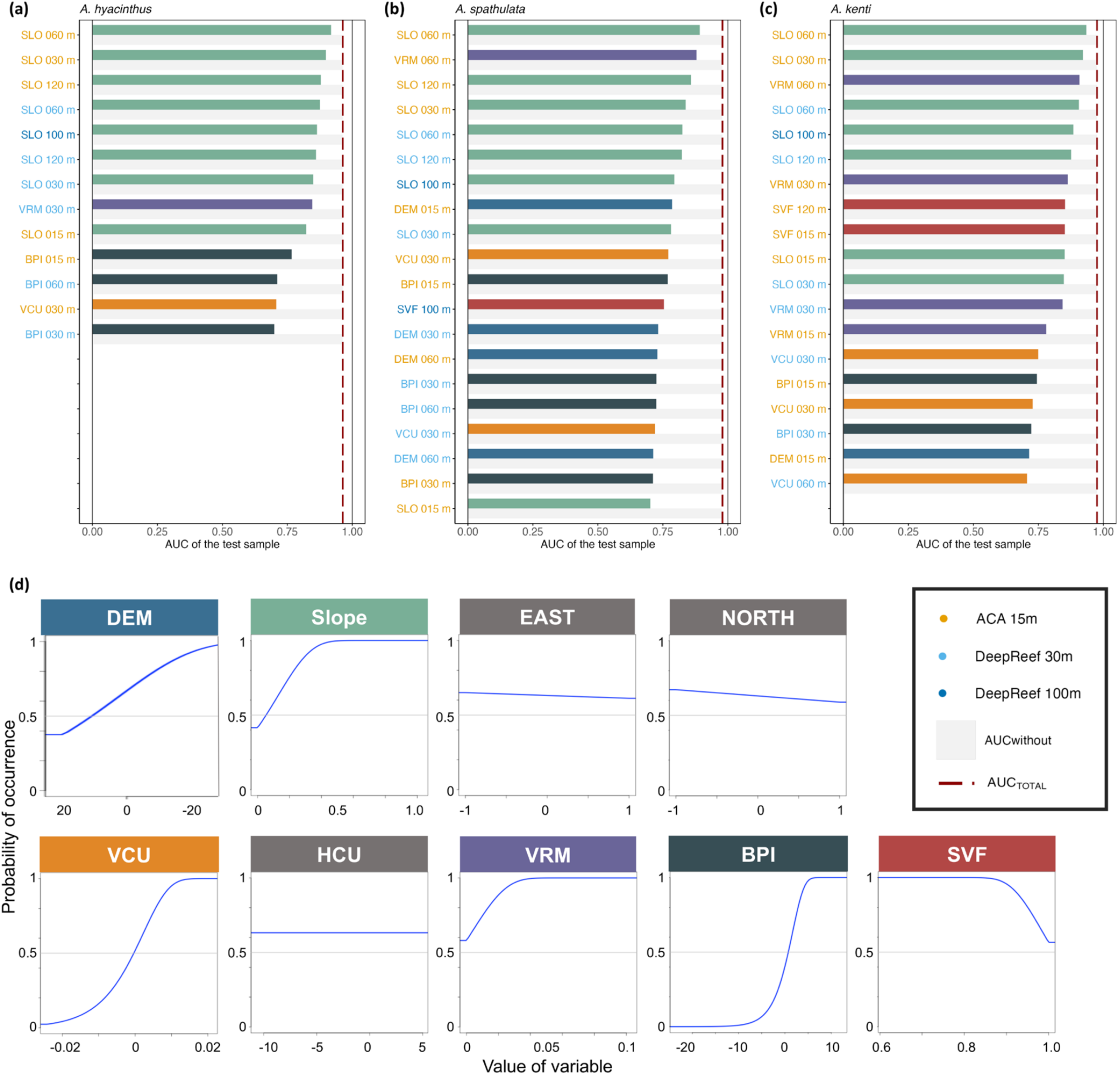
Jackknife assessments highlighting the relative importance of top predictor variables (i.e., those with AUC_ONLY_ > 0.7) used in MaxEnt distribution models for three *Acropora* coral species sampled across 23 reefs of the Great Barrier Reef: (a) *A. hyacinthus* , (b) *A. spathulata* , and (c) *A. kenti* . All 72 variables were used as predictors in the MaxEnt model for each species (full Jackknife results are in Figure S5). Relative importance is assessed by comparing the Area Under the receiver operating Curve (AUC) of the full model (AUC_TOTAL_; red dashed lines), with the AUC of a model run only with one target variable (AUC_ONLY_; coloured bars that match variable names in (d)) and with all variables except for the target (AUC_WITHOUT_; light grey bars). Variable abbreviations (detailed in Table 1) correspond to: DEM = digital elevation model, SLO = slope, EAST = eastness, NORTH = northness, VCU = vertical curvature, HCU = horizontal curvature, VRM = vector ruggedness measure, BPI = bathymetry position index and SVF = sky view factor. Y‐axis variable labels are coloured by the source of DEM: Allen Coral Atlas 15 m (ACA 15 m; orange), DeepReef 30 m (light blue), DeepReef 100 m (dark blue). Example response curves (d) highlight the individual effect of each variable type derived from ACA15 m on the predicted probability of *A. spathulata* occurrence (see Figures S6 and S7 for all marginal and individual response curves).

Jackknife analyses revealed the relative importance of the 72 variables used in SDM (top variables: Figure 6a–c; all variables shown in Figure S5). Here, we focus on the general trends of ‘top’ variables that, when used alone, produced models with ‘acceptable’ discrimination between occurrence and background points (i.e., AUC_ONLY_ > 0.7; Hosmer Jr. et al. 2013). Their interpretations were complemented with marginal response curves to visualise the variable's additive effect in MaxEnt models, given all other variables, along with the variable's independent effects on predictions (examples: Figure 6d; all variables: Figure S6). The species‐specific optimal resolutions for each variable are summarised in Table 1. The most important variable for predicting the three species' occurrences was SLOPE across all resolutions, notably at 30 m and 60 m. Considered with other variables, the predictive presence of species was highest on moderate slopes, with low presence on both completely flat and steep inclines. The terrain complexity variable of VRM was also important at 30 m or 60 m. When modelled with other variables, VRM at 30 m and 60 m highlight higher probabilities of *A. spathulata* and *A. kenti* on smoother broad‐scale terrain features (e.g., reef flats and straight lengths along reef slopes, rather than spur and groove systems), whilst *A. hyacinthus* appears to be less influenced by VRM. Other common top variables amongst species included VCU at 30 m and BPI at 15–60 m, where response curves were highly sensitive to resolution. Generally, higher presence was predicted on concave slopes (+VCU) and on local peaks and reef crests (+BPI). Whilst neither depth nor SVF were important for *A. hyacinthus* , both were top variables for *A. spathulata* (depth at 15–60 m; SVF at 100 m) and *A. kenti* (depth at 15 m; SVF at 15 m and 120 m). Interestingly, *A. spathulata* was slightly more associated with open areas (+SVF), whilst *A. kenti* was associated with more covered areas (‐SVF). Models using only aspect (EAST and NORTH) were no better than random, with slightly higher occurrence along western slopes. Additionally, HCU had no effect on predicted occurrence and was not used by MaxEnt for any species. As before, the source of bathymetric model generally had little impact on variable importance, though the top three variables for each species was based on ACA.

**FIGURE 7:**
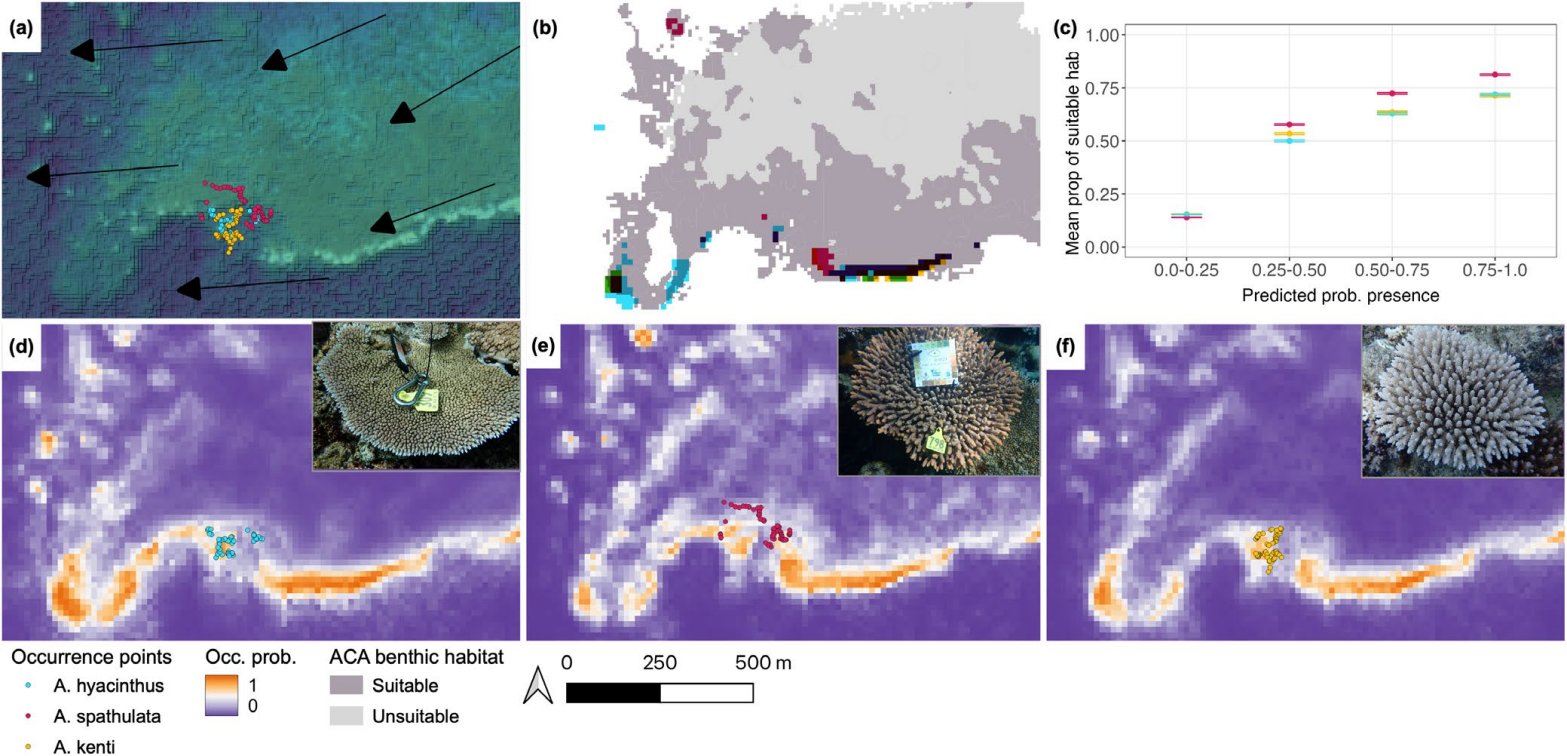
Visual assessment of MaxEnt prediction models compared with actual reef morphology and potential suitable habitat for three *Acropora* species, taking an example from the south‐west corner of Chicken Reef in the central Great Barrier Reef (indicated with a purple box in Figure 2). Reef morphology (a) is highlighted using satellite imagery superimposed on a bathymetry model, alongside mean current direction in summer (arrows), where sampled colonies of each species are indicated by coloured points. Areas of potential suitable habitat (b) are those identified as ‘coral/algae’ or ‘rock’ in the independent Allen Coral Atlas benthic map, where areas of high predicted occurrence (> 75% probability) according to MaxEnt models are superimposed for each species. Note that black areas indicate high predicted occurrence of multiple species. Panel (c) quantifies the relationship between mean ± standard error of potentially suitable habitat associated with the predicted probability of species' presences (grouped to 0%–25%, 25%–50%, 50%–75%, 75%–100%). Last, occurrence probability maps of (d) *A. hyacinthus* , (e) *A. spathulata* , and (f) *A. kenti* , determined using all 72 topographic variables in MaxEnt models, with photograph examples of sampled colonies for each species. Satellite imagery (a) is copyright to Planet Labs (2021, licenced CC BY‐SA‐NC 4.0).

Visual inspection of MaxEnt prediction maps, illustrated with an example from Chicken Reef (starred on the map of Figure 2), highlights higher occurrence probabilities coinciding with reef slopes and crests, based on satellite images (Figure 7a) and areas of potentially suitable habitat (‘coral/algae’ and ‘rock’ from the ACA benthic habitat map; Figure 7b). Across all modelled reefs, areas of high predicted presence (> 75%) were associated with 70%–80% suitable habitat, whilst regions of low predicted presence (0%–50%) contain < 20% suitable habitat (Figure 7c). Similar distributions were predicted for *A. hyacinthus* (Figure 7d) and *A. kenti* (Figure 7e), notably along reef crests and reef slopes, where *A. hyacinthus* was more likely to be found around coral reef outcrops. Meanwhile, *A. spathulata* was predicted to be more prevalent across reef flats and lagoons than on reef crests and slopes (Figure 7e).

## 4 Discussion

Topographic variables derived from open‐access bathymetry DEMs produced high‐performing, species‐specific distribution models for three *Acropora* coral species across the GBR. Using only eight derived variable types plus depth, we generated maps predicting coral distributions that resolved reef morphology and correlated high occurrence probabilities of the species with areas of potentially suitable habitat, as determined by independent remote sensed benthic maps (i.e., ACA benthic maps). Our findings corroborate studies highlighting the power of topographic variables for modelling deep‐water coral distributions (e.g., Bridge et al. 2012; Dolan et al. 2008; Tong et al. 2012) and for identifying shallow‐water coral recruitment habitats (e.g., Carlson et al. 2024; Radford et al. 2024). The novelty of the present study lies in its assessment of very high‐resolution bathymetric DEMs as fine as 15 m across an entire regional extent for shallow water coral distribution models. Here, we examine the importance of considering topographic variables derived from high‐resolution bathymetry models. We then discuss the consequences of using predictor variables at multiple spatial resolutions for mapping distributions of sessile marine organisms. Our proposed methods can be applied to map species‐specific marine organism distributions at other reef regions around the world, which can ultimately guide global coral reef research and conservation projects.

### 4.1 Bathymetric DEMs Share Similar Vertical Accuracies and Are Both Important for Shallow‐Water Coral Spatial Models

As DEM quality influences the relevance of derived topographic variables in ecological modelling, we first compared derived products of the open‐access and readily available bathymetry models of the ACA (Allen Coral Atlas 2022) and DeepReef (Beaman 2010), filling missing pixels of ACA with the DeepReef30 m DEM. We found that the vertical accuracies of DEMs, assessed to 10 m depth, were similar for DEMs from both sources at matching resolutions, where coarser pixel sizes had larger vertical errors compared to in situ measurements. We expect that approximately 1 m of the vertical inaccuracies can be attributed to technical factors of diving‐based sampling, as well as from tide corrections interpolated from the nearest tide stations. Regardless of these depth errors, we found the DEMs to be important for modelling distributions of three coral species in shallow waters.

Contrary to the current paradigm that finer resolutions result in higher SDM performance (e.g., Chauvier et al. 2022; Cushman and Landguth 2010; Moudrý et al. 2023), we find a slight improvement in SDM performance with 30 m and 60 m topographic variables. This suggests that the added computational time and expert knowledge required to process the 15 m variables from the ACA may not always be necessary. Simultaneously, the slightly lower performance of coarser variable models suggests that the DeepReef100 m model may not be resolved enough for modelling shallow water (< 10 m depth) coral, despite its success in modelling deep‐water reefs (Bridge et al. 2012). For research in the GBR, we can recommend prioritisation of the DeepReef30 m model, particularly when computational limitations are a concern or when the target species are known to occur deeper than 10 m. Given that the highest AUC values were consistently attributed to ACA derived variables (at 15, 30 and 60 m), when computational limitations are not a concern and the taxon of interest occurs predominantly in depths shallower than 10 m, the ACA would also be suitable. Furthermore, global availability of the ACA bathymetry maps makes them excellent resources to derive topographic variables for modelling shallow‐water corals in reefs outside of the GBR region, with the DeepReef also valuable across the GBR and Coral Sea.

Whilst the results presented here will be relevant for most coral species located on shallow reef (i.e., < 10 m depth; Lyons et al. 2024), the accuracy and importance of the bathymetry products for modelling species in waters deeper than 10 m cannot be specifically commented on here. We hypothesise that DeepReef bathymetry models are more accurate than ACA at > 10 m depth, as DeepReef uses more precise LiDAR and sonar data to complement satellite data (Beaman 2010, 2017, 2020). Furthermore, assessments of the ACA bathymetry maps indicate the maximum accuracy of DEMs is limited to 10–15 m depths, where the root mean squared error of DEMs doubles at 20 m depth (Li et al. 2021). Future work should compare the accuracies and importance of bathymetric DEM products, including from DeepReef and ACA, at depths > 10 m prior to use for deeper water species.

### 4.2 Multiscale Topographic Variables Are Important in Coral Spatial Models

Regardless of bathymetric source, the top variables for modelling all three *Acropora* species in shallow waters were those representing habitat complexity (i.e., Slope, VRM, VCU, BPI and SVF), where discussions on optimal species‐specific terrain attributes are provided in Section 4.3. These top variables are those that proxy for important physical processes that may impact coral settlement, growth, and survival (Pratchett et al. 2015) and are known to be important for marine habitat mapping (e.g., Brown et al. 2012; Dolan et al. 2008; Lecours, Brown, et al. 2016), including for coral SDMs (e.g., Bridge et al. 2012; Pygas et al. 2020; Tong et al. 2012). Indeed, slope affects benthic habitat composition and colonisation success by dictating sediment stability, water flow and current acceleration (Lecours, Dolan, et al. 2016; Wilson et al. 2007). The habitat complexity variables represent terrain structures that dictate coral exposure to biotic, abiotic and ecological factors, such as available surface for larval attachment and growth, access to nutrients and shelter that dictates predation risk, as well as variations in local environmental conditions (Asner et al. 2021; Chung et al. 2021; Whalan et al. 2015). For instance, VCU captures dominant landforms and geomorphic processes, such as ridges and channels, influencing patterns of sedimentation and currents (Lecours, Dolan, et al. 2016; Rowden et al. 2017; Wilson et al. 2007). VRM and BPI indicate crests and troughs impacting local habitat conditions, attachment sites, exposure, and thermal regimes (Misiuk et al. 2021; Pygas et al. 2020; Sappington et al. 2007; Tong et al. 2012; Wilson et al. 2007). Last, terrain openness, captured through SVF, impacts overhead light and wave action, with consequences for energy acquisition (e.g., photosynthetic rates and nutrients) and stress tolerance (e.g., wave energy and UV exposure) (Böhner and Antonić 2009). Whilst in the present study we selected only a few commonly used and easily calculated terrain variables, the importance of structural complexity on ecological, geomorphological and hydrodynamical processes has resulted in the development of a plethora of other terrain roughness metrics. Such variables that warrant investigation in future coral SDM studies include linear rugosity, skewness, effective and spectral slope, entropy and frontal solidity (e.g., Chung et al. 2021; Pygas et al. 2020; Smith 2014; Sous et al. 2024).

Variables representing aspect (EAST and NORTH) and HCU at the tested resolutions were seemingly irrelevant for modelling these species. It could be that these variables incorrectly capture environmental processes, either due to artefact errors in high‐resolution DEMs that are amplified in these primary terrain attributes (Lecours, Dolan, et al. 2016) or because the variables need to be at resolutions < 15 m or > 100 m to be relevant for modelling these species (Dolan et al. 2008; Pygas et al. 2020). For example, distribution modelling of the cold‐water coral, *Lophelia pertusa*, across an Irish seamount required aspect between 1.5–16 m, but not HCU at any resolution (Dolan et al. 2008), whilst HCU was instead important at 170 m across a Norwegian deep‐reef margin (Tong et al. 2016). Though fine‐resolution topographic variables < 10 m are excellent predictors in modelling coral habitats at local scales (Carlson et al. 2024; Radford et al. 2024), they remain unattainable across entire reef networks with current technologies (Bongaerts et al. 2021) and could therefore not be assessed in the present study. It is also possible that these topographic variables were not closely related to the environmental variables that we assume them to be environmental surrogates for (e.g., light availability), as we did not ground truth these relationships (e.g., we assume that aspect is a proxy for erosion and wave direction; Table 1). Here, we based our assumptions on previous research (see references in Introduction and Table S3), but it could be that the assumed relationship does not extend to our study area. Future research is needed to correlate derived topographic variables with empirically quantified environmental conditions.

Harnessing multi‐resolution variables can provide more nuanced information about processes impacting species distributions and lead to more accurate models for conservation management. The sensitivity of topographic variables to spatial scale is known to be highly complex (e.g., Pygas et al. 2020; Smith 2014; Sous et al. 2024), where we found in the current study that the optimal spatial resolution for coral SDMs depended on habitat characteristics, species biology and ecology, and topographic variable type. For instance, Jackknife analyses of the ‘all variable’ models revealed that the top‐performing variables were often at 30 m or 60 m resolutions. These mid‐resolutions could be advantageous due to smoothing over DEM acquisition noise amplified at finer resolutions (Pradervand et al. 2014). We show that depth and habitat complexity variables were most relevant at finer resolutions (i.e., 15 m and 30 m), whilst variables of general landform features, orientation, and openness were more relevant at mid resolutions (i.e., 60 m). By contrast, a similar study on deep‐sea coral distributions found that BPI, curvature and rugosity at 170 m were more relevant than 30 m or 90 m (Tong et al. 2012). The importance of finer resolution variables could stem from sample bias in depth‐restricted sampling. For instance, if a species is present deeper than what is sampled on a reef slope, the 15 m model might underpredict presence, which would otherwise be captured with coarser‐pixel DEM models. The 100 m and 120 m resolution variables were generally less important and associated with decreased model performance, likely due to loss of critical information related to terrain features and habitats such as reef outcrops.

As the relationships between variables at given spatial resolutions are strongly site‐ and species‐dependent, extrapolation to new areas is not advised, where unique sets of multiscale variables must be produced for each new area and species. For researchers interested in producing and using species‐specific marine SDMs built with DEM‐derived topographic variables in other regions of the world, we recommend comparing SDM prediction maps with high‐resolution remote sensed coral habitat and benthic classification maps. These can be accessed through open‐source resources including Millennium Coral Reef Mapping Project (Andréfouët and Bionaz 2021; Andréfouët and Riegl 2004), the Khaled bin Sultan Living Oceans Foundation (Carlton et al. 2021; Purkis et al. 2019), Reef Cover (Kennedy et al. 2021), and the Allen Coral Atlas (ACA; Allen Coral Atlas 2022).

### 4.3 Topographic Variable Importance Is Species‐Specific

Distinct coral prediction maps were produced for the three *Acropora* species, reflecting recorded habitat preferences (Veron 2000; Wallace et al. 2012) and aligning well with reef satellite images and generic coral habitat maps. *A. spathulata* was predicted across a broader range of geomorphic areas than the other species, reflecting its observed distribution across exposed and sheltered reef slopes, outer reef flats, and lagoons. Conversely, *A. kenti* and *A. hyacinthus* predictions were more limited to reef crests and upper reef slopes, consistent with their known habitat preferences for higher energy environments. The subtlety in distribution patterns between these species could be due to taxonomic closeness as members of the *Acropora* genus, with overlapping habitat preferences and distributions. To better understand coral relationships with topographic variables and potentially make generalisations about optimal variable resolutions, future work should compare more distantly related coral species with diverse morphologies and/or life history traits (such as investigating massive or dome‐shaped corals of the Poritidae or Pocilloporidae families) from contrasting habitats, particularly as the relevant spatial scale is expected to vary between and within species (Winship et al. 2020).

Species‐specific variable importance underlies the observed differences in predicted spatial distributions. The *A. hyacinthus* complex required fewer topographic variables than the other species, notably omitting depth and SVF, with some evidence from cross‐validations that these models were less transferable to novel locations (Elith et al. 2011). Rather than fine resolution topographic influences, this tabular species might be more affected by broader‐scale geographic, oceanographic or climatic variables, in line with its high sensitivity to storm damage, coral bleaching, and disease (Ortiz et al. 2021; Pratchett et al. 2015). By contrast, *A. spathulata* and *A. kenti* distributions were driven by topographic variables representing substrate composition, terrain variability, openness, and relative position, potentially due to their corymbose morphologies (Veron 2000) with broader bases for solid attachments in heterogeneous environments (Cresswell et al. 2020; Madin et al. 2012; Pratchett et al. 2015).

### 4.4 Harnessing a Multiscale Framework

As the sensitivity of ecological models to spatial scale varies with study species and topographic variable type, accurate mapping of species distributions for multi‐species conservation planning requires pragmatic methods to integrate species‐specific multiresolution variables (Pittman et al. 2021; Pygas et al. 2020). Here, we demonstrated that mid‐resolution variables (30 and 60 m) performed just as well as 15 m models, with performance dropping slightly at coarser resolutions (100 and 120 m), debunking the paradigm that the finest resolutions should be used whenever possible (Moudrý et al. 2023). Therefore, when faced with limitations in finances, time, and computational resources, slightly coarser resolution variables remain a practical option to obtain accurate SDMs. Whilst the present study focuses on species in the expansive coral reef network of the GBR, Australia, we propose a multiscale framework to facilitate selecting context‐specific optimal spatial resolutions for coral reef researchers around the globe, given that presence‐only or presence/absence data is available for the target species.

First, all potentially relevant variables must be generalised to multiple spatial resolutions. Here, we used a Gaussian pyramid to obtain DEMs at nested resolutions from which topographic variables were derived (following Guillaume et al. 2021; Kalbermatten et al. 2012; Leempoel et al. 2015). Whilst this resample‐calculate method (Misiuk et al. 2021) based on wavelet multi‐resolution decompositions is good when characterising specific benthic features or processes, it may not be optimal for multiscale implementations for all groups of variables (e.g., climatic, oceanographic, etc.). Other multiscale methods exist and should be selected based on study goals and data characteristics (see Misiuk et al. 2021; Smith 2014). Further investigation into different approaches could provide valuable knowledge for the integration of multiscale variables.

Once multiscale variables are produced, the second step involves integrating them into an ecological model that is best suited to the presence data available. Here, we integrated all variables into MaxEnt as predictors of coral distribution, as this SDM model based on presence‐only data can handle redundancy automatically in its machine learning algorithm (De Marco Júnior and Nóbrega 2018; Feng et al. 2019). Depending on the ecological model, collinearity amongst multiresolution variables may first need to be addressed. This can be done using simple correlation assessments, such as Spearman's pairwise correlations using |r_s_| ≥ 0.8 (Fischer et al. 2013) or variation inflation factors with VIF < 10 (Dormann et al. 2013). Alternatively, automatised variable selection procedures using either a step‐wise forward model (Blanchet et al. 2008) or an embedded covariate algorithm (Adde et al. 2023) aimed at optimising the predictive ability of SDMs could be used.

Here, we illustrate how topographic variables at multiple resolutions can produce species‐specific distribution models that are more nuanced than generic benthic habitat maps (e.g., the Millennium Coral Reef Mapping Project (Andréfouët and Bionaz 2021; Andréfouët and Riegl 2004); the Khaled bin Sultan Living Oceans Foundation (Carlton et al. 2021; Purkis et al. 2019); Reef Cover (Kennedy et al. 2021); the Allen Coral Atlas (Allen Coral Atlas 2022)). These species‐specific prediction maps can provide nuanced insights into the drivers of species distributions and act as null models against which to study the effects of climate change, where topography‐only models can be compared to models using geomorphic, oceanographic and climatic variables (e.g., ocean temperatures, seawater chemical composition, connectivity, and wave exposure; Bertelli et al. 2023; Pygas et al. 2020; Winship et al. 2020; Yuen et al. 2023).

This research represents a first step towards producing multiple multiscale species‐specific distribution prediction maps across an entire reef network relatively quickly using only open‐access bathymetric data, providing initial information to guide field expeditions for research and conservation planning alike. One such application might be to predict the distribution of bleached coral colonies of a species, given the presence of thermally sensitive individuals. The distribution maps obtained in this project are to be validated and used in upcoming RRAP research to guide the selection and prioritisation of sampling sites for continued coral restoration and adaptation research. Similar work can be done in other coral reef regions around the world by following the proposed framework and harnessing region‐specific bathymetry models to produce site‐specific species distribution maps to aid local coral reef restoration projects.

Beyond applications in SDM, this multiscale framework offers potential benefits for spatial population genomic analyses. For instance, integrating variables at appropriate spatial resolutions in seascape genomic (Riginos et al. 2016; Selmoni et al. 2020) approaches using genotype–environment association models may lead to more accurate hypotheses of candidate loci under selection (as shown in a terrestrial example of Guillaume, Leempoel, et al. 2024). The integration of multiscale variables into evolutionary ecology models will enable the development of robust and effective predictions for marine ecosystems research, ultimately contributing to improved conservation and management strategies.

## 5 Conclusions

Distribution models offer a practical and powerful way to identify priority areas for research and management intervention for multiple at‐risk species simultaneously, where the accuracy, and therefore relevance, of SDMs depends on the quality of the predictor variables. With increased access to predictor variables at ever‐finer spatial resolutions, the ecological relevance of variables must be assessed prior to their use in ecological modelling. For the three GBR open‐access, high resolution bathymetric models assessed here (ACA, DeepReef30 m and DeepReef100 m), we found that the finest resolution of 15 m was not always required for achieving high‐performing SDMs for the *Acropora* species investigated. Instead, optimal spatial resolutions fell between 15 m and 60 m depending on the species and variable type. By using the multiscale framework described here, we included variables quickly and efficiently at resolutions important for each specific species. This shows how a multiscale framework can be adapted to produce species‐specific distribution models for coral reefs around the world. Using multiscale variables as predictors in ecological models ultimately facilitates conservation decision making. By deepening our understanding of the environmental variables that drive spatial distributions we can produce more nuanced prediction maps. This enables conservation practitioners to better grasp the ecological dynamics at play across different spatial resolutions, ultimately enhancing our ability to predict marine species distributions and inform spatial planning.

## Author Contributions

**Annie S. Guillaume:** conceptualization (lead), data curation (equal), formal analysis (lead), funding acquisition (supporting), investigation (lead), methodology (lead), project administration (lead), validation (lead), visualization (lead), writing – original draft (lead), writing – review and editing (lead). **Renata Ferrari:** formal analysis (equal), methodology (equal), resources (supporting), validation (supporting), writing – review and editing (equal). **Oliver Selmoni:** formal analysis (equal), methodology (equal), writing – review and editing (equal). **Véronique J. L. Mocellin:** data curation (equal), formal analysis (supporting), methodology (supporting), writing – review and editing (equal). **Hugo Denis:** data curation (equal), formal analysis (supporting), methodology (supporting), writing – review and editing (equal). **Melissa Naugle:** data curation (equal), formal analysis (supporting), methodology (supporting), writing – review and editing (equal). **Emily Howells:** data curation (equal), funding acquisition (equal), methodology (supporting), resources (equal), supervision (supporting), writing – review and editing (equal). **Line K. Bay:** funding acquisition (equal), project administration (equal), resources (equal), supervision (equal), writing – review and editing (supporting). **Stéphane Joost:** conceptualization (equal), funding acquisition (equal), methodology (supporting), project administration (equal), resources (equal), supervision (lead), writing – review and editing (supporting).

## Conflicts of Interest

The authors declare no conflicts of interest.

## Supporting information


Appendix S1.

## Data Availability

All data and scripts that support the findings of this study are openly available on Dryad at https://doi.org/10.5061/dryad.wdbrv15vk (Guillaume, Ferrari, et al. 2024). Publicly available data can be found at the following repositories: Coordinates (X, Y, depth) records maintained by AIMS (https://doi.org/10.25845/8DJ4‐0V58). Multiscale DEMs and topographic variables from the AIMS archive (https://doi.org/10.25845/ggyv‐pq15). Allen Coral Atlas Digital Elevation Models (https://doi.org/10.5281/zenodo.3833242). DeepReef30m Digital Elevation Model (https://doi.org/10.4225/25/5a207b36022d2). DeepReef100m Digital Elevation Model (www.deepreef.org/2010/07/06/gbr‐bathy/).

## References

[ece371292-bib-0001] Abrego, D. , E. J. Howells , S. D. A. Smith , et al. 2021. “Factors Limiting the Range Extension of Corals Into High‐Latitude Reef Regions.” Diversity 13: 1–16. 10.3390/d13120632.

[ece371292-bib-0002] Adde, A. , P. L. Rey , F. Fopp , et al. 2023. “Too Many Candidates: Embedded Covariate Selection Procedure for Species Distribution Modelling With the Covsel R Package.” Ecological Informatics 75: 102080. 10.1016/j.ecoinf.2023.102080.

[ece371292-bib-0003] Ahmadi, M. , M.‐R. Hemami , M. Kaboli , and F. Shabani . 2023. “MaxEnt Brings Comparable Results When the Input Data Are Being Completed; Model Parameterization of Four Species Distribution Models.” Ecology and Evolution 13, no. 2: e9827. 10.1002/ece3.9827. 36820245 PMC9937880

[ece371292-bib-0004] Allen Coral Atlas . 2022. “Imagery, Maps and Monitoring of the World's Tropical Coral Reefs [WWW Document].” 10.5281/zenodo.3833242.

[ece371292-bib-0005] Anderson, C. D. , B. k. Epperson , M.‐J. Fortin , et al. 2010. “Considering Spatial and Temporal Scale in Landscape‐Genetic Studies of Gene Flow.” Molecular Ecology 19: 3565–3575. 10.1111/j.1365-294X.2010.04757.x. 20723051

[ece371292-bib-0006] Andréfouët, S. , and O. Bionaz . 2021. “Lessons From a Global Remote Sensing Mapping Project. A Review of the Impact of the Millennium Coral Reef Mapping Project for Science and Management.” Science of the Total Environment 776: 145987. 10.1016/j.scitotenv.2021.145987.

[ece371292-bib-0007] Andréfouët, S. , and B. Riegl . 2004. “Remote Sensing: A Key Tool for Interdisciplinary Assessment of Coral Reef Processes.” Coral Reefs 23: 1–4. 10.1007/s00338-003-0360-z.

[ece371292-bib-0008] Asner, G. P. , N. R. Vaughn , S. A. Foo , J. Heckler , and R. E. Martin . 2021. “Abiotic and Human Drivers of Reef Habitat Complexity Throughout the Main Hawaiian Islands.” Frontiers in Marine Science 8: 1–13. 10.3389/fmars.2021.631842.35685121

[ece371292-bib-0009] Bakker, A. C. , A. C. R. Gleason , A. C. Dempsey , H. E. Fox , R. H. Green , and S. J. Purkis . 2024. “Remotely Sensed Habitat Diversity Predicts Species Diversity on Coral Reefs.” Remote Sensing of Environment 302: 113990. 10.1016/j.rse.2024.113990.

[ece371292-bib-0010] Beaman, R. J. 2010. Project 3D‐GBR: A high‐resolution depth model for the Great Barrier Reef and Coral Sea. Marine and Tropical Sciences Research Facility (MTSRF) Project 2.5i.1a Final Report. 10.4225/25/5a207b36022d2.

[ece371292-bib-0011] Beaman, R. J. 2017. “High‐Resolution Depth Model for the Great Barrier Reef – 30 m Dataset.” 10.4225/25/5a207b36022d2.

[ece371292-bib-0012] Beaman, R. J. 2020. “High‐Resolution Depth Model for the Great Barrier Reef and Coral Sea – 100 m.” 10.26186/5e2f8bb629d07.

[ece371292-bib-0013] Bertelli, C. M. , W. G. Bennett , H. Karunarathna , D. E. Reeve , R. K. F. Unsworth , and J. C. Bull . 2023. “High‐Resolution Wave Data for Improving Marine Habitat Suitability Models.” Frontiers in Marine Science 9: 1–14. 10.3389/fmars.2022.1004829.

[ece371292-bib-0014] Blanchet, F. G. , P. Legendre , and D. Borcard . 2008. “Forward Selection of Explanatory Variables.” Ecology 89: 2623–2632. 10.1890/07-0986.1. 18831183

[ece371292-bib-0015] Böhner, J. , and O. Antonić . 2009. “Land‐Surface Parameters Specific to Topo‐Climatology.” In Developments in Soil Science, edited by T. Hengl and H. I. Reuter , 195–226. Elsevier B.V. 10.1016/S0166-2481(08)00008-1.

[ece371292-bib-0016] Böhner, J. , R. Köthe , O. Conrad , J. Gross , A. Ringeler , and T. Selige . 2002. Soil Regionalisation by Means of Terrain Analysis and Process Parameterisation, Soil Classification 2001. European Soil Bureau, Research Report No. 7, EUR 20398 EN.

[ece371292-bib-0017] Bongaerts, P. , C. E. Dubé , K. E. Prata , J. C. Gijsbers , M. Achlatis , and A. Hernandez‐Agreda . 2021. “Reefscape Genomics: Leveraging Advances in 3D Imaging to Assess Fine‐Scale Patterns of Genomic Variation on Coral Reefs.” Frontiers in Marine Science 8: 1–9. 10.3389/fmars.2021.638979.35685121

[ece371292-bib-0018] Bradley, A. P. 1997. “The Use of the Area Under the ROC Curve in the Evaluation of Machine Learning Algorithms.” Pattern Recognition 30: 1145–1159. 10.1016/S0031-3203(96)00142-2.

[ece371292-bib-0019] Brenning, A. , D. Bangs , and M. Becker . 2022. “RSAGA: SAGA Geoprocessing and Terrain Analysis.” R package version 1.4.0.

[ece371292-bib-0020] Bridge, T. , R. Beaman , T. Done , and J. Webster . 2012. “Predicting the Location and Spatial Extent of Submerged Coral Reef Habitat in the Great Barrier Reef World Heritage Area, Australia.” PLoS One 7, no. 10: e48203. 10.1371/journal.pone.0048203. 23118952 PMC3484119

[ece371292-bib-0021] Bridge, T. C. L. , P. F. Cowman , A. M. Quattrini , et al. 2023. “A Tenuis Relationship: Traditional Taxonomy Obscures Systematics and Biogeography of the ‘*Acropora tenuis*’ (Scleractinia: Acroporidae) Species Complex.” Zoological Journal of the Linnean Society 202, no. 1: zlad062. 10.1093/zoolinnean/zlad062.

[ece371292-bib-0022] Brown, C. J. , J. A. Sameoto , and S. J. Smith . 2012. “Multiple Methods, Maps, and Management Applications: Purpose Made Seafloor Maps in Support of Ocean Management.” Journal of Sea Research 72: 1–13. 10.1016/j.seares.2012.04.009.

[ece371292-bib-0023] Carlson, R. R. , L. B. Crowder , R. E. Martin , and G. P. Asner . 2024. “The Effect of Reef Morphology on Coral Recruitment at Multiple Spatial Scales.” Proceedings of the National Academy of Sciences 121: 2017. 10.1073/pnas.2311661121. PMC10823213 38190515

[ece371292-bib-0024] Carlton, R. , A. Dempsey , L. Thompson , et al. 2021. Global Reef Expedition Final Report . Annapolis, MD.

[ece371292-bib-0025] Chauvier, Y. , P. Descombes , M. Guéguen , L. Boulangeat , W. Thuiller , and N. E. Zimmermann . 2022. “Resolution in Species Distribution Models Shapes Spatial Patterns of Plant Multifaceted Diversity.” Ecography 2022, no. 10: e05973. 10.1111/ecog.05973.

[ece371292-bib-0026] Chung, D. , N. Hutchins , M. P. Schultz , and K. A. Flack . 2021. “Predicting the Drag of Rough Surfaces.” Annual Review of Fluid Mechanics 53: 439–471. 10.1146/annurev-fluid-062520-115127.

[ece371292-bib-0027] Colberg, F. , G. B. Brassington , P. Sandery , P. Sakov , and S. Aijaz . 2020. “High and Medium Resolution Ocean Models for the Great Barrier Reef.” Ocean Modelling 145: 101507. 10.1016/j.ocemod.2019.101507.

[ece371292-bib-0028] Connor, T. , V. Hull , A. Viña , et al. 2018. “Effects of Grain Size and Niche Breadth on Species Distribution Modeling.” Ecography 41: 1270–1282. 10.1111/ecog.03416.

[ece371292-bib-0029] Conrad, O. , B. Bechtel , M. Bock , et al. 2015. “System for Automated Geoscientific Analyses (SAGA) v. 2.1.4.” Geoscientific Model Development 8: 1991–2007. 10.5194/gmd-8-1991-2015.

[ece371292-bib-0030] Cooke, I. , H. Ying , S. Forêt , et al. 2020. “Genomic Signatures in the Coral Holobiont Reveal Host Adaptations Driven by Holocene Climate Change and Reef Specific Symbionts.” Science Advances 6: eabc6318. 10.1126/sciadv.abc6318. PMC7695477 33246955

[ece371292-bib-0031] Cornwall, C. E. , S. Comeau , N. A. Kornder , et al. 2021. “Global Declines in Coral Reef Calcium Carbonate Production Under Ocean Acidification and Warming.” Proceedings of the National Academy of Sciences of the United States of America 118, no. 21: e2015265118. 10.1073/pnas.2015265118. 33972407 PMC8166140

[ece371292-bib-0032] Cresswell, A. K. , M. Orr , M. Renton , et al. 2020. “Structure‐From‐Motion Reveals Coral Growth Is Influenced by Colony Size and Wave Energy on the Reef Slope at Ningaloo Reef, Western Australia.” Journal of Experimental Marine Biology and Ecology 530‐531: 151438. 10.1016/j.jembe.2020.151438.

[ece371292-bib-0033] Cushman, S. A. , and E. L. Landguth . 2010. “Scale Dependent Inference in Landscape Genetics.” Landscape Ecology 25: 967–979. 10.1007/s10980-010-9467-0. 20618896

[ece371292-bib-0034] De Marco Júnior, P. , and C. C. Nóbrega . 2018. “Evaluating Collinearity Effects on Species Distribution Models: An Approach Based on Virtual Species Simulation.” PLoS One 13, no. 9: e0202403. 10.1371/journal.pone.0202403. 30204749 PMC6133275

[ece371292-bib-0035] Deng, Y. , J. P. Wilson , and B. O. Bauer . 2007. “DEM Resolution Dependencies of Terrain Attributes Across a Landscape.” International Journal of Geographical Information Science 21: 187–213. 10.1080/13658810600894364.

[ece371292-bib-0036] Denis, H. , L. K. Bay , V. J. L. Mocellin , et al. 2024. “Thermal Tolerance Traits of Individual Corals Are Widely Distributed Across the Great Barrier Reef.” Proceedings of the Royal Society B: Biological Sciences 291: 202420240587. 10.1098/rspb.2024.0587. PMC11463214 39257340

[ece371292-bib-0037] Devlin, M. J. , E. T. da Silva , C. Petus , et al. 2013. “Combining In‐Situ Water Quality and Remotely Sensed Data Across Spatial and Temporal Scales to Measure Variability in Wet Season Chlorophyll‐a: Great Barrier Reef Lagoon (Queensland, Australia).” Ecological Processes 2: 31. 10.1186/2192-1709-2-31.

[ece371292-bib-0038] Dolan, M. F. J. , A. J. Grehan , J. C. Guinan , and C. Brown . 2008. “Modelling the Local Distribution of Cold‐Water Corals in Relation to Bathymetric Variables: Adding Spatial Context to Deep‐Sea Video Data.” Deep Sea Research Part I: Oceanographic Research Papers 55: 1564–1579. 10.1016/j.dsr.2008.06.010.

[ece371292-bib-0039] Donovan, M. K. , D. E. Burkepile , C. Kratochwill , et al. 2021. “Local Conditions Magnify Coral Loss After Marine Heatwaves.” Science 372: 977–980. 10.1126/science.abd9464. 34045353

[ece371292-bib-0040] Dormann, C. F. , J. Elith , S. Bacher , et al. 2013. “Collinearity: A Review of Methods to Deal With It and a Simulation Study Evaluating Their Performance.” Ecography Cop. 36: 27–46. 10.1111/j.1600-0587.2012.07348.x.

[ece371292-bib-0041] Duce, S. , A. Vila‐Concejo , S. M. Hamylton , J. M. Webster , E. Bruce , and R. J. Beaman . 2016. “A Morphometric Assessment and Classification of Coral Reef Spur and Groove Morphology.” Geomorphology 265: 68–83. 10.1016/j.geomorph.2016.04.018.

[ece371292-bib-0042] Elith, J. , and J. R. Leathwick . 2009. “Species Distribution Models: Ecological Explanation and Prediction Across Space and Time.” Annual Review of Ecology, Evolution, and Systematics 40: 677–697. 10.1146/annurev.ecolsys.110308.120159.

[ece371292-bib-0043] Elith, J. , S. J. Phillips , T. Hastie , M. Dudík , Y. E. Chee , and C. J. Yates . 2011. “A Statistical Explanation of MaxEnt for Ecologists.” Diversity and Distributions 17: 43–57. 10.1111/j.1472-4642.2010.00725.x.

[ece371292-bib-0044] Fator, M. , and N. Zomrawi . 2015. “Etrex Garmin GPS Receiver Accuracy Testing.” International Journal on Recent and Innovation Trends in Computing and Communication 3: 2772–2774. 10.17762/ijritcc2321-8169.150558.

[ece371292-bib-0045] Feng, X. , D. S. Park , Y. Liang , R. Pandey , and M. Papeş . 2019. “Collinearity in Ecological Niche Modeling: Confusions and Challenges.” Ecology and Evolution 9: 10365–10376. 10.1002/ece3.5555. 31624555 PMC6787792

[ece371292-bib-0046] Ferrari, R. , H. A. Malcolm , M. Byrne , et al. 2018. “Habitat Structural Complexity Metrics Improve Predictions of Fish Abundance and Distribution.” Ecography 41: 1077–1091. 10.1111/ecog.02580.

[ece371292-bib-0049] Fischer, M. C. , C. Rellstab , A. Tedder , et al. 2013. “Population Genomic Footprints of Selection and Associations With Climate in Natural Populations of Arabidopsis Halleri From the Alps.” Molecular Ecology 22: 5594–5607. 10.1111/mec.12521. 24102711 PMC4274019

[ece371292-bib-0050] Goetze, J. S. , S. Wilson , B. Radford , et al. 2021. “Increased Connectivity and Depth Improve the Effectiveness of Marine Reserves.” Global Change Biology 27: 3432–3447. 10.1111/gcb.15635. 34015863 PMC8360116

[ece371292-bib-0051] Graham, N. A. J. , and K. L. Nash . 2013. “The Importance of Structural Complexity in Coral Reef Ecosystems.” Coral Reefs 32: 315–326. 10.1007/s00338-012-0984-y.

[ece371292-bib-0052] Guest, J. R. , A. H. Baird , J. A. Maynard , et al. 2012. “Contrasting Patterns of Coral Bleaching Susceptibility in 2010 Suggest an Adaptive Response to Thermal Stress.” PLoS One 7: 1–8. 10.1371/journal.pone.0033353. PMC3302856 22428027

[ece371292-bib-0053] Guillaume, A. S. , R. Ferrari , O. Selmoni , et al. 2024. “Derived Variables and Coordinates to Assess the Ecological Relevance of Multiscale Bathymetry for Coral Species Distribution Modelling Across the Great Barrier Reef [Dataset].” 10.5061/dryad.wdbrv15vk.Dryad.

[ece371292-bib-0054] Guillaume, A. S. , K. Leempoel , E. Rochat , et al. 2021. “Multiscale Very High Resolution Topographic Models in Alpine Ecology: Pros and Cons of Airborne LiDAR and Drone‐Based Stereo‐Photogrammetry Technologies.” Remote Sensing 13: 1588. 10.3390/rs13081588.

[ece371292-bib-0055] Guillaume, A. S. , K. Leempoel , A. Rogivue , F. Gugerli , C. Parisod , and S. Joost . 2024. “Integrating Very High Resolution Environmental Proxies in Genotype–Environment Association Studies.” Ecological Applications 17: e13737. 10.1111/eva.13737. PMC11212006 38948540

[ece371292-bib-0056] Guisan, A. , C. H. Graham , J. Elith , et al. 2007. “Sensitivity of Predictive Species Distribution Models to Change in Grain Size.” Diversity and Distributions 13: 332–340. 10.1111/j.1472-4642.2007.00342.x.

[ece371292-bib-0057] Hamylton, S. M. , J. D. Hedley , and R. J. Beaman . 2015. “Derivation of High‐Resolution Bathymetry From Multispectral Satellite Imagery: A Comparison of Empirical and Optimisation Methods Through Geographical Error Analysis.” Remote Sensing 7: 16257–16273. 10.3390/rs71215829.

[ece371292-bib-0058] Häntzschel, J. , V. Goldberg , and C. Bernhofer . 2007. “GIS‐Based Regionalisation of Radiation, Temperature and Coupling Measures in Complex Terrain for Low Mountain Ranges.” Meteorological Applications 12: 33–42. 10.1017/S1350482705001489.

[ece371292-bib-0059] Harris, P. T. , and E. K. Baker . 2012. Seafloor Geomorphology as Benthic Habitat: GeoHab Atlas of Seafloor Geomorphic Features and Benthic Habitats. Elsevier. 10.1016/C2010-0-67010-6.

[ece371292-bib-0061] Hazraty‐Kari, S. , P. Tavakoli‐Kolour , S. Kitanobo , et al. 2022. “Adaptations by the Coral *Acropora tenuis* Confer Resilience to Future Thermal Stress.” Communications Biology 5: 1–10. 10.1038/s42003-022-04309-5. 36517561 PMC9751277

[ece371292-bib-0062] Höhle, J. , and M. Höhle . 2009. “Accuracy Assessment of Digital Elevation Models by Means of Robust Statistical Methods.” ISPRS Journal of Photogrammetry and Remote Sensing 64: 398–406. 10.1016/j.isprsjprs.2009.02.003.

[ece371292-bib-0063] Höhle, J. , and M. Potuckova . 2006. “The EuroSDR Test: Checking and Improving of Digital Terrain Models.” In European Spatial Data Research, 9–141. Gopher.

[ece371292-bib-0064] Hosmer, D. W., Jr. , S. Lemeshow , and R. X. Sturdivant . 2013. Applied Logistic Regression. 3rd ed. John Wiley & Sons, Inc. 10.1002/9781118548387.

[ece371292-bib-0065] Hughes, T. P. , J. T. Kerry , M. Álvarez‐Noriega , et al. 2017. “Global Warming and Recurrent Mass Bleaching of Corals.” Nature 543: 373–377. 10.1038/nature21707. 28300113

[ece371292-bib-0066] Ilich, A. R. , B. Misiuk , V. Lecours , and S. A. Murawski . 2023. “MultiscaleDTM: An Open‐Source R Package for Multiscale Geomorphometric Analysis.” Transactions in GIS 27: 929–1286. 10.1111/tgis.13067.

[ece371292-bib-0067] International Hydrographic Organization . 2020. “Standards for Hydrographic Surveys: S‐44 Edition 6.1.0.”

[ece371292-bib-0068] Kalbermatten, M. , D. Van De Ville , P. Turberg , D. Tuia , and S. Joost . 2012. “Multiscale Analysis of Geomorphological and Geological Features in High Resolution Digital Elevation Models Using the Wavelet Transforms.” Geomorphology 138: 352–363. 10.1016/j.geomorph.2011.09.023.

[ece371292-bib-0069] Kennedy, E. V. , C. M. Roelfsema , M. B. Lyons , et al. 2021. “Reef Cover, a Coral Reef Classification for Global Habitat Mapping From Remote Sensing.” Scientific Data 8: 1–20. 10.1038/s41597-021-00958-z. 34341357 PMC8329285

[ece371292-bib-0070] Kool, J. T. , A. Moilanen , and E. A. Treml . 2013. “Population Connectivity: Recent Advances and New Perspectives.” Landscape Ecology 28: 165–185. 10.1007/s10980-012-9819-z.

[ece371292-bib-0071] Ladner, J. T. , and S. R. Palumbi . 2012. “Extensive Sympatry, Cryptic Diversity and Introgression Throughout the Geographic Distribution of Two Coral Species Complexes.” Molecular Ecology 21: 2224–2238. 10.1111/j.1365-294X.2012.05528.x. 22439812

[ece371292-bib-0072] Lecours, V. , C. J. Brown , R. Devillers , V. L. Lucieer , and E. N. Edinger . 2016. “Comparing Selections of Environmental Variables for Ecological Studies: A Focus on Terrain Attributes.” PLoS One 11: 1–18. 10.1371/journal.pone.0167128. PMC5176161 28002453

[ece371292-bib-0073] Lecours, V. , R. Devillers , E. N. Edinger , C. J. Brown , and V. L. Lucieer . 2017. “Influence of Artefacts in Marine Digital Terrain Models on Habitat Maps and Species Distribution Models: A Multiscale Assessment.” Remote Sensing in Ecology and Conservation 3: 232–246. 10.1002/rse2.49.

[ece371292-bib-0074] Lecours, V. , R. Devillers , D. C. Schneider , V. L. Lucieer , C. J. Brown , and E. N. Edinger . 2015. “Spatial Scale and Geographic Context in Benthic Habitat Mapping: Review and Future Directions.” Marine Ecology Progress Series 535: 259–284. 10.3354/meps11378.

[ece371292-bib-0075] Lecours, V. , R. Devillers , A. E. Simms , V. L. Lucieer , and C. J. Brown . 2017. “Towards a Framework for Terrain Attribute Selection in Environmental Studies.” Environmental Modelling & Software 89: 19–30. 10.1016/j.envsoft.2016.11.027.

[ece371292-bib-0076] Lecours, V. , M. F. J. Dolan , A. Micallef , and V. L. Lucieer . 2016. “A Review of Marine Geomorphometry, the Quantitative Study of the Seafloor.” Hydrology and Earth System Sciences 20: 3207–3244. 10.5194/hess-20-3207-2016.

[ece371292-bib-0077] Leempoel, K. , C. Parisod , C. Geiser , L. Daprà , P. Vittoz , and S. Joost . 2015. “Very High‐Resolution Digital Elevation Models: Are Multi‐Scale Derived Variables Ecologically Relevant?” Methods in Ecology and Evolution 6: 1373–1383. 10.1111/2041-210X.12427.

[ece371292-bib-0078] Lepczyk, C. A. , L. M. Wedding , G. P. Asner , et al. 2021. “Advancing Landscape and Seascape Ecology From a 2D to a 3D Science.” Bioscience 71: 1–13. 10.1093/biosci/biab001.

[ece371292-bib-0079] Li, J. , D. E. Knapp , M. Lyons , et al. 2021. “Automated Global Shallow Water Bathymetry Mapping Using Google Earth Engine.” Remote Sensing 13, no. 8: 1469. 10.3390/rs13081469.

[ece371292-bib-0080] Li, J. , D. E. Knapp , S. R. Schill , et al. 2019. “Adaptive Bathymetry Estimation for Shallow Coastal Waters Using Planet Dove Satellites.” Remote Sensing of Environment 232: 111302. 10.1016/j.rse.2019.111302.

[ece371292-bib-0081] Li, J. , S. R. Schill , D. E. Knapp , and G. P. Asner . 2019. “Object‐Based Mapping of Coral Reef Habitats Using Planet Dove Satellites.” Remote Sensing 11: 1445. 10.3390/rs11121445.

[ece371292-bib-0082] Lukoschek, V. , C. Riginos , and M. J. H. Van Oppen . 2016. “Congruent Patterns of Connectivity Can Inform Management for Broadcast Spawning Corals on the Great Barrier Reef.” Molecular Ecology 25: 3065–3080. 10.1111/mec.13649. 27085309

[ece371292-bib-0083] Lundblad, E. R. , D. J. Wright , J. Miller , et al. 2006. “A Benthic Terrain Classification Scheme for American Samoa.” Marine Geodesy 29: 89–111. 10.1080/01490410600738021.

[ece371292-bib-0084] Lyons, M. B. , N. J. Murray , E. V. Kennedy , et al. 2024. “New Global Area Estimates for Coral Reefs From High‐Resolution Mapping.” Cell Reports Sustainability 1: 100015. 10.1016/j.crsus.2024.100015.

[ece371292-bib-0085] Lyons, M. B. , C. M. Roelfsema , E. V. Kennedy , et al. 2020. “Mapping the World's Coral Reefs Using a Global Multiscale Earth Observation Framework.” Remote Sensing in Ecology and Conservation 6: 557–568. 10.1002/rse2.157.

[ece371292-bib-0086] Madin, J. S. , T. P. Hughes , and S. R. Connolly . 2012. “Calcification, Storm Damage and Population Resilience of Tabular Corals Under Climate Change.” PLoS One 7: 1–10. 10.1371/journal.pone.0046637. PMC3464260 23056379

[ece371292-bib-0136] Marlowe, C. , K. Hyder , M. D. J. Sayer , et al. 2021. “Divers as Citizen Scientists: Response Time, Accuracy and Precision of Water Temperature Measurement Using Dive Computers.” Frontiers in Marine Science 8. 10.3389/fmars.2021.617691.

[ece371292-bib-0087] McArthur, M. A. , B. P. Brooke , R. Przeslawski , et al. 2010. “On the Use of Abiotic Surrogates to Describe Marine Benthic Biodiversity.” Estuarine, Coastal and Shelf Science 88: 21–32. 10.1016/j.ecss.2010.03.003.

[ece371292-bib-0088] McClanahan, T. R. , and M. K. Azali . 2021. “Environmental Variability and Threshold Model's Predictions for Coral Reefs.” Frontiers in Marine Science 8: 1–16. 10.3389/fmars.2021.7781212021.35685121

[ece371292-bib-0089] McCullagh, P. , and J. A. Nelder . 1989. Generalized Linear Model. Chapman and Hall. 10.1007/978-1-4899-3242-6.

[ece371292-bib-0090] Merow, C. , M. J. Smith , and J. A. Silander . 2013. “A Practical Guide to MaxEnt for Modeling Species' Distributions: What It Does, and Why Inputs and Settings Matter.” Ecography Cop. 36: 1058–1069. 10.1111/j.1600-0587.2013.07872.x.

[ece371292-bib-0091] Misiuk, B. , V. Lecours , M. F. J. Dolan , and K. Robert . 2021. “Evaluating the Suitability of Multi‐Scale Terrain Attribute Calculation Approaches for Seabed Mapping Applications.” Marine Geodesy 44: 327–385. 10.1080/01490419.2021.1925789.

[ece371292-bib-0092] Montesano, P. M. , C. Neigh , G. Sun , L. Duncanson , J. Van Den Hoek , and K. J. Ranson . 2017. “The Use of Sun Elevation Angle for Stereogrammetric Boreal Forest Height in Open Canopies.” Remote Sensing of Environment 196: 76–88. 10.1016/j.rse.2017.04.024. 32848282 PMC7446955

[ece371292-bib-0093] Moore, I. D. , R. B. Grayson , and A. R. Ladson . 1991. “Digital Terrain Modelling: A Review of Hydrological, Geomorphological, and Biological Applications.” Hydrological Processes 5: 3–30. 10.1002/hyp.3360050103.

[ece371292-bib-0094] Morais, J. , S. B. Tebbett , R. A. Morais , and D. R. Bellwood . 2023. “Natural Recovery of Corals After Severe Disturbance.” Ecology Letters 27: e14332. 10.1111/ele.14332. 37850584

[ece371292-bib-0095] Moudrý, V. , P. Keil , A. F. Cord , et al. 2023. “Scale Mismatches Between Predictor and Response Variables in Species Distribution Modelling: A Review of Practices for Appropriate Grain Selection.” Progress in Physical Geography 47: 467–482. 10.1177/03091333231156362.

[ece371292-bib-0096] Naugle, M. S. , H. Denis , V. J. L. Mocellin , et al. 2024. “Heat Tolerance Varies Considerably Within a Reef‐Building Coral Species on the Great Barrier Reef.” Communications Earth & Environment 5, no. 1: 525. 10.1038/s43247-024-01649-4.

[ece371292-bib-0097] Ortiz, J. C. , R. J. Pears , R. Beeden , et al. 2021. “Important Ecosystem Function, Low Redundancy and High Vulnerability: The Trifecta Argument for Protecting the Great Barrier Reef's Tabular Acropora.” Conservation Letters 14: 1–18. 10.1111/conl.12817.

[ece371292-bib-0098] Otto, S. P. 2018. “Adaptation, Speciation and Extinction in the Anthropocene.” Proceedings of the Royal Society B: Biological Sciences 285: 20182047. 10.1098/rspb.2018.2047. PMC6253383 30429309

[ece371292-bib-0099] Phillips, S. J. , R. P. Anderson , M. Dudík , R. E. Schapire , and M. E. Blair . 2017. “Opening the Black Box: An Open‐Source Release of Maxent.” Ecography 40: 887–893. 10.1111/ecog.03049.

[ece371292-bib-0100] Phillips, S. J. , R. P. Anderson , and R. E. Schapire . 2006. “Maximum Entropy Modeling of Species Geographic Distributions.” Ecological Modelling 190, no. 3‐4: 231–259. 10.1016/j.ecolmodel.2005.03.026.

[ece371292-bib-0101] Phillips, S. J. , and M. Dudík . 2008. “Modeling of Species Distributions With Maxent: New Extensions and a Comprehensive Evaluation.” Ecography 31: 161–175. 10.1111/j.2007.0906-7590.05203.x.

[ece371292-bib-0102] Phillips, S. J. , M. Dudík , J. Elith , et al. 2009. “Sample Selection Bias and Presence‐Only Distribution Models: Implications for Background and Pseudo‐Absence Data.” Ecological Applications 19: 181–197. 10.1890/07-2153.1. 19323182

[ece371292-bib-0103] Pittman, S. , K. Yates , P. Bouchet , et al. 2021. “Seascape Ecology: Identifying Research Priorities for an Emerging Ocean Sustainability Science.” Marine Ecology Progress Series 663: 1–29. 10.3354/meps13661.

[ece371292-bib-0104] Pradervand, J.‐N. , A. Dubuis , L. Pellissier , A. Guisan , and C. Randin . 2014. “Very High Resolution Environmental Predictors in Species Distribution Models: Moving Beyond Topography?” Progress in Physical Geography: Earth and Environment 38: 79–96. 10.1177/0309133313512667.

[ece371292-bib-0105] Pratchett, M. S. , K. D. Anderson , M. O. Hoogenboom , et al. 2015. “Spatial, Temporal and Taxonomic Variation in Coral Growth‐Implications for the Structure and Function of Coral Reef Ecosystems.” Oceanography and Marine Biology: An Annual Review 53: 215–295. 10.1201/b18733.

[ece371292-bib-0106] Price, D. M. , A. Lim , A. Callaway , et al. 2021. “Fine‐Scale Heterogeneity of a Cold‐Water Coral Reef and Its Influence on the Distribution of Associated Taxa.” Frontiers in Marine Science 8: 1–20. 10.3389/fmars.2021.556313.35685121

[ece371292-bib-0107] Purkis, S. J. , A. C. R. Gleason , C. R. Purkis , et al. 2019. “High‐Resolution Habitat and Bathymetry Maps for 65,000 Sq. Km of Earth's Remotest Coral Reefs.” Coral Reefs 38: 467–488. 10.1007/s00338-019-01802-y.

[ece371292-bib-0108] Pygas, D. R. , R. Ferrari , and W. F. Figueira . 2020. “Review and Meta‐Analysis of the Importance of Remotely Sensed Habitat Structural Complexity in Marine Ecology.” Estuarine, Coastal and Shelf Science 235: 106468. 10.1016/j.ecss.2019.106468.

[ece371292-bib-0109] Quigley, K. M. , M. Hein , and D. J. Suggett . 2022. “Translating the 10 Golden Rules of Reforestation for Coral Reef Restoration.” Conservation Biology 36: 1–8. 10.1111/cobi.13890. PMC9543798 35075743

[ece371292-bib-0110] R Core Team . 2021. R: A Language and Environment for Statistical Computing .

[ece371292-bib-0111] Radford, B. , M. Puotinen , D. Sahin , N. Boutros , M. Wyatt , and J. Gilmour . 2024. “A Remote Sensing Model for Coral Recruitment Habitat.” Remote Sensing of Environment 311: 114231. 10.1016/j.rse.2024.114231.

[ece371292-bib-0112] Riginos, C. , E. D. Crandall , L. Liggins , P. Bongaerts , and E. A. Treml . 2016. “Navigating the Currents of Seascape Genomics: How Spatial Analyses Can Augment Population Genomic Studies.” Current Zoology 62: 581–601. 10.1093/cz/zow067. 29491947 PMC5804261

[ece371292-bib-0113] Roelfsema, C. M. , E. M. Kovacs , J. C. Ortiz , et al. 2020. “Habitat Maps to Enhance Monitoring and Management of the Great Barrier Reef.” Coral Reefs 39: 1039–1054. 10.1007/s00338-020-01929-3.

[ece371292-bib-0114] Rose, N. H. , R. A. Bay , M. K. Morikawa , L. Thomas , E. A. Sheets , and S. R. Palumbi . 2021. “Genomic Analysis of Distinct Bleaching Tolerances Among Cryptic Coral Species.” Proceedings of the Royal Society B: Biological Sciences 288: 20210678. 10.1098/rspb.2021.0678. PMC8511746 34641729

[ece371292-bib-0115] Rowden, A. A. , O. F. Anderson , S. E. Georgian , et al. 2017. “High‐Resolution Habitat Suitability Models for the Conservation and Management of Vulnerable Marine Ecosystems on the Louisville Seamount Chain, South Pacific Ocean.” Frontiers in Marine Science 4: 335. 10.3389/fmars.2017.00335.

[ece371292-bib-0116] Sappington, J. M. , K. M. Longshore , and D. B. Thompson . 2007. “Quantifying Landscape Ruggedness for Animal Habitat Analysis: A Case Study Using Bighorn Sheep in the Mojave Desert.” Journal of Wildlife Management 71: 1419–1426. 10.2193/2005-723.

[ece371292-bib-0117] Selmoni, O. , G. Lecellier , V. Berteaux‐Lecellier , and S. Joost . 2023. “The Reef Environment Centralized InFormation System (RECIFS): An Integrated Geo‐Environmental Database for Coral Reef Research and Conservation.” Global Ecology and Biogeography 32: 622–632. 10.1111/geb.13657.

[ece371292-bib-0118] Selmoni, O. , E. Rochat , G. Lecellier , V. Berteaux‐Lecellier , and S. Joost . 2020. “Seascape Genomics as a New Tool to Empower Coral Reef Conservation Strategies: An Example on North‐Western Pacific *Acropora digitifera* .” Evolutionary Applications 13: 1923–1938. 10.1111/eva.12944. 32908595 PMC7463334

[ece371292-bib-0119] Smith, A. B. , S. J. Murphy , D. Henderson , and K. D. Erickson . 2023. “Including Imprecisely Georeferenced Specimens Improves Accuracy of Species Distribution Models and Estimates of Niche Breadth.” Global Ecology and Biogeography 32: 342–355. 10.1111/geb.13628.

[ece371292-bib-0120] Smith, M. W. 2014. “Roughness in the Earth Sciences.” Earth‐Science Reviews 136: 202–225. 10.1016/j.earscirev.2014.05.016.

[ece371292-bib-0121] Sous, D. , S. Meulé , S. Dealbera , et al. 2024. “Quantifying the Topographical Structure of Rocky and Coral Seabeds.” PLoS One 19: 1–28. 10.1371/journal.pone.0303422. PMC11156299 38843131

[ece371292-bib-0122] Tokeshi, M. , and S. Arakaki . 2012. “Habitat Complexity in Aquatic Systems: Fractals and Beyond.” Hydrobiologia 685: 27–47. 10.1007/s10750-011-0832-z.

[ece371292-bib-0123] Tong, R. , A. Purser , V. Unnithan , and J. Guinan . 2012. “Multivariate Statistical Analysis of Distribution of Deep‐Water Gorgonian Corals in Relation to Seabed Topography on the Norwegian Margin.” PLoS One 7: 1–13. 10.1371/journal.pone.0043534. PMC3422289 22912887

[ece371292-bib-0124] Tong, R. , A. Purser , V. Unnithan , and J. Yu . 2016. “Predicting Potential Distribution for Cold‐Water Coral Based on GIS and MaxEnt.” 10.1109/GEOINFORMATICS.2015.7378689.Int. Conf. Geoinformatics 2016‐January.

[ece371292-bib-0125] Vega Thurber, R. , L. D. Mydlarz , M. Brandt , et al. 2020. “Deciphering Coral Disease Dynamics: Integrating Host, Microbiome, and the Changing Environment.” Frontiers in Ecology and Evolution 8: 1–18. 10.3389/fevo.2020.575927.

[ece371292-bib-0126] Veron, J. E. N. 2000. Corals of the World. Australian Institute of Marine Science.

[ece371292-bib-0127] Wallace, C. C. , B. J. Done , and P. R. Muir . 2012. “Revision and Catalogue of Worldwide Staghorn Corals Acropora and Isopora (Scleractinia: Acroporidae) in the Museum of Tropical Queensland.” Memoirs of the Queensland Museu Nature 57: 1–255. 10.17082/j:2204-1478-56-2.2013-42.

[ece371292-bib-0128] Whalan, S. , M. A. Abdul Wahab , S. Sprungala , A. J. Poole , and R. De Nys . 2015. “Larval Settlement: The Role of Surface Topography for Sessile Coral Reef Invertebrates.” PLoS One 10: 1–17. 10.1371/journal.pone.0117675. PMC4324781 25671562

[ece371292-bib-0129] Wilson, J. , and J. Gallant . 2000. Terrain Analysis: Principles and Applications, edited by J. P. Wilson and J. C. Gallant , 1–27. John Wiley & Sons.

[ece371292-bib-0130] Wilson, M. F. J. , B. O'Connell , C. Brown , J. C. Guinan , and A. J. Grehan . 2007. “Multiscale Terrain Analysis of Multibeam Bathymetry Data for Habitat Mapping on the Continental Slope.” Marine Geodesy 30, no. 1‐2: 3–35. 10.1080/01490410701295962.

[ece371292-bib-0131] Winship, A. J. , J. T. Thorson , M. E. Clarke , et al. 2020. “Good Practices for Species Distribution Modeling of Deep‐Sea Corals and Sponges for Resource Management: Data Collection, Analysis, Validation, and Communication.” Frontiers in Marine Science 7: 1–15. 10.3389/fmars.2020.00303.32802822

[ece371292-bib-0132] Wood, J. 1996. The Geomorphological Characterisation of Digital Elevation Models .

[ece371292-bib-0133] Woodcock, C. E. , and A. H. Strahler . 1987. “The Factor of Scale in Remote Sensing.” Remote Sensing of Environment 21: 311–332. 10.1016/0034-4257(87)90015-0.

[ece371292-bib-0134] Yuen, B. , C. E. Stuart , S. J. Pittman , S. J. Green , L. M. Henderson , and L. M. Wedding . 2023. “Habitat Suitability Models of Elkhorn Coral Provide Ecological Insight to Support Coral Reef Restoration.” Restoration Ecology 31: 1–15. 10.1111/rec.13953.

[ece371292-bib-0135] Zuo, X.‐L. , K.‐F. Yu , B.‐N. Qin , X.‐P. Duan , Z.‐F. Yao , and F.‐Z. Su . 2023. “Deriving Fine‐Scale Patterns of Sea Surface Temperature in Coral Reef Habitats Using the Landsat 8 Thermal Infrared Sensor.” Frontiers in Marine Science 10: 1293414. 10.3389/fmars.2023.1293414.

