# Supplementary material for: Harnessing Multiscale Topographic Environmental Variables for Regional Coral Species Distribution Models
Source: Ecol Evol. 2025 Apr 23;15(4):e71292. doi: 10.1002/ece3.71292 (PMC12017900; doi:10.1002/ece3.71292)
Supplement: Supplementary file 1 — Appendix S1. [file ECE3-15-e71292-s001.pdf]

Supporting information for:

Harnessing multiscale topographic environmental variables for regional coral species distribution models

Annie S. Guillaume [1,*], Renata Ferrari [2], Oliver Selmoni [3,4], Véronique J.L. Mocellin [2], Hugo Denis [5,6,7], Melissa Naugle [7], Emily Howells [7], Line K. Bay [2], Stéphane Joost [1,*]

[1] Geospatial Molecular Epidemiology group (GEOME), Laboratory for Biological Geochemistry (LGB), Ecole Polytechnique Fédérale de Lausanne (EPFL), Station 2, CH-1015 Lausanne, Switzerland

[2] Australian Institute of Marine Science, Townsville, QLD, Australia

[3] Department of Embryology, Carnegie Institution for Science, Baltimore, MD, USA

[4] Department of Plant Biology, Carnegie Institution for Science, Stanford, CA, USA

[5] UMR250/9220 ENTROPIE (IRD-CNRS-UR-IFREMER-UNC), Promenade Roger-Laroque, Noumea Cedex, New Caledonia, France

[6] ED 129, SU Sorbonne Université, 4, Place Jussieu, 75252 Paris, France

[7] National Marine Science Centre, Faculty of Science and Engineering, Southern Cross University, Coffs Harbour, NSW, Australia

* Corresponding author: annie.guillaume@alumni.epfl.ch, stephane.joost@epfl.ch

---

Summary of supporting information

Supplementary results: Topographic derived variable importance per species

Figure S1: Quantile-quantile (QQ) plots for digital elevation model (DEM) vertical error

Figure S2: Normalized median absolute deviation (NMAD) of digital elevation model (DEM) vertical error

Figure S3: Correlation plot (Spearman correlation r_s) between pairs of independent derived variables at all spatial resolutions

Figure S4: Performance evaluation of MaxEnt species distribution models, assessed at reefs across the entire GBR extent, highlighting variance between cross-validations.

Figure S5: Full Jackknife assessment results (**a.** *A. hyacinthus*, **b.** *A. spathulata*, **c.**, *A. kenti*)

Figure S6: MaxEnt marginal response curves for all variables (**a.** *A. hyacinthus*, **b.** *A. spathulata*, **c.**, *A. kenti*)

Figure S7: Individual variable response curves (**a.** *A. hyacinthus*, **b.** *A. spathulata*, **c.**, *A. kenti*)

Table S1: Summary of sampled reefs with regards to location and colonies sampled

Table S2: Summary of benthic habitat and coral reef geomorphic information based on the Allen Coral Atlas for sampled colonies

Table S3: Description and parameters for bathymetry and eight DEM derived variables

Table S4: Summary results ranking MaxEnt species distribution models to determine optimal parameters

Table S5: Summary statistics of digital elevation models (DEM) vertical error

Table S6: Statistical analyses of comparison model test scores between species

Table S7: Statistical analyses of comparison model test scores between source DEMs for each species

Supplementary Results

Topographic derived variable importance per species

MaxEnt models revealed many similarities in topographic variable importance amongst the three *Acropora* species, where the general trends are discussed in the main text. Subtle differences in the contribution of these variables for each species appear upon close investigation, which are discussed here.

A. hyacinthus had 13 top predictor variables according to jackknife analyses (**Figure 6a**), of which eight were slope at all spatial resolutions. Depth was missing as a top predictor, with AUC_{ONLY} values between 0.6–0.7 for DEM at 30-60m (**Supp Figure S5a**). This indicates that depth alone is not a strong predictor for this species at these resolutions. Slope, however, was an extremely strong predictor for *A. hyacinthus* across multiple resolutions, most strongly at 30m and 60m (**Supp Figure S5a**). The integration of eastness and northness produced models no different than random. Only eastness at the coarsest resolution of 120m contributed to MaxEnt models, where *A. hyacinthus* was more likely to occur on the western reef slopes (**Supp Figure S6a**). Curvature was only important in the profile direction (VCU), where at 30m moderately concave slopes were very important. Terrain complexity, measured as VRM, did not appear to be useful in modelling *A. hyacinthus*, except for a slightly negative relationship with VRM at 30m, indicating that this species can be found across a variety of terrain complexities. However, it appears that this species strongly prefers peaks, with a strong positive relationship between occurrence and positive BPI values at 15m. SVF was not included in the model at all (**Supp Figure S5a**).

A. spathulata had the most predictor variables, with 20 top variables that had acceptable levels of discrimination when used alone in models based on jackknife analyses (**Figure 6b**). As with *A. hyacinthus*, slope at all eight resolutions had strong predictive power (**Supp Figure S5b**). Depth was a strong predictor for this species at the provided resolutions, especially at 15–60m, potentially because this species is more generalist with regards to depth. Slope was again a strong predictor, particularly at 30-120m (**Supp Figure S5b**). Though eastness and northness produced models only slightly better than random, response curves indicate a slight preference for north-east slopes at fine resolutions, and an overall preference for south-eastern reef slopes (**Supp Figure S6b**). Curvature was only important in the profile direction (VCU), where at 30–60m moderately concave slopes were very important. VRM did not appear to be useful in modelling *A. spathulata*, except for a strong negative relationship with VRM at 60m, indicating that this species prefers generally smoother terrain. However, there is a strong preference for local peaks and reef crests at fine resolutions, with a strong positive relationship between occurrence and positive BPI values at 15–60m (**Supp Figure S6b**). The amount of light obstruction, measured as SVF, had little impact on the location of *A. spathulata* colonies, with a slight increase in predicted presence in large areas of open sky (SVF at 100m).

A. kenti had 19 top variables (**Figure 6c**). Depth was generally a relatively weaker predictor of species occurrence, with AUC_{ONLY} values between 0.6–0.7 for depth at 60-120m. The exception was depth at the finer resolution of 15m, where deeper waters predicted higher species presence (**Supp Figure S5c**). Slope was again a strong predictor, particularly at 60-120m (**Supp Figure S5b**), but ACA120m was not included in the analyses. Again, eastness and northness produced models only slightly better than random, where response curves indicate a slight preference for northern slopes at fine resolutions, and an overall preference for southern reef slopes (**Supp Figure S6c**). Curvature was again only important in the profile direction (VCU), where at 30–60m moderately concave slopes were very important. VRM at 30–60m had a strong negative relationship with species presence, with a slightly positive relationship at 15, indicating a preference for some small-scale variations and large scale smoothness of terrain. There is also a strong preference for local peaks and reef crests at fine resolutions, with a strong positive relationship between occurrence and positive BPI values at 15–30m (**Supp Figure S6c**). SVF had little impact on the location of *A. spathulata* colonies, with slight decrease in predicted occurrence when in open areas at fine and broad scales (SVF 15m and 120m).

Supp Figure S1. Quantile-quantile (Q-Q) plots assessing the vertical error (Δh; in meters) of the bathymetry digital elevation models (DEM) at multiple spatial resolutions, derived from three publicly available sources: Allen Coral Atlas 15m, DeepReef 30m, and DeepReef 100m. Vertical error (Δh; in meters) was calculated as the difference between the tide-corrected depth of sampled colonies (n=2368) and the predicted depth from the bathymetry models. Bathymetry source and spatial resolutions are noted at the top of each plot.

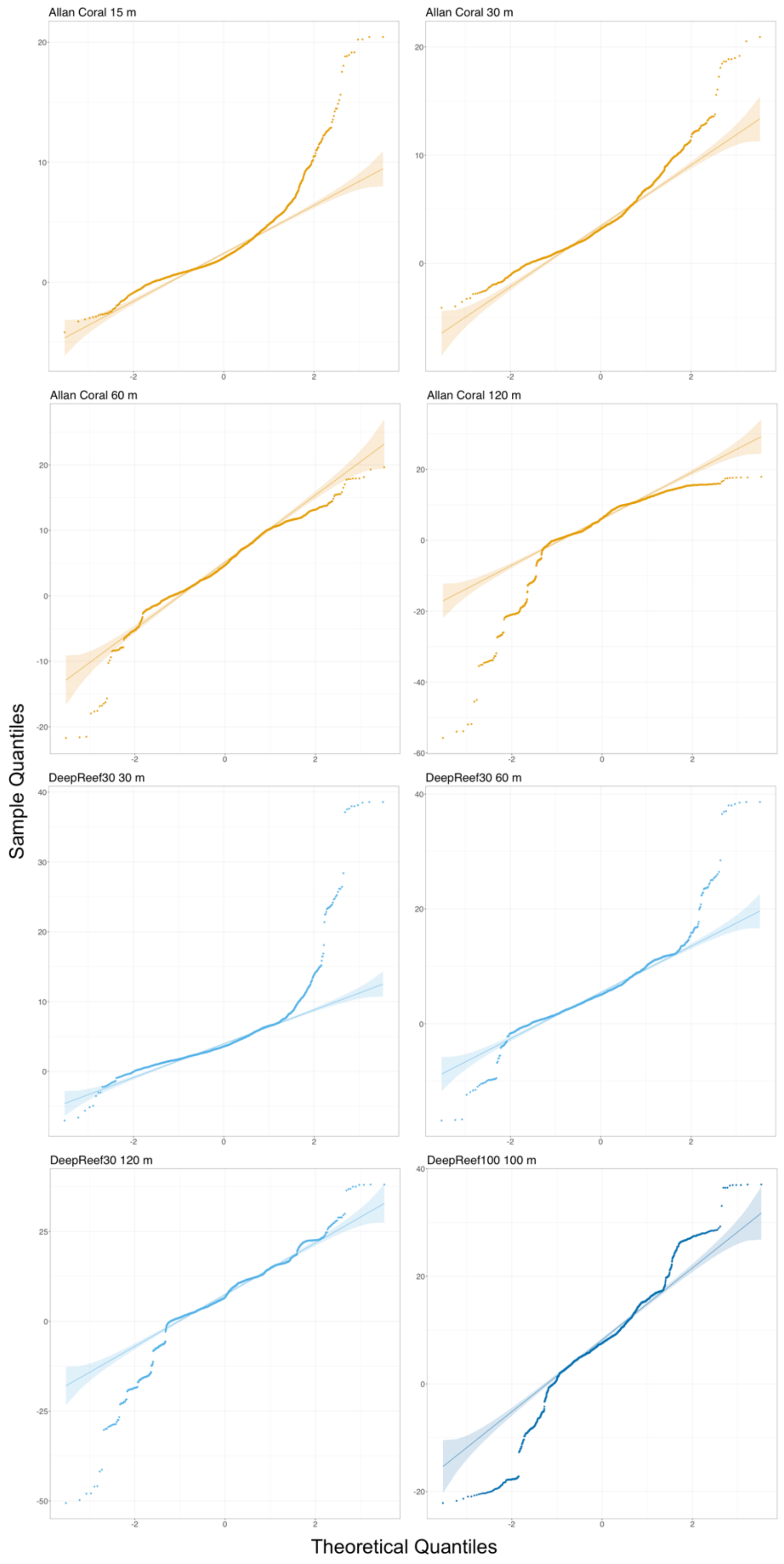

Supp Figure S2. Normalised median absolute deviation (NMAD; meters) for the vertical error (Δh; in meters) of the bathymetry digital elevation models (DEM) at multiple spatial resolutions, derived from three publicly available sources: Allen Coral Atlas 15m (ACA 15m; orange), DeepReef 30m (light blue), and DeepReef 100m (dark blue). Vertical error (Δh; in meters) was calculated as the difference between the tide-corrected depth of sampled colonies (n=2368) and the predicted depth from the bathymetry models.

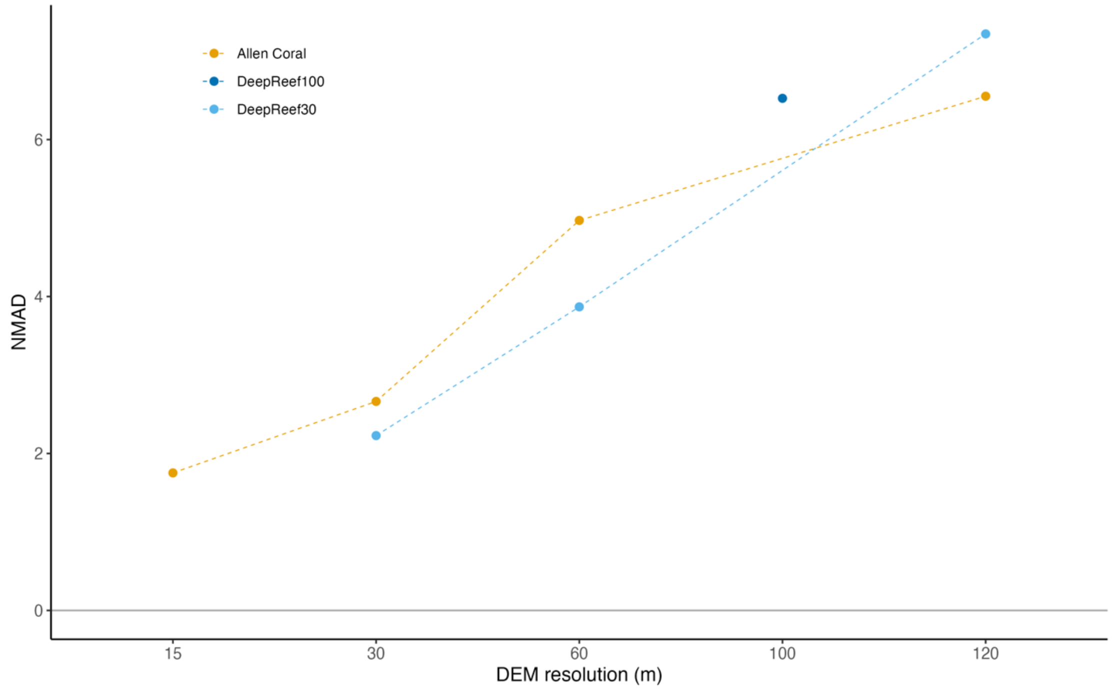

Supp Figure S3. Spearman correlation (r_s) for pairwise comparisons of all variables (9 types * 8 source-resolutions; n=72) derived from bathymetric digital elevation models (DEMs) retained after initial correlation analyses, assessed at 15,000 random points across 23 sampled reefs of the Great Barrier Reef. Bathymetric DEMs were obtained from three publicly available sources: Allen Coral Atlas 15m (ACA15), DeepReef30m, and DeepReef100m. Abbreviations and information on variables are provided in **Supp Table S3**.

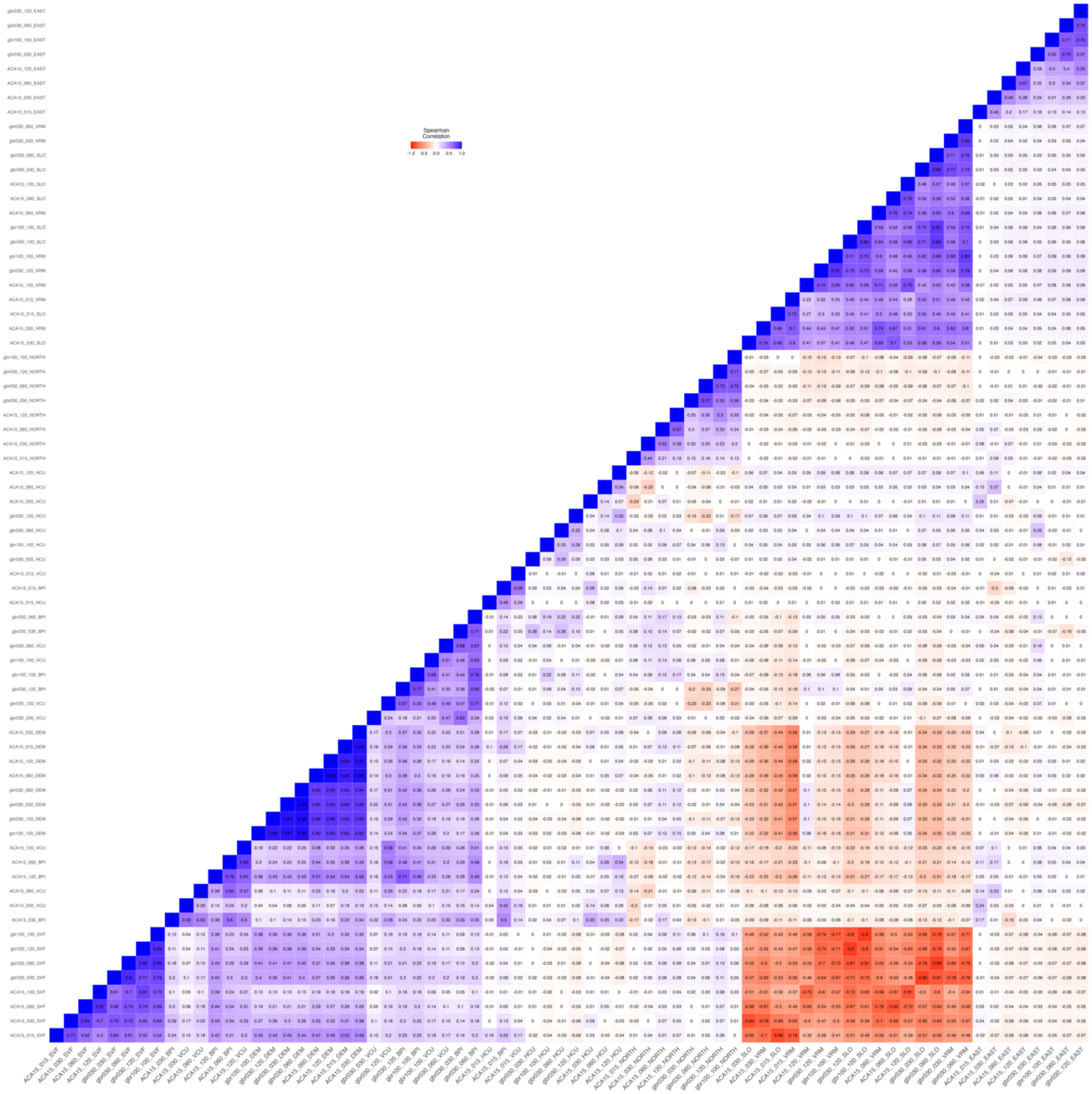

Supp Figure S4. Performance evaluation of MaxEnt species distribution models (SDMs) for three *Acropora* species (*A. hyacinthus*, *A. spathulata*, and *A. kenti*) assessed at reefs of the Great Barrier Reef, compared using two metrics: (**a**) AUC_{TEST}, and (**b**) BIC. These plots highlight the variability in the leave-one-reef-out cross validation method used to evaluate MaxEnt models, where each cross-validation was repeated over ten iterations per excluded reef using different selections of 75% of occurrence points to train the models.

The x-axes represent the spatial resolution of predictor variables used to build SDMs, with violin plots coloured by the source of DEM bathymetry models: Allen Coral Atlas 15m (ACA 15m; orange), DeepReef 30m (light blue), DeepReef 100m (dark blue). The models built with all variable types at all spatial resolutions from all sources (n=72) is indicated in grey. Points are coloured by the reef that was left out in the model.

(a) AUC_{TEST}

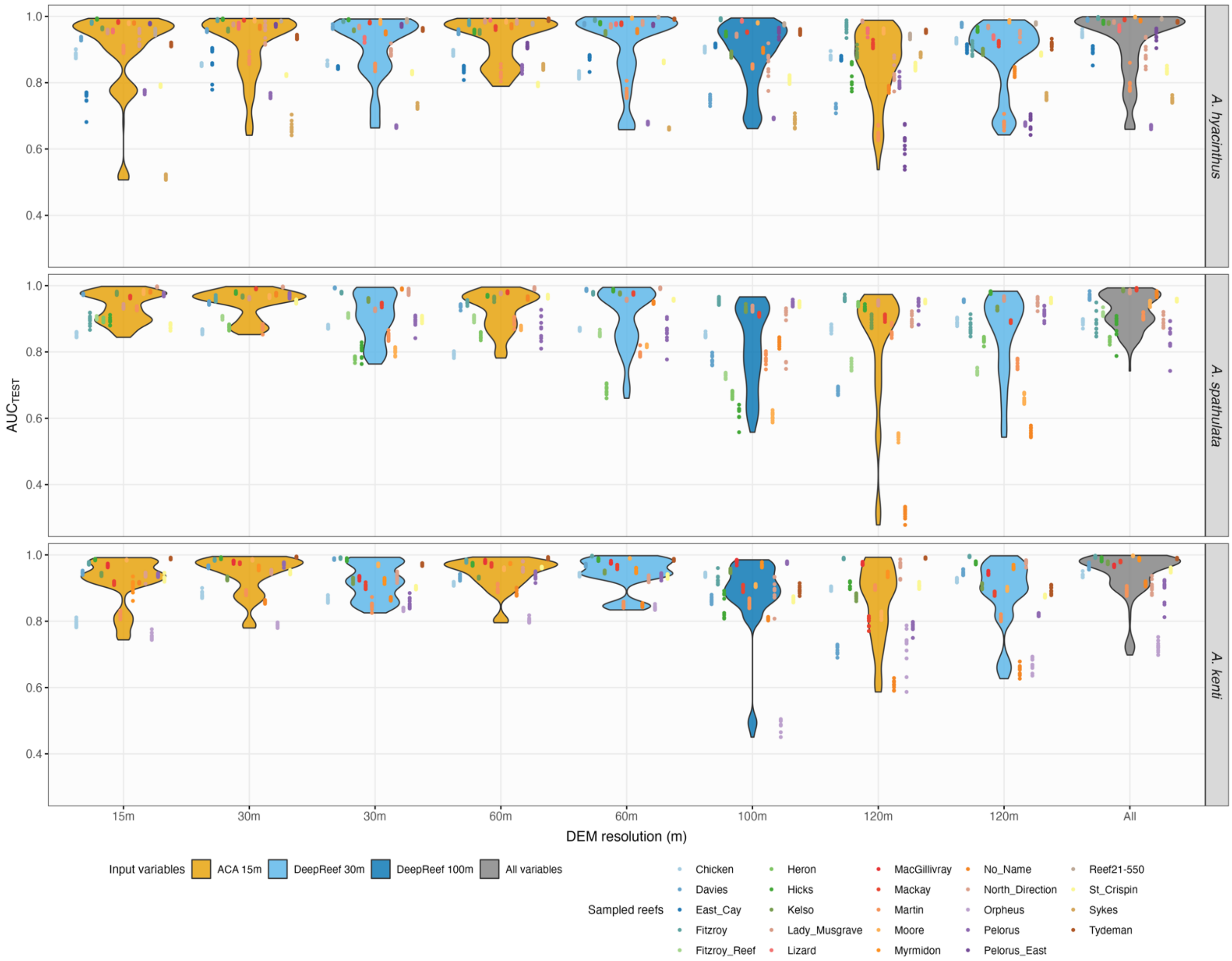

(b) BIC

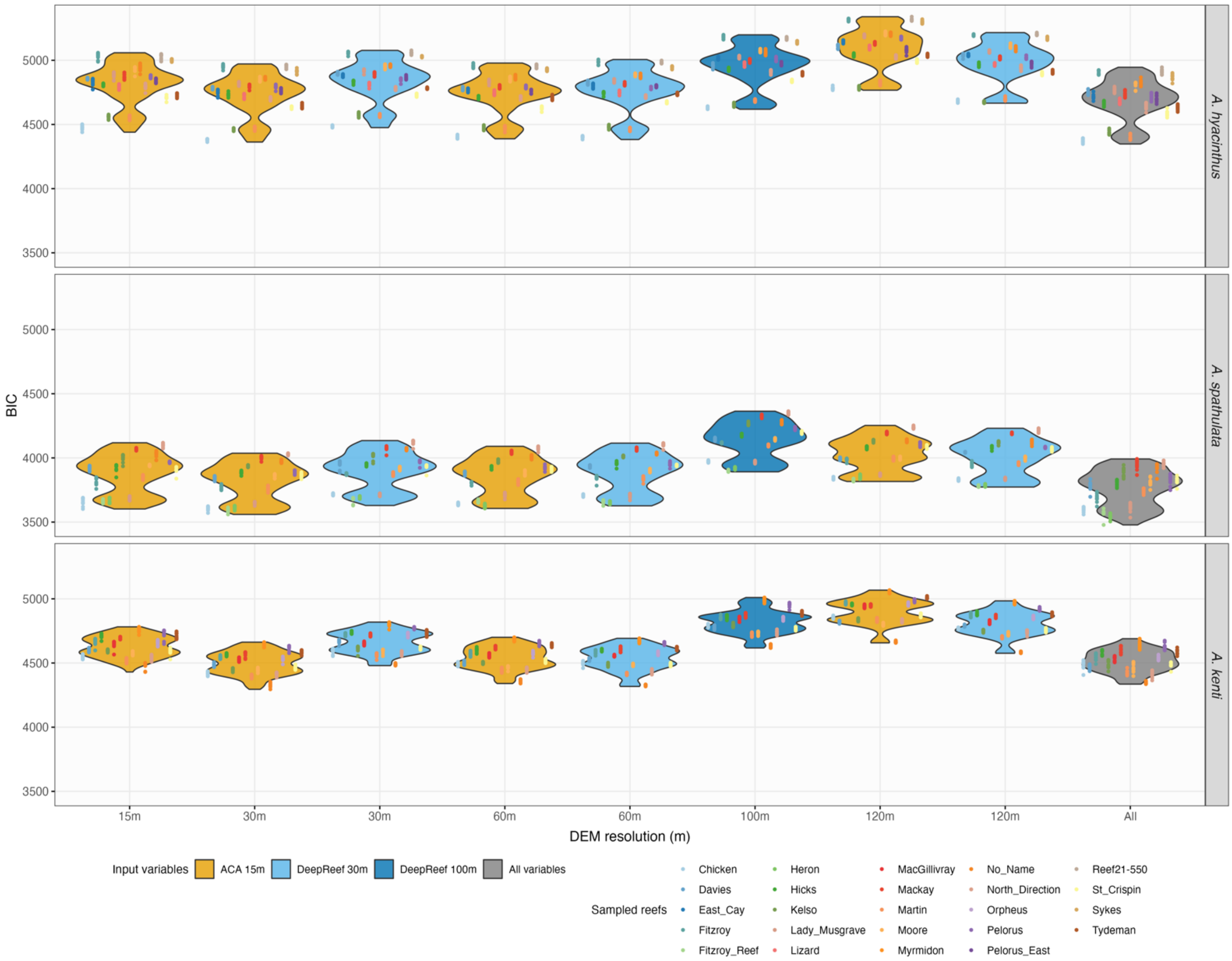

BIC
5000
4500
4000
3500
A. hyacinthus
A. spathulata
A. kenti
15m
30m
30m
60m
60m
100m
120m
120m
All
DEM resolution (m)
Input variables
ACA 15m
DeepReef 30m
DeepReef 100m
All variables
Sampled reefs
Chicken
Davies
East_Cay
Fitzroy
Fitzroy_Reef
Heron
Hicks
Kelso
Lady_Musgrave
Lizard
MacGillivray
Mackay
Martin
Moore
Myrmidon
No_Name
North_Direction
Orpheus
Pelorus
Pelorus_East
Reef21-550
St_Crispin
Sykes
Tydeman

Supp Figure S5. The relative importance of all predictor variables used in distribution modelling of three *Acropora* coral species: (**a**) *A. hyacinthus*, (**b**) *A. spathulata,* and (**c**) *A. kenti*, across 23 sampled reefs of the Great Barrier Reef. Jackknife assessments highlight each variable's importance by comparing full model AUC (AUC_{TOTAL}; red dashed lines), with the AUC of a model run with only a target variable (AUC_{ONLY}; dark blue bars) and with all variables except for the target ($AUC_{WITHOUT}$; grey bars). Abbreviations and information on variables are provided in **Supp Table S3**. Rows of variables without bars indicate that MaxEnt has automatically removed the variable during modelling due to collinearity with other variables.

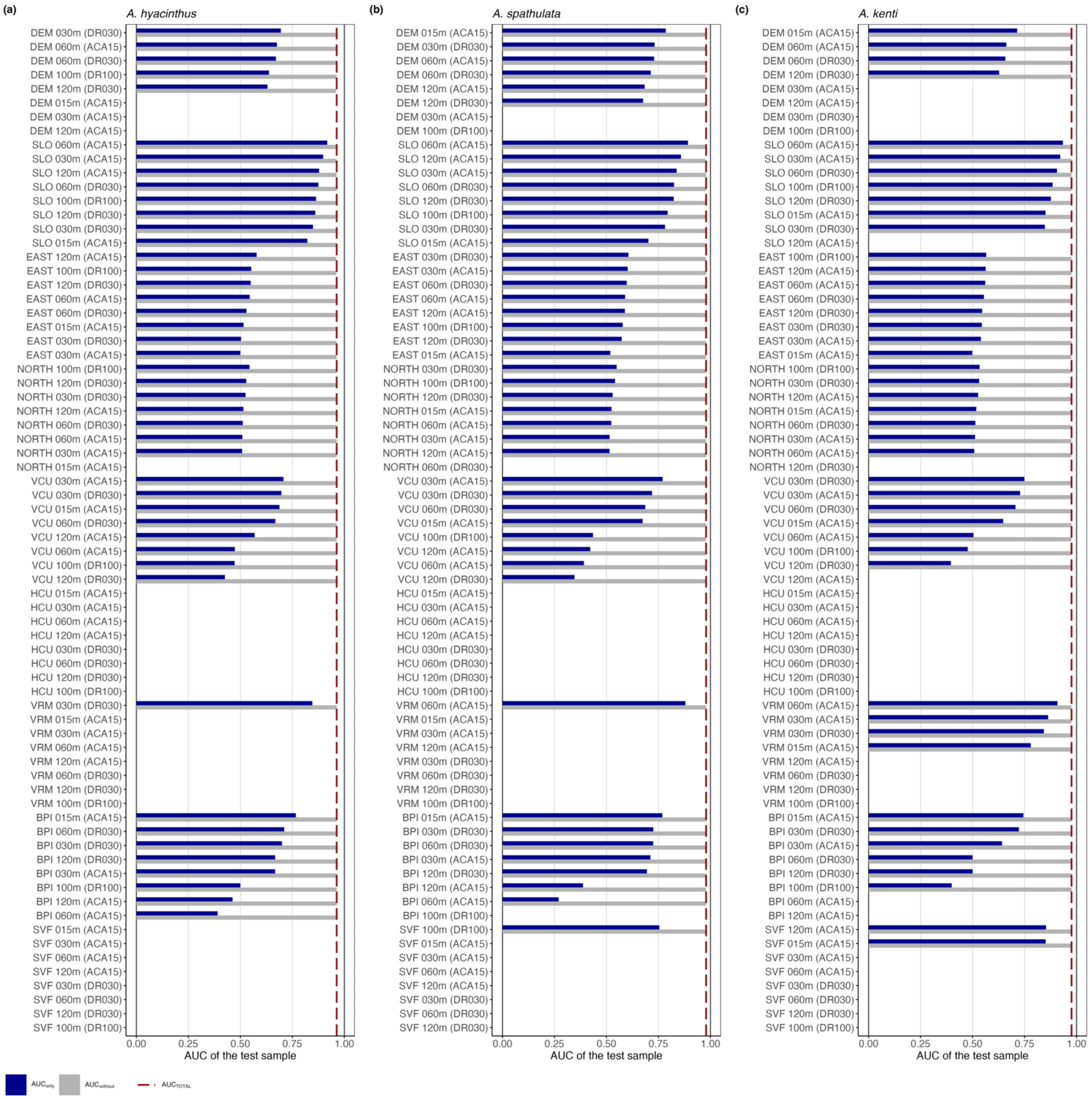

Supp Figure S6: Marginal response plots for the 72 predictor variables used in MaxEnt models of the three *Acropora* species: (**a**) *A. hyacinthus*, (**b**) *A. spathulata,* and (**c**) *A. kenti.* Each plot highlights how the predicted probability of occurrence (y-axis) varies at different values of the target variable (x-axis) once the effect of all other variables has been accounted for (using the average sampled values of all other variables). The plots use standardized covariates with a mean of zero for comparisons across covariates with differing ranges.
Here, variable type is sorted by row (DEM=digital elevation model; Slope; EAST=eastness; NORTH=northness; VCU=Vertical curvature; HCU=horizontal curvature; VRM=Vector ruggedness measure; BPI=Bathymetric position index; SVF=sky view factor; **Supp Table S3**), while bathymetric source is sorted by column (ACA=Allen Coral Atlas; DR=DeepReef; each at multiple spatial resolutions). Note that the scales on the x-axes represent the range of values unique to each variable and are not standardised.

(a) ***A. hyacinthus***

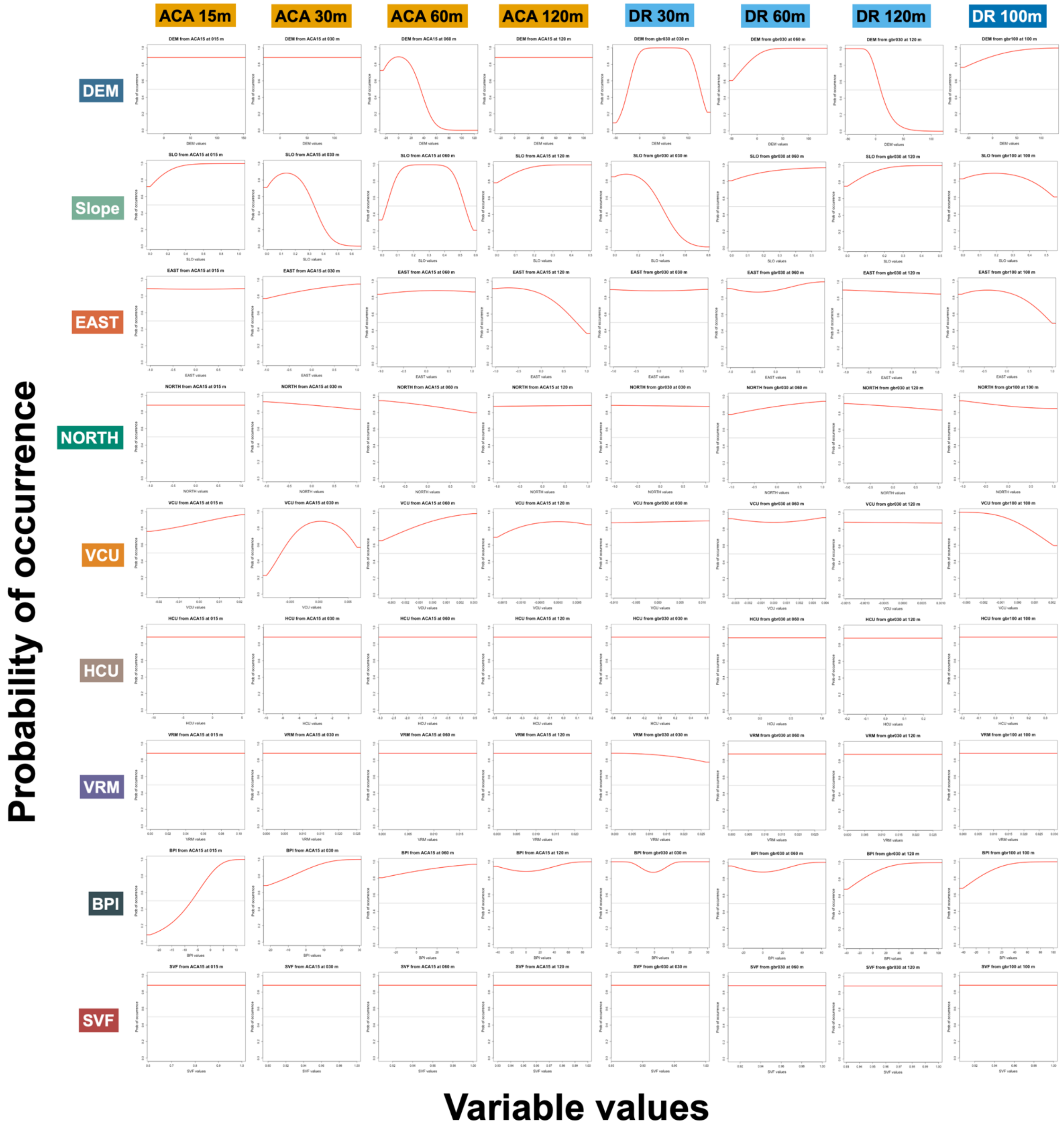

(b) ***A. spathulata***

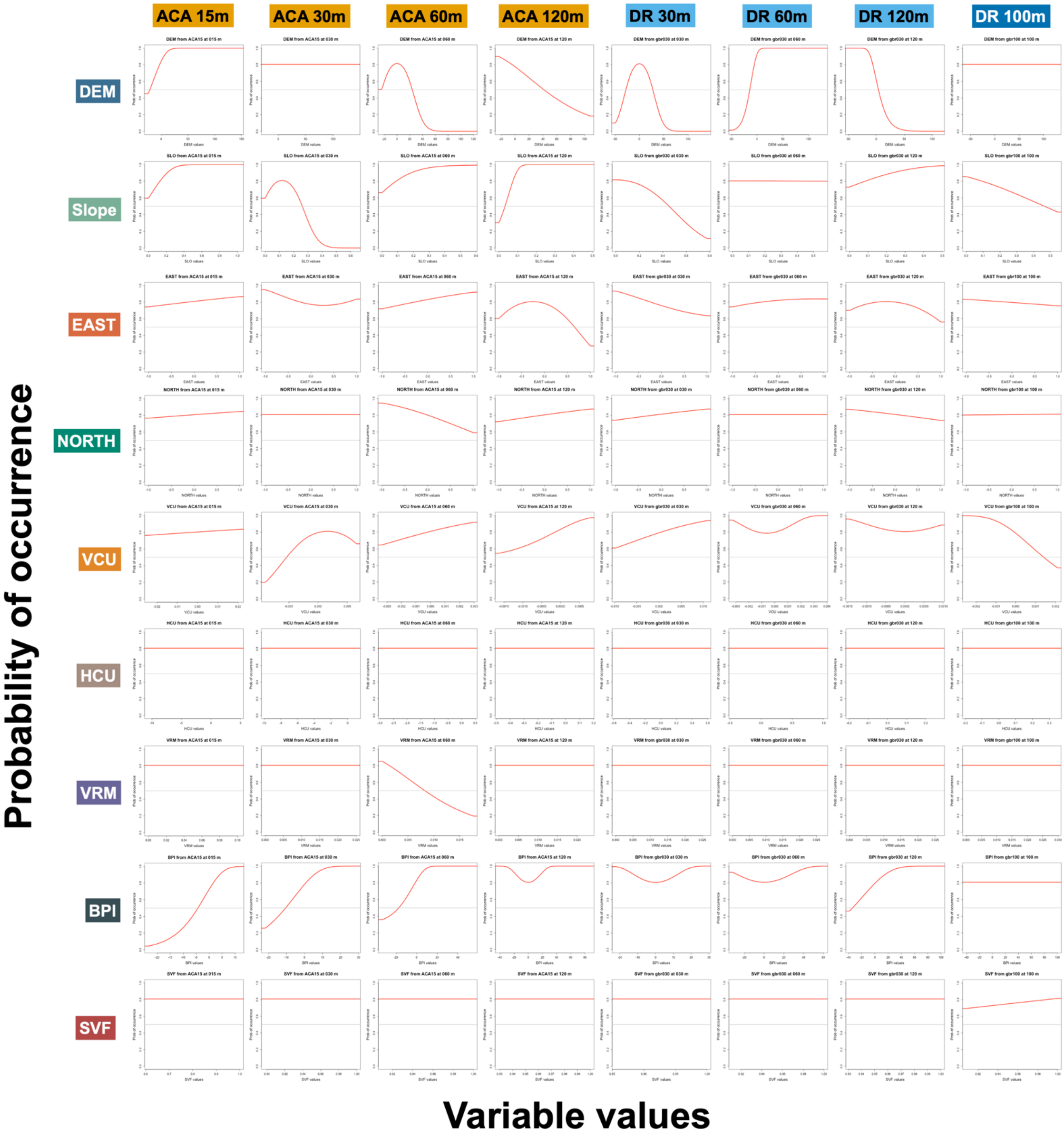

(c) ***A. kenti***

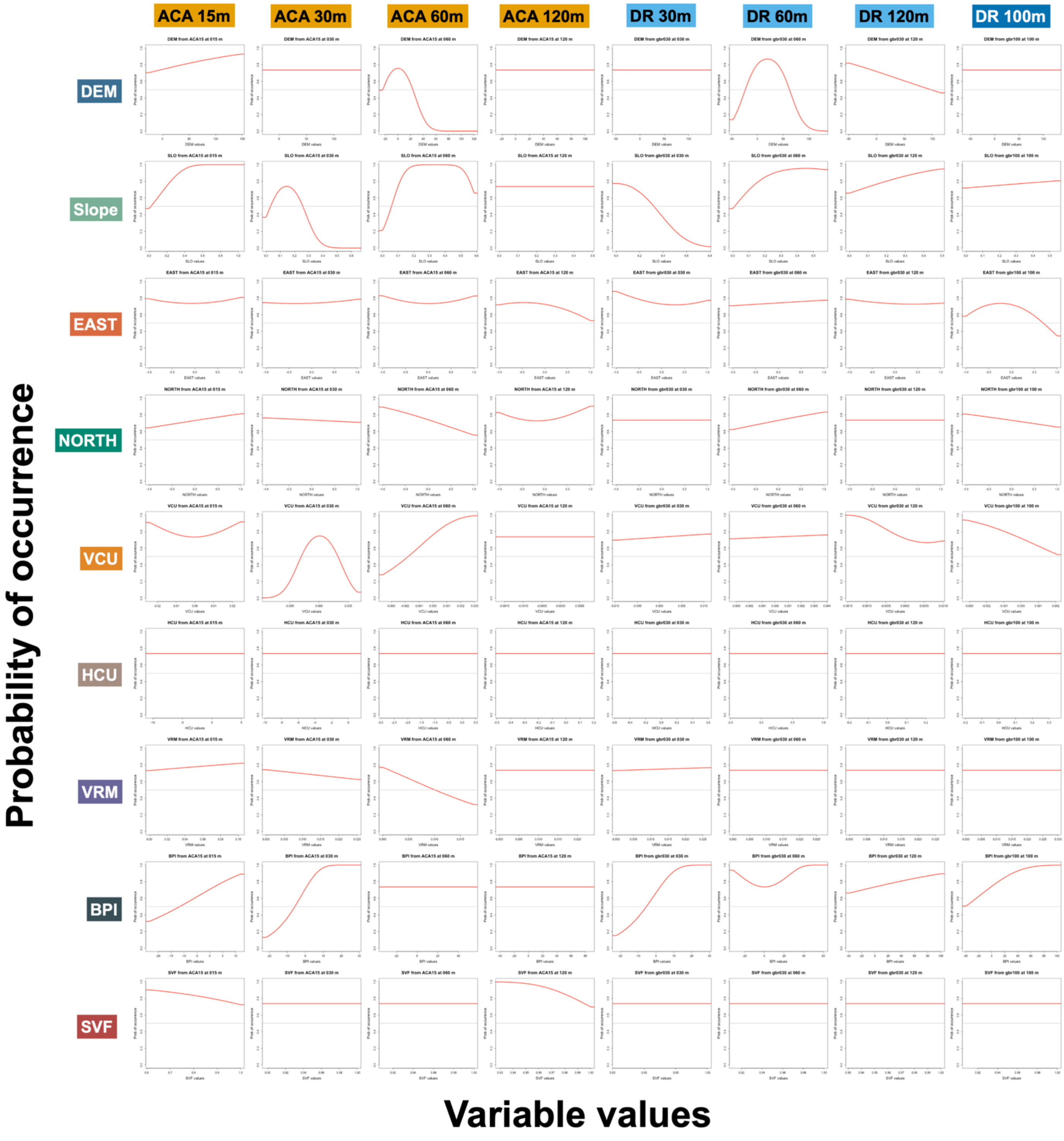
ACA 15m
ACA 30m
ACA 60m
ACA 120m
DR 30m
DR 60m
DR 120m
DR 100m
DEM
Slope
EAST
NORTH
VCU
HCU
VRM
BPI
SVF
Probability of occurrence
Variable values

Supp Figure S7: Individual variable response plots for the 72 predictor variables used in MaxEnt models of the three *Acropora* species: (**a**) *A. hyacinthus*, (**b**) *A. spathulata,* and (**c**) *A. kenti.* Each plot highlights how the predicted probability of occurrence (y-axis) varies at different values of the target variable (x-axis) when the MaxEnt model is performed only with that variable. These differ from **Supp Figure S6** in that no other variable is considered in the model. This is useful as there are some correlations amongst predictor variables, complicating interpretation of variables in models from **Supp Figure S6**.

Here, variable type is sorted by row (DEM=digital elevation model (ie, depth); Slope; EAST=eastness; NORTH=northness; VCU=Vertical curvature; HCU=horizontal curvature; VRM=Vector ruggedness measure; BPI=Bathymetric position index; SVF=sky view factor; **Supp Table S3**), while bathymetric source is sorted by column (ACA=Allen Coral Atlas; DR=DeepReef; each at multiple spatial resolutions). Note that the scales on the x-axes represent the range of values unique to each variable and are not standardised.

(a) ***A. hyacinthus***

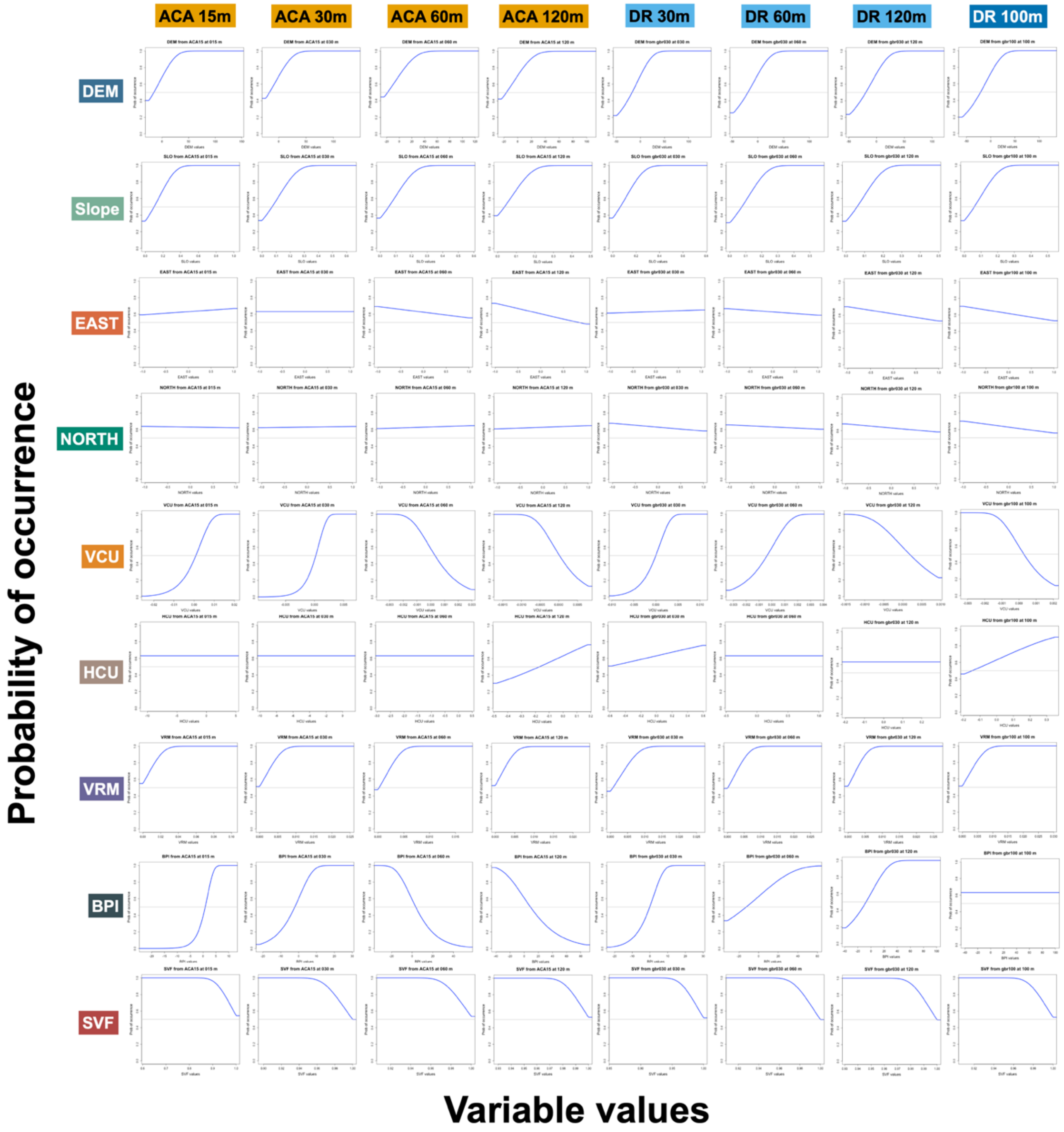

(b) ***A. spathulata***

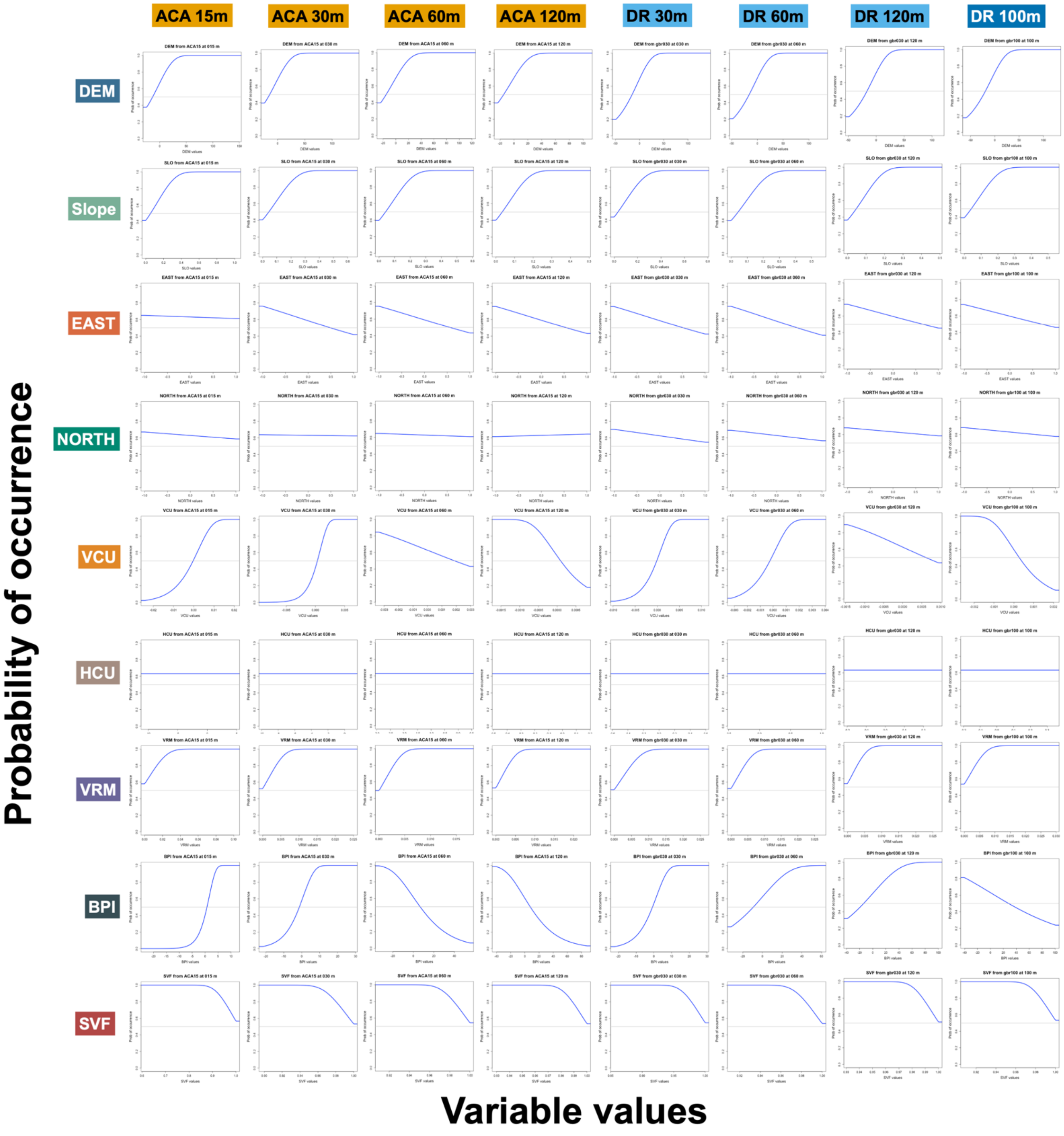

(c) *A. kenti*

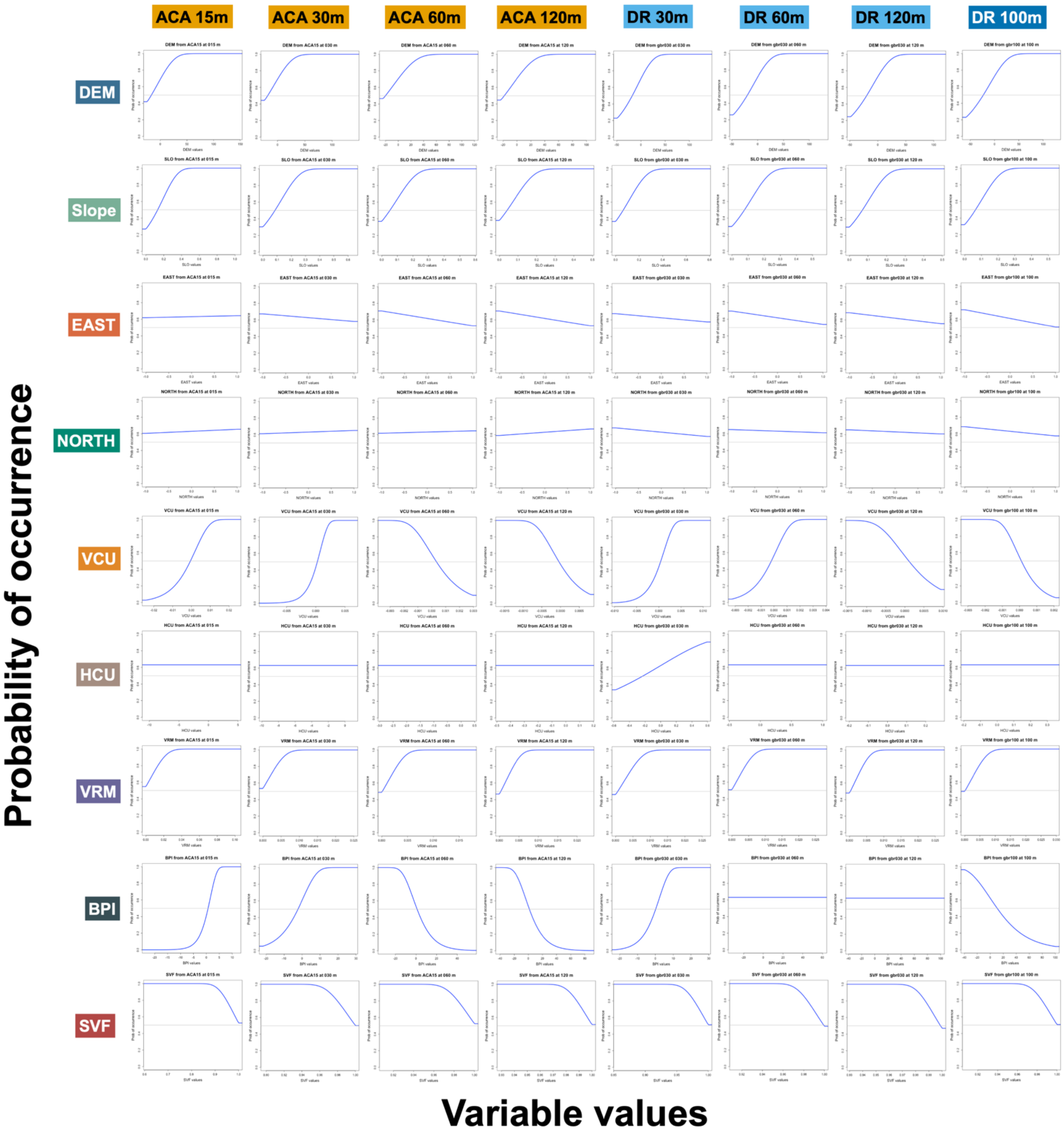
ACA 15m
ACA 30m
ACA 60m
ACA 120m
DR 30m
DR 60m
DR 120m
DR 100m
DEM
Slope
EAST
NORTH
VCU
HCU
VRM
BPI
SVF
Probability of occurrence
Variable values

Supp Table S1. Summary of occurrence data for the three *Acropora* coral species (*A. hyacinthus, A. spathulata* and *A. kenti*) assessed across 23 reefs sampled in the Great Barrier Reef. Summarised in the table: average latitude and longitude from all sampled colonies at the reef; the nearest tide station from which tide information was obtained to correct sampled colony depths; the range of tide-corrected depths of colonies and the distance between the reef and its closest tide station; and the total number of georeferenced colonies, for each species group. Positive depths indicate colonies situated above the lowest astronomical tide level. The number of unique colonies retained per 15m grid used in MaxEnt modelling are provided at the bottom of the table for each species.

Reef	Latitude	Longitude	Tide station	Dist. reef to tide station	Depth range	*A. hyacinthus*	*A. spathulata*	*A. kenti*	Total colonies
Chicken	-18.6683	147.7071	Dip Reef	39 km	-5.7 to 0.6 m	103	60	60	**223**
Davies	-18.8259	147.6266	Shrimp Reef	46 km	-5.9 to 0.4 m	63	53	44	**160**
East Cay	-21.4709	152.5632	East Cay	0 km	-3.1 to 0.5 m	15			**15**
Fitzroy Island	-16.9398	145.9851	Fitzroy Island	0 km	-3.5 to 0.6 m	4	60	57	**121**
Fitzroy Reef	-23.6128	152.1514	One Tree Island	13 km	-0.7 to 0.8 m		61		**61**
Heron	-23.4397	151.9811	Heron Island	0 km	-1 to 1.3 m		60		**60**
Hicks	-14.4726	145.5004	Carter Reef	13 km	-5.9 to 0.2 m	43	61	41	**145**
Kelso	-18.4269	146.9865	Rib Reef	13 km	-6.6 to -0.5 m	54	44	51	**149**
Lady Musgrave	-23.9048	152.3906	Lady Musgrave	0 km	-4.2 to 1.1 m	24	55		**79**
Lizard	-14.6462	145.4559	Lizard Island	0 km	-4.2 to 0 m	50		40	**90**
Mackay	-16.0387	145.6514	Sylvan Reef	9 km	-1.7 to 1.1 m	53	60	56	**169**
Martin	-14.7801	145.3590	Lizard Island	16 km	-4.1 to 0.2 m	49	60	59	**168**

Reef	Latitude	Longitude	Tide station	Dist. reef to tide station	Depth range	*A. hyacinthus*	*A. spathulata*	*A. kenti*	Total colonies
Moore	-16.8658	146.2104	Sudbury Cay	10 km	-7.7 to 0.3 m	51	44	43	**138**
Myrmidon	-18.2780	147.3790	Dip Reef	17 km	-5.2 to -0.6 m			25	**25**
No Name	-14.6504	145.6290	Carter Reef	12 km	-5.7 to 0.3 m	57	53	61	**171**
North Direction	-14.7428	145.5125	North Direction	0 km	-2.6 to 1 m	46	52	60	**158**
Orpheus	-18.5667	146.4889	Lucinda Offshore	16km	-1.3 to 0.5 m			30	**30**
Pelorus	-18.5575	146.5000	Lucinda Offshore	12 km	-5.5 to 0.6 m	22	45	60	**127**
Pelorus East	-18.5587	146.5024	Lucinda Offshore	13 km	-2 to 1.8 m	30			**30**
Reef21-550	-21.9604	152.3038	Gannet Cay	17 km	-3.5 to 1.6 m	5			**5**
St Crispin	-16.0748	145.8436	Michaelmas Cay	61 km	-3.5 to 0.9 m	44	60	59	**163**
Sykes	-23.4458	152.0262	One Tree Island	9 km	-1.3 to -0.1 m	5			**5**
Tydeman	-13.9713	144.5051	Pipon Islands	16 km	-5.5 to 0.1 m	50		26	**76**
**Total occurrence records**						**768**	**828**	**772**	**2368**
**Total occurrence records after thinning for MaxEnt**						**329**	**269**	**324**	**922**
**Min and max tide-corrected depths**						-4.6 to 1.8m	-5.3 to 1.3m	-7.7 to 1.0m	

Supplementary Table S2. Summary of shallow coral reef benthic and geomorphic map classes attributed to each georeferenced coral colony based on the Allen Coral Atlas (ACA; Allen Coral Atlas, 2022). The global-scale ACA coral reef habitat maps are calculated from satellite imagery at 3.7m spatial resolution combined with wave modelling and ecological data. It provides six benthic classes to describe coral reef bottom types (including living organisms and substrate), as well as twelve geomorphic zones describing reef structure (only eight classes were relevant to the sampled colonies). Percentages of the number of sampled colonies allocated to a habitat type for a given species are provided in brackets.

**Benthic**	***A. hyacinthus***	***A. spathulata***	***A. kenti***	**Total colonies**
Coral/Algae	223 (29 %)	228 (27.5 %)	181 (23.4 %)	632 (26.7%)
Rock	460 (59.9 %)	451 (54.5 %)	351 (45.5 %)	1262 (53.3%)
Seagrass	-	-	5 (0.6 %)	5 (0.2%)
Rubble	4 (0.5 %)	2 (0.2 %)	20 (2.6 %)	26 (1.1%)
Sand	9 (1.2 %)	55 (6.6 %)	41 (5.3 %)	105 (4.4%)
NA	*72 (9.3 %)*	*92 (11.1 %)*	*174 (22.5 %)*	338 (14.3%)
**Geomorphic**				
Back Reef Slope	3 (0.4 %)	2 (0.2 %)	19 (2.5 %)	24 (1%)
Deep Lagoon	24 (3.1 %)	48 (5.8 %)	41 (5.3 %)	113 (4.8%)
Inner Reef Flat	10 (1.3 %)	33 (4 %)	-	43 (1.8%)
Outer Reef Flat	84 (10.9 %)	216 (26.1 %)	95 (12.3 %)	395 (16.7%)
Plateau	26 (3.4 %)	6 (0.7 %)	-	32 (1.4%)
Reef Crest	30 (3.9 %)	49 (5.9 %)	13 (1.7 %)	92 (3.9%)
Reef Slope	366 (47.7 %)	223 (26.9 %)	312 (40.4 %)	901 (38%)
Sheltered Reef Slope	168 (21.9 %)	160 (19.3 %)	127 (16.5 %)	455 (19.2%)
NA	*57 (7.4 %)*	*91 (11 %)*	*165 (21.4 %)*	313 (13.2%)
**Totals**	**768**	**828**	**772**	**2368**

Supplementary Table S3. Description and parameters for bathymetry and eight synthetic topographic environmental variables derived from digital elevation models (DEMs) obtained from two publicly available online sources: Allen Coral Atlas and DeepReef. These variables were selected due to their common usage in ecological studies and their potential for importance for coral biology. Aspect was converted into Eastness and Northness. Each variable was computed at each resolution for the Allen Coral Atlas (15, 30, 60, 120m) and DeepReef (30, 60, and 120m; 100m). Computations were performed using SAGA GIS through R, except for BPI that was computed using the *MultiscaleDTM* R package. Relevant references are located below the table.

Variable	Abbv.	Description	Units	Parameters/Reference
Bathymetry	**DEM**	DEM of the bathymetry (depth below sea level) obtained from the Allen Coral Atlas (Allen Coral Atlas 2022) or DeepReef (Beaman 2010) online resources.<br>DEM=0 → sea level<br>DEM<0 → below sea level	Meters below sea level	Generalised to multiple resolutions using Gaussian pyramid transformation. (Kalbermatten et al 2012)
Slope	**SLOPE**	*Morphometry*. Maximum rate of change in DEM. Proxy for water flow, wave direction, wave intensity, erosion, solar radiation, currents, nutrition, etc. Affects habitat formation (e.g. sand, reef flat, reef crests, strata, etc.).<br>Slope= 0 rad =0° → completely flat surface<br>Slope= 1 rad =57.2° → steep slope	Radians	Method = 9 parameter 2nd order polynomial; unit slope = radian; unit aspect = radian<br>(Zevenbergen & Thorne, 1987)
Aspect<br>Eastness<br>Northness	 **EAST**<br>**NORTH**	*Morphometry*. Orientation of the slope in the downward direction, that describes energy modification across terrain: e.g., wave direction, erosion, light, currents. Eastness and Northness represent the sine and cosine of Aspect, respectively.<br>EAST=-1 → west-facing; EAST=1 → east-facing;<br>EAST=0 → north- or south-facing<br>NORTH=-1 → south-facing; NORTH =1 → north-facing;<br>NORTH=0 → east- or west-facing	Radians	

Variable	Abbv.	Description	Units	Parameters/Reference
Vertical (profile) curvature<br><br>Horizontal (plan) curvature	**VCU**<br><br>**HCU**	*Morphometry*. The level of convexity or concavity, either parallel (VCU) or perpendicular (HCU) to slope. Important for understanding variations in terrain and complexity and quantifying the energy modification across terrain, where VCU explains acceleration or deceleration of flow across terrain, while HCU explains convergence or divergence of flow. TCU combines VCU and HCU to understand total flow over a surface.<br>VCU<0 → upwardly convex<br>VCU=0 → linear surface<br>VCU>0 → upwardly concave<br>HCU<0 → sideward concave<br>HCU=0 → linear surface<br>HCU>0 → sideward convex	Radians m^{-1}	<u>Method</u> = 9 parameter 2nd order polynomial; <u>unit slope</u> = radian; <u>unit aspect</u> = radian<br><br>(Zevenbergen & Thorne, 1987)
Vector ruggedness measure	**VRM**	*Morphometry*. Quantifies local variation in seabed rugosity with less correlation to slope, indicating a combined variability in slope and aspect. Predictor of habitat suitability and biodiversity.<br>VRM=0 → no variation in terrain around target pixel<br>VRM=1 → complete variation in terrain around pixel	No unit	<u>Radius</u> = 1 cell<br><br>(Sappington et al., 2007)
Bathymetry Position Index	**BPI**	*Morphometry*. Indicates a pixel's relative position with regards to surrounding pixels, giving an indication of local topography (peaks and troughs). Calculated using an annulus-shaped focal window. Predictor of habitat suitability and biodiversity.<br>BPI<0 → local lows in terrain (pits)<br>BPI=0 → flat terrain<br>BPI>0 → local highs in terrain (peaks)	Meters	<u>w</u>= c(1, 5); <u>stand</u> = none, <u>unit</u> = cell (*MultiscaleDTM* R package)<br><br>(Lundblad et al, 2006)

Variable	Abbv.	Description	Units	Parameters/Reference
Sky view factor	**SVF**	*Lighting*. Quantifies the proportion of sky visible from a point around a particular radius, by calculating the ratio of the radiation received by a planar surface to the radiation emitted by the entire hemispheric environment. It uses only the zenith angles above the horizontal plane.<br>SVF=0 $\rightarrow$ light completely obstructed to target pixel<br>SVF=1 $\rightarrow$ light completely unobstructed to target pixel	No unit	Max search radius = 10000; Method = sectors; Multi Scale Factor = 3; Number of sectors = 8<br>(Boehner et al, 2009, Hantzschel et al 2005, Oke 2000)

References

Böhner, J., & Antonić, O. (2009). Land-surface parameters specific to topo-climatology. In T. Hengl & H. I. Reuter (Eds.), *Developments in Soil Science* (Vol. 33, Issue C, pp. 195–226). Elsevier B.V. https://doi.org/10.1016/S0166-2481(08)00008-1

Du Preez, C. (2015). A new arc–chord ratio (ACR) rugosity index for quantifying three-dimensional landscape structural complexity. *Landscape Ecology*, *30*, 181–192. https://doi.org/10.1007/s10980-014-0118-8

Häntzschel, J., Goldberg, V., & Bernhofer, C. (2007). GIS-based regionalisation of radiation, temperature and coupling measures in complex terrain for low mountain ranges. *Meteorological Applications*, *12*, 33–42. https://doi.org/10.1017/S1350482705001489

Jenness, J. S. (2004). Determining surface area from DEM. *Wildlife Society Bulletin*, *32*(3), 829–839. https://doi.org/10.2193/0091-7648(2004)032[0829:CLSAFD]2.0.CO;2

Kalbermatten, M., Van De Ville, D., Turberg, P., Tuia, D., & Joost, S. (2012). Multiscale analysis of geomorphological and geological features in high resolution digital elevation models using the wavelet transforms. *Geomorphology*, *138*, 352–363. https://doi.org/10.1016/j.geomorph.2011.09.023

Koethe, R., & Lehmeier, F. (1996). *SARA - System zur Automatischen Relief-Analyse. User Manual, 2. Edition*. Dept. of Geography, University of Goettingen.

Lundblad, E. R., Wright, D. J., Miller, J., Larkin, E. M., Rinehart, R., Naar, D. F., Donahue, B. T., Anderson, S. M., & Battista, T. (2006). A benthic terrain classification scheme for American Samoa. *Marine Geodesy*, *29*(2), 89–111. https://doi.org/10.1080/01490410600738021

Oke, T. R. (2000). *Boundary Layer Climates*. Taylor & Francis.

Sappington, J. M., Longshore, K. M., & Thompson, D. B. (2007). Quantifying landscape ruggedness for animal habitat analysis: a case study using bighorn sheep in the Mojave Desert. *Journal of Wildlife Management*, *71*(5), 1419–1426. https://doi.org/10.2193/2005-723

Yokoyama, R., Shirasawa, M., & Pike, R. J. . (2002). Visualizing topography by openness: A new application of image processing to digital elevation models. *Photogrammetric Engineering and Remote Sensing, 68*, 257–265.

Zevenbergen, L. W., & Thorne, C. R. (1987). Quantitative analysis of land surface topography. *Earth Surface Processes and Landforms*, *12*(1), 47–56. https://doi.org/10.1002/esp.3290120107

Table S4. Summary results ranking MaxEnt species distribution models to determine optimal parameters of feature class (FC: L = linear only; LP = linear and product; LQ = linear and quadratic; LPQ =linear, product and quadratic) and regularization multipliers (RM: 1, 2, 5 and 10). Each *Acropora* species was assessed separately: (**a**) *A. hyacinthus*, (**b**) *A. spathulata*, and (**c**) *A. kenti*. *Acropora* distributions were predicted based on coral presence-only points (*A. hyacinthus*, n=329; *A. spathulata*, n=269; *A. kenti*, n=324) and 72 predictor variables used as background points (nine variables each from eight combinations of bathymetry sources and spatial resolutions). Each MaxEnt model was run using a leave-one-out cross validation method to select occurrence points from all but one reef at a time to train the model and use the left-out reef to test it. Background points were randomly selected as 75% training points, 25% testing points over three iterations for each cross-validation. Mean diagnostic values are shown here. Models were assessed using the mean Area Under the Receiver Operating Curve based on the test data (AUC_{TEST}), as well mean Bayesian Information Criterion (BIC). For each site, FC-RM combination was ranked by AUC_{TEST} and BIC separately, then determined the optimal FC-RM combination as the model resulting in the lowest sum of these ranks (Overall Rank). The top three ranked models at each site are highlighted in yellow, and the selected parameter of LQ 2 is in bold red font.

(a) *A. hyacinthus*

		AUC_{TEST}		BIC		Rank	Rank	Sum of	Overall
FC	RM	mean	sd	mean	sd	AUC_{TEST}	BIC	ranks	Rank
L	1	0.873	0.153	4776.8	156.7	16	5	21	8
L	2	0.882	0.144	4800.2	159.1	15	6	21	8
L	5	0.893	0.133	4834.8	160.8	14	9	23	9
L	10	0.904	0.115	4861.0	164.0	13	10	23	9
LP	1	0.912	0.090	5480.0	159.8	12	15	27	10
LP	2	0.921	0.086	5134.0	158.7	9	14	23	9
LP	5	0.927	0.086	4864.4	160.3	5	11	16	6
LP	10	0.920	0.094	4805.5	151.1	10	7	17	7
LQ	1	0.924	0.096	4688.5	147.7	8	1	9	2
**LQ**	**2**	**0.927**	**0.094**	**4690.6**	**149.4**	**4**	**2**	**6**	**1**
LQ	5	0.928	0.098	4720.0	158.9	3	3	6	1
LQ	10	0.925	0.103	4774.2	166.5	6	4	10	3
LPQ	1	0.917	0.085	5496.5	166.8	11	16	27	10
LPQ	2	0.929	0.076	5122.1	163.0	2	13	15	5
LPQ	5	0.932	0.077	4871.5	157.8	1	12	13	4
LPQ	10	0.924	0.086	4812.8	154.2	7	8	15	5

(b) ***A. spathulata***

FC	RM	AUC$_{TEST}$ mean	AUC$_{TEST}$ sd	BIC mean	BIC sd	Rank AUC$_{TEST}$	Rank BIC	Sum of ranks	Overall Rank
L	1	0.767	0.235	3955.8	146.3	16	9	25	9
L	2	0.796	0.196	3961.0	140.9	15	10	25	9
L	5	0.812	0.174	3971.1	145.6	14	11	25	9
L	10	0.817	0.151	4014.0	148.1	13	12	25	9
LP	1	0.837	0.178	4363.7	173.1	11	15	26	10
LP	2	0.848	0.166	4136.6	191.8	9	13	22	7
LP	5	0.849	0.153	3918.4	137.1	6	7	13	5
LP	10	0.849	0.152	3854.4	140.0	7	4	11	4
LQ	1	0.918	0.077	3743.8	132.1	2	1	3	1
**LQ**	**2**	**0.924**	**0.055**	**3768.6**	**136.4**	**1**	**2**	**3**	**1**
LQ	5	0.916	0.064	3844.2	140.7	3	3	6	2
LQ	10	0.904	0.083	3899.4	149.0	4	6	10	3
LPQ	1	0.835	0.176	4398.8	174.8	12	16	28	11
LPQ	2	0.848	0.166	4163.6	191.8	10	14	24	8
LPQ	5	0.849	0.152	3935.0	134.7	8	8	16	6
LPQ	10	0.849	0.150	3863.2	140.9	5	5	10	3

(c) *A. kenti*

FC	RM	AUC$_{TEST}$ mean	AUC$_{TEST}$ sd	BIC mean	BIC sd	Rank AUC$_{TEST}$	Rank BIC	Sum of ranks	Overall Rank
L	1	0.869	0.164	4593.7	92.4	16	5	21	9
L	2	0.882	0.150	4617.6	95.1	13	6	19	7
L	5	0.899	0.130	4663.1	95.6	10	9	19	7
L	10	0.904	0.118	4696.1	97.0	9	11	20	8
LP	1	0.873	0.165	5211.8	122.5	15	15	30	11
LP	2	0.883	0.156	4900.7	101.5	12	13	25	10
LP	5	0.911	0.097	4686.1	85.5	7	10	17	6
LP	10	0.929	0.066	4624.5	89.6	5	7	12	4
LQ	1	0.937	0.073	4496.3	81.9	4	1	5	2
**LQ**	**2**	**0.943**	**0.067**	**4512.8**	**88.7**	**3**	**2**	**5**	**2**
LQ	5	0.947	0.066	4525.1	83.5	1	3	4	1
LQ	10	0.944	0.068	4570.5	88.9	2	4	6	3
LPQ	1	0.875	0.163	5252.1	120.0	14	16	30	11
LPQ	2	0.883	0.156	4937.7	95.9	11	14	25	10
LPQ	5	0.910	0.098	4716.5	86.8	8	12	20	8
LPQ	10	0.928	0.066	4648.8	91.3	6	8	14	5

Table S5: Summary statistics of vertical error (Δh; in meters) of the bathymetry digital elevation models (DEM) at multiple spatial resolutions, derived from three publicly available sources: Allen Coral Atlas 15m, DeepReef 30m, and DeepReef 100m. Vertical error (Δh; in meters) was calculated as the difference between the tide-corrected depth of sampled colonies (n=2368) and the predicted depth from the bathymetry models. All values are in meters below sea level. Statistical measures that assume a normal distribution were recalculated with outliers removed, using an outlier threshold of 3 RMSE. *St dev* = standard deviation. *RMSE* = root mean square error. *NMAD* = normalized median absolute deviation. Q68.3% = 68.3% quantile of the absolute errors. Q95% = 95% quantile of the absolute errors.

Bathymetry source	**Allen Coral Atlas**				**DeepReef**			
Resolution (m b.s.l..)	**15**	**30**	**60**	**120**	**30**	**60**	**120**	**100**
Outliers (n)	40	13	3	23	32	29	17	9
Minimum	-4.18	-4.11	-21.72	-55.77	-7.03	-16.87	-50.59	-22.13
Maximum	20.44	20.92	19.64	17.90	38.58	38.63	38.07	37.05
Mean	2.72	3.74	4.95	4.79	4.35	5.61	6.75	7.60
Mean (outliers removed)	2.51	3.65	4.99	5.22	4.02	5.32	6.81	7.49
Mean absolute	2.83	3.86	5.65	8.06	4.41	5.99	9.52	9.98
Mean absolute *(outliers removed)*	2.62	3.77	5.63	7.75	4.09	5.70	9.29	9.88
St dev	2.74	3.18	4.82	8.93	3.91	4.98	9.56	9.28
St dev (outliers removed)	2.24	2.99	4.73	7.80	2.70	4.24	8.88	9.12
RMSE	3.86	4.90	6.91	10.13	5.85	7.50	11.70	12.00
RMSE (outliers removed)	3.36	4.72	6.87	9.37	4.83	6.80	11.19	11.80
Median (Q50%)	2.01	3.18	4.66	6.16	3.58	5.02	6.71	7.58
NMAD	1.75	2.66	4.97	6.55	2.23	3.87	7.35	6.53
Q68.3% (absolute)	3.18	4.42	7.64	10.57	4.88	7.08	12.12	11.79
Q95% (absolute)	7.60	9.90	11.97	17.40	9.87	12.21	22.30	24.82

Table S6. Formal comparisons of MaxEnt models built for three *Acropora* species: *A. hyacinthus* (n=329), *A. spathulata* (n=269), and *A. kenti* (n=324). Each model was evaluated using two metrics: the Area Under the Receiver Operating Curve based on the test data (AUC_{TEST}), and mean Bayesian Information Criterion (BIC). (**a**) Dunn's test of multiple comparisons using rank sums and (**b**) the Kruskal-Wallis test (non-parametric alternative to a one-way ANOVA) were used to identify differences in model performances between the three species, using the Holm p-value correction for multiple testing.

(a) Dunn's Test to perform pairwise comparisons between species, assessed for AUC_{TEST} and BIC metrics

	AUC_{TEST}			BIC		
	**Z-score**	***P*-value**	**Adj. *P*-value**	**Z- score**	***P*-value**	**Adj. *P*-value**
A. hyacinthus* – *A. spathulata	4.66	<0.001	<0.001 ***	55.67	<0.001	<0.001 ***
A. hyacinthus* – *A. kenti	-0.26	0.798	0.798	18.06	<0.001	<0.001***
A. spathulata* – *A. kenti	-4.72	<0.001	<0.001***	-36.45	<0.001	<0.001***

(b) Kruskal-Wallis test to identify differences in MaxEnt model performance between species, assessed for AUC_{TEST} and BIC metrics

		**Group**	**Minimum**	**Maximum**	**Q25**	**Q50**	**Q75**	**sd**
AUC_{TEST}	***A. hyacinthus***	a	0.507	0.999	0.853	0.948	0.978	0.10
	A. spathulata	b	0.278	0.998	0.865	0.926	0.966	0.11
	A. kenti	a	0.451	0.999	0.884	0.937	0.974	0.08
**BIC**	***A. hyacinthus***	a	4347.9	5339.8	4726.1	4845.8	4981.0	201
	A. spathulata	b	3477.9	4363.8	3809.7	3930.9	4046.9	177
	A. kenti	c	4295.1	5067.6	4526.8	4626.2	4776.4	167

Table S7. Formal comparisons of MaxEnt models built for three *Acropora* species: *A. hyacinthus* (n=329), *A. spathulata* (n=269), and *A. kenti* (n=324). Each model was evaluated using two metrics: the Area Under the Receiver Operating Curve based on the test data (AUC_{TEST}), and mean Bayesian Information Criterion (BIC).

Nine MaxEnt models using different input variables were built for each species. Eight input variables were derived from open-source bathymetric DEM models: Allen Coral Atlas (ACA) and DeepReef (DeepR), at one five spatial resolutions (15m, 30m, 60m, 100m, 120m), with a total of eight bathymetric source-resolution combinations. One model was built per bathymetric source-resolution combinations (e.g., ACA.15m, DeepR.30m, etc), with an additional model using all predictor variables at all spatial resolutions ('Allvars').

The models were evaluated individually for each species, where the input variables significantly impacted model performance between species: *A. hyacinthus* (AUC_{TEST}: $H(8)$= 155.30, $p<0.001$; BIC: $H(8)$= 688.78, $p<0.001$), *A. spathulata* (AUC_{TEST}: $H(8)$= 211.32, $p<0.001$; BIC: $H(8)$= 478.50, $p<0.001$), and *A. kenti* (AUC_{TEST}: $H(8)$= 171.10, $p<0.001$; BIC: $H(8)$= 1021.89, $p<0.001$) (**Supp Table S6**). Here, Kruskal-Wallis tests (non-parametric alternative to a one-way ANOVA) were used to perform pairwise comparisons between each model, assessing each species separately, using the Holm *p*-value correction for multiple testing.

Significant differences in metrics between compared models are highlighted as follows: * $p<0.05$; ** $p<0.01$; *** $p<0.001$.

	A. hyacinthus				*A. spathulata*				*A. kenti*			
**Comparison of AUC_{TEST} metric**	**Zscore**	***P*-value**	**Adj. *P*-value**		**Zscore**	***P*-value**	**Adj. *P*-value**		**Zscore**	***P*-value**	**Adj. *P*-value**	
ACA.15m – ACA.30m	-1.82	0.069	0.689		-1.50	0.134	1.000		-3.00	0.003	0.038	*
ACA.15m – ACA.60m	-1.68	0.093	0.841		1.23	0.219	1.000		-2.26	0.024	0.283	
ACA.15m – ACA.120m	5.76	<0.001	<0.001	***	7.29	<0.01	<0.01	***	4.56	<0.01	<0.01	***
ACA.15m – Allvars	-3.78	<0.001	0.003	**	1.66	0.096	0.867		-3.92	<0.01	0.002	**
ACA.15m – DeepR.30m	-0.35	0.724	1.000		3.13	0.002	0.028	*	1.06	0.287	1.000	
ACA.15m – DeepR.60m	-2.49	0.013	0.206		1.37	0.169	1.000		-2.69	0.007	0.092	
ACA.15m – DeepR.120m	3.46	<0.001	0.011	*	6.38	<0.01	<0.01	***	3.23	0.001	0.020	*

ACA.15m – DeepR.100m	2.45	0.014	0.217		9.28	<0.01	<0.01	***	4.08	<0.01	0.001	**
ACA.30m – ACA.60m	0.14	0.887	0.887		2.73	0.006	0.083		0.74	0.462	1.000	
ACA.30m – ACA.120m	7.58	<0.001	<0.001	***	8.79	<0.01	<0.01	***	7.56	<0.01	<0.01	***
ACA.30m – Allvars	-1.97	0.049	0.544		3.16	0.002	0.027	*	-0.92	0.357	1.000	
ACA.30m – DeepR.30m	1.47	0.143	1.000		4.63	<0.01	<0.01	***	4.06	<0.01	0.001	**
ACA.30m – DeepR.60m	-0.67	0.504	1.000		2.87	0.004	0.057		0.31	0.759	0.759	
ACA.30m – DeepR.120m	5.28	<0.001	<0.001	***	7.88	<0.01	<0.01	***	6.23	<0.01	<0.01	***
ACA.30m – DeepR.100m	4.26	<0.001	<0.001	***	10.78	<0.01	<0.01	***	7.08	<0.01	<0.01	***
ACA.60m – ACA.120m	7.44	<0.001	<0.001	***	6.06	<0.01	<0.01	***	6.82	<0.01	<0.01	***
ACA.60m – Allvars	-2.11	0.035	0.422		0.43	0.665	1.000		-1.66	0.097	0.975	
ACA.60m – DeepR.30m	1.32	0.186	1.000		1.90	0.058	0.636		3.33	<0.01	0.015	*
ACA.60m – DeepR.60m	-0.81	0.418	1.000		0.14	0.885	0.885		-0.43	0.668	1.000	
ACA.60m – DeepR.120m	5.13	<0.001	<0.001	***	5.15	<0.01	<0.01	***	5.49	<0.01	<0.01	***
ACA.60m – DeepR.100m	4.12	<0.001	<0.001	***	8.05	<0.01	<0.01	***	6.34	<0.01	<0.01	***
ACA.120m – Allvars	-9.55	<0.001	<0.001	***	-5.63	<0.01	<0.01	***	-8.48	<0.01	<0.01	***
ACA.120m – DeepR.30m	-6.12	<0.001	<0.001	***	-4.16	<0.01	<0.01	***	-3.49	<0.01	0.009	**
ACA.120m – DeepR.60m	-8.25	<0.001	<0.001	***	-5.91	<0.01	<0.01	***	-7.25	<0.01	<0.01	***
ACA.120m – DeepR.120m	-2.31	0.021	0.293		-0.91	0.364	1.000		-1.33	0.184	1.000	
ACA.120m – DeepR.100m	-3.32	<0.001	0.016	*	1.99	0.047	0.563		-0.48	0.632	1.000	
Allvars – DeepR.30m	3.43	<0.001	0.011	*	1.46	0.143	1.000		4.99	<0.01	<0.01	***
Allvars – DeepR.60m	1.30	0.195	1.000		-0.29	0.773	1.000		1.23	0.219	1.000	
Allvars – DeepR.120m	7.24	<0.001	<0.001	***	4.72	<0.01	<0.01	***	7.15	<0.01	<0.01	***
Allvars – DeepR.100m	6.23	<0.001	<0.001	***	7.61	<0.01	<0.01	***	8.00	<0.01	<0.01	***

DeepR.30m – DeepR.60m	-2.13	0.033	0.427		-1.75	0.080	0.797		-3.76	<0.01	0.003	**
DeepR.30m – DeepR.120m	3.81	<0.001	0.003		3.25	0.001	0.020	*	2.16	0.031	0.337	
DeepR.30m – DeepR.100m	2.80	0.005	0.087		6.15	<0.001	<0.001	***	3.01	0.003	0.039	*
DeepR.60m – DeepR.120m	5.94	<0.001	<0.001	***	5.01	<0.001	<0.001	***	5.92	<0.01	<0.01	***
DeepR.60m – DeepR.100m	4.93	<0.001	<0.001	***	7.90	<0.001	<0.001	***	6.77	<0.01	<0.01	***
DeepR.120m – DeepR.100m	-1.01	0.312	1.000		2.89	0.004	0.057		0.85	0.395	1.000	

**Comparison of BIC metric**	***A. hyacinthus***				***A. spathulata***				***A. kenti***			
	**Zscore**	***P*-value**	**Adj. *P*-value**		**Zscore**	***P*-value**	**Adj. *P*-value**		**Zscore**	***P*-value**	**Adj. *P*-value**	
ACA.15m – ACA.30m	4.45	<0.01	<0.01	***	3.22	0.001	0.013	*	7.8	<0.01	<0.01	***
ACA.15m – ACA.60m	4.14	<0.01	<0.01	***	1.26	0.208	1.000		5.4	<0.01	<0.01	***
ACA.15m – ACA.120m	-12.30	<0.01	<0.01	***	-7.06	<0.01	<0.01	***	-12.6	<0.01	<0.01	***
ACA.15m – Allvars	6.78	<0.01	<0.01	***	5.68	<0.01	<0.01	***	6.9	<0.01	<0.01	***
ACA.15m – DeepR.30m	-1.18	0.237	0.473		-1.20	0.228	0.913		-2.0	0.043	0.255	
ACA.15m – DeepR.60m	2.83	0.005	0.037	*	-0.41	0.683	0.683		5.6	<0.01	<0.01	***
ACA.15m – DeepR.120m	-8.10	<0.01	<0.01	***	-6.08	<0.01	<0.01	***	-9.4	<0.01	<0.01	***
ACA.15m – DeepR.100m	-6.82	<0.01	<0.01	***	-11.66	<0.01	<0.01	***	-9.9	<0.01	<0.01	***
ACA.30m – ACA.60m	-0.31	0.759	0.759		-1.97	0.049	0.345		-2.4	0.014	0.116	
ACA.30m – ACA.120m	-16.75	<0.01	<0.01	***	-10.28	<0.01	<0.01		-20.4	<0.01	<0.01	***
ACA.30m – Allvars	2.33	0.020	0.118		2.46	0.014	0.112		-0.9	0.345	1.000	
ACA.30m – DeepR.30m	-5.63	<0.01	<0.01	***	-4.43	<0.01	<0.01	***	-9.9	<0.01	<0.01	***
ACA.30m – DeepR.60m	-1.62	0.106	0.529		-3.63	<0.01	0.003	**	-2.2	0.028	0.193	
ACA.30m – DeepR.120m	-12.55	<0.01	<0.01	***	-9.30	<0.01	<0.01	***	-17.2	<0.01	<0.01	***

ACA.30m – DeepR.100m	-11.27	<0.01	<0.01	***	-14.88	<0.01	<0.01	***	-17.8	<0.01	<0.01	***
ACA.60m – ACA.120m	-16.45	<0.01	<0.01	***	-8.32	<0.01	<0.01	***	-18.0	<0.01	<0.01	***
ACA.60m – Allvars	2.64	0.008	0.058		4.42	<0.01	<0.01	***	1.5	0.133	0.665	
ACA.60m – DeepR.30m	-5.33	<0.01	<0.01	***	-2.46	0.014	0.124		-7.4	<0.01	<0.01	***
ACA.60m – DeepR.60m	-1.31	0.190	0.760		-1.67	0.096	0.575		0.2	0.808	0.808	
ACA.60m – DeepR.120m	-12.25	<0.01	<0.01	***	-7.33	<0.01	<0.01	***	-14.7	<0.01	<0.01	***
ACA.60m – DeepR.100m	-10.96	<0.01	<0.01	***	-12.92	<0.01	<0.01	***	-15.3	<0.01	<0.01	***
ACA.120m – Allvars	19.09	<0.01	<0.01	***	12.74	<0.01	<0.01	***	19.5	<0.01	<0.01	***
ACA.120m – DeepR.30m	11.12	<0.01	<0.01	***	5.86	<0.01	<0.01	***	10.6	<0.01	<0.01	***
ACA.120m – DeepR.60m	15.14	<0.01	<0.01	***	6.65	<0.01	<0.01	***	18.2	<0.01	<0.01	***
ACA.120m – DeepR.120m	4.20	<0.01	<0.01	***	0.98	0.325	0.974		3.2	0.001	0.012	*
ACA.120m – DeepR.100m	5.48	<0.01	<0.01	***	-4.60	<0.01	<0.01	***	2.7	0.008	0.071	
Allvars – DeepR.30m	-7.96	<0.01	<0.01	***	-6.89	<0.01	<0.01	***	-8.9	<0.01	<0.01	***
Allvars – DeepR.60m	-3.95	<0.01	<0.01	***	-6.09	<0.01	<0.01	***	-1.3	0.208	0.832	
Allvars – DeepR.120m	-14.89	<0.01	<0.01	***	-11.76	<0.01	<0.01	***	-16.2	<0.01	<0.01	***
Allvars – DeepR.100m	-13.60	<0.01	<0.01	***	-17.34	<0.01	<0.01	***	-16.8	<0.01	<0.01	***
DeepR.30m – DeepR.60m	4.02	<0.01	<0.01	***	0.80	0.426	0.851		7.7	<0.01	<0.01	***
DeepR.30m – DeepR.120m	-6.92	<0.01	<0.01	***	-4.87	<0.01	<0.01	***	-7.3	<0.01	<0.01	***
DeepR.30m – DeepR.100m	-5.64	<0.01	<0.01	***	-10.45	<0.01	<0.01	***	-7.9	<0.01	<0.01	***
DeepR.60m – DeepR.120m	-10.94	<0.01	<0.01	***	-5.67	<0.01	<0.01	***	-15.0	<0.01	<0.01	***
DeepR.60m – DeepR.100m	-9.65	<0.01	<0.01	***	-11.25	<0.01	<0.01	***	-15.6	<0.01	<0.01	***
DeepR.120m – DeepR.100m	1.28	0.199	0.597		-5.58	<0.01	<0.01	***	-0.6	0.556	1.000	
